# Amyloid-β plaque diversity across brain regions in Alzheimer’s disease and its relation to major clinicopathological traits

**DOI:** 10.1007/s00401-026-03096-1

**Published:** 2026-10-07

**Authors:** Antonia Neubauer, Paul Feyen, Susanna Pekrun, Sigrun Roeber, Viktoria Ruf, Felix L. Strübing, Jochen Herms

**Affiliations:** 1 Institute of Neuropathology, LMU Medizin, Ludwig-Maximilians-Universität München, Munich, Germany; 2 German Center for Neurodegenerative Diseases (DZNE), Munich, Germany; 3 Munich Cluster for Systems Neurology (SyNergy), Munich, Germany

**Keywords:** Alzheimer’s disease, Amyloid beta, Plaque morphology, Classification, Machine learning, Digital pathology

## Abstract

Amyloid beta (Aβ) deposition and tau accumulation are neuropathological hallmarks of Alzheimer’s disease (AD). While Aβ deposits present with a wide morphological variety, a comprehensive, cross-regional overview of Aβ plaque composition in AD and associations with clinicopathological traits are missing. In this study, we systematically documented the abundance and morphological diversity of Aβ plaques in up to 23 brain regions of 84 advanced AD cases. Deposits were segmented with a random forest pixel classifier in 4G8 diaminobenzidine stains and classified into six classes using a convolutional neural network (cored, diffuse light, diffuse dense, compact, small dense plaques, cerebral amyloid angiopathy (CAA)). Deposit counts were correlated between classes, regions, and tau load using Pearson correlation. Associations between plaque densities and co-pathologies, sex, ApoE, familial vs. sporadic cases, age at clinical onset, disease duration, and age at death were examined. Our classification model achieved a recall of 81.5% and a precision of 82.4% in the test set. Cortical brain regions showed the highest Aβ plaque loads, predominantly consisting of diffuse light and small dense plaques. Plaque densities correlated within and across the hippocampal and cortical regions. Abundances of diffuse dense, small dense, and compact plaques were positively correlated with one another, while densities of cored plaques and CAA did not correlate with other plaque classes. Only cored plaques were positively correlated with the entorhinal tau load, while other plaque classes, except CAA, showed negative correlations. Notably, female sex was associated with more diffuse plaques, and ApoE4 was associated with diffuse dense and small dense plaques. Earlier age at onset and death were associated with higher densities of diffuse light plaques. Overall, associations varied across brain regions and plaque classes. Cored plaques and CAA stand out statistically from other Aβ deposits, suggesting distinct association patterns in the course of the disease.

**Supplementary Information:**

The online version contains supplementary material available at https://doi.org/10.1007/s00401-026-03096-1.

## Introduction

Amyloid beta (Aβ) plaques, alongside hyperphosphorylated tau deposition, are the neuropathological hallmarks of Alzheimer’s disease (AD) [12, 44, 82], the most common form of dementia [43, 44]. Aβ, a cleavage product of the amyloid precursor protein (APP), accumulates mostly in the extracellular space in the brain and spreads via misfolding and seeded protein aggregation [38, 53, 82]. The aggregation usually begins in the associative cortices and appears in subcortical areas, the brainstem, and the cerebellum at later stages, according to Thal phases 1 to 5 [73]. Thereby, Aβ is seen as an initiator of the AD-related pathology, preceding tau pathology, synapse and neuron loss, and cognitive decline [5, 32, 82]. This view is strongly supported by the fact that familial cases of Alzheimer’s disease involving an *APP* or *PSEN1/2* mutation exhibit altered Aβ metabolism [82]. Although the general Aβ load does not exclusively account for all ongoing processes in dementia [21], numerous therapeutic approaches are directed against Aβ deposition with varying degrees of success [5, 30, 95].

A complicating factor is that Aβ deposition is highly heterogeneous [40, 72, 82, 85]. The deposits range from frequently described diffuse plaques, classical cored plaques, cerebral amyloid angiopathy (CAA) [72, 82] and distinct plaque types, such as cotton-wool plaques [35, 45] and coarse-grained plaques [9, 10]. Further deposits without explicit names were observed as subpial bands, stellate-shaped deposits, small puncta, and intracellular inclusions [82]. Besides plaques, Aβ also appears in toxic, small oligomeric species, which may partly be derived from larger deposits or generated intracellularly [31, 78, 83].

Diverse morphologies can reflect local tissue structure, altered molecular compositions, and are associated with varying determinants and reactions [27, 40, 72, 82]. The AD risk factor ApoE4 for example, is associated with generally higher cortical Aβ loads [58, 63], including higher loads of Aβ oligomers [34]. Female sex was also related to higher Aβ loads [1, 57, 58], although not in every study [6]. While diffuse Aβ plaques commonly occur in aging without cognitive impairment [20, 24], cored plaques were associated with cognitive decline [47, 50]. Especially neuritic cored plaques are known for an accompanying immune reaction with microglia infiltration [67]. Moreover, it has been suggested that some plaques undergo a progression from diffuse deposits to more fibrillar and compact to classic cored plaques, and finally to ‘burned-out’ plaques lacking a diffuse ring [62, 72, 82], while other studies suggest distinct factors influencing the plaque morphology [33, 48].

While many papers discuss individual components of specific Aβ plaques in a few brain regions, a comprehensive overview, quantifying and classifying Aβ plaques across brain regions in a large human AD cohort, is missing. For a large-scale approach, automated analysis is necessary. In recent years, several algorithms for Aβ plaque segmentation [58, 81] and classification were developed, achieving high accuracies [3, 40, 60, 71, 80, 89]. However, current light microscopy plaque classifiers are often limited to a subset of classes, which is not representative of broader, real compositions [71, 89].

In this study, Aβ deposits from up to 23 brain regions of a large cohort of 84 advanced familial and sporadic AD cases were extracted from immunohistochemical Aβ stains. Building upon the convolutional neural network by Tang et al. [71], we trained a more class-comprehensive algorithm: plaques were classified into six classes, namely cored, diffuse light, diffuse dense, compact, small dense plaques, and CAA. Absolute plaque densities and relative class loads were mapped across the brain, checked for correlations, and examined for associations with tau, alpha-Synuclein (α-syn) and TDP43 co-pathology. Furthermore, associations with sex, ApoE genotype, familial AD mutations, age at clinical disease onset, disease duration, and age at death were investigated. Finally, a cross-factor statistical model was developed to integrate various dimensions to identify Aβ deposit class-specific determining factors.

## Materials and methods

### Human cohort and neuropathological assessment

Human brain samples were acquired from the Neurobiobank Munich, which collects brains in accordance with the Code of Conduct of Brain-Net Europe [39] and in agreement with the local ethics committee. Brains were collected from voluntary donors after informed consent by the donor when alive or closest relatives conforming with the presumed will of the donor. This study follows the principles of the Declaration of Helsinki and is in accordance with the local ethics committee. At least two board-certified neuropathologists conducted the neuropathological assessments.

Inclusion criteria for this study were 1) registration of the case in the digital brain bank form as advanced, neuropathologically defined AD (Braak and Braak stage IV, V, or VI) and 2) availability of scanned diaminobenzidine (DAB) Aβ stains. Cases with other suspected clinical diagnoses, but AD-related changes in the neuropathological examination and cases with co-pathologies were also included. These criteria resulted in a cohort of 84 AD cases (Table 1, Fig. S1).

**Table 1:** Cohort description

	Available n (% of the cohort)	Cohort (absolute)	Cohort (%)
*n* (%)	84 (100%)	84	100%
Clinical diagnosis (AD: FTD: PD)^a^	84 (100%)	51: 7: 6	61%: 8%: 7%
Sex (female: male)	84 (100%)	46: 38	55%: 45%
Age at onset [years]	70 (83%)	61.6 ± 12.2	
Disease duration [years]	70 (83%)	10.9 ± 5.9	
Age at death [years]	83 (99%)	73.9 ± 11.4	
Braak and Braak (IV: V: VI)	84 (100%)	10: 15: 59	12%: 18%: 70%
Thal phase (3: 4: 5)	79 (94%)^b^	2: 10: 67	3%: 13%: 85%
CERAD (B: C)	73 (87%)	6: 67	8%: 92%
ApoE ((E2/E3): (E2/E4): (E3/E3): (E3/E4): (E4/E4))	78 (93%)	4: 1: 28: 38: 7	5%: 1%: 36%: 49%: 9%
WGS (no mutation: APP: PSEN1: PSEN2: TREM2)	70 (83%)	50: 3: 13^c^: 3: 1	71%: 4%: 19%: 4%: 1%
α-syn (neg: amygdala: brainstem: cortical)	71 (85%)	29: 15: 5: 22	41%: 21%: 7%: 31%
TDP43 (neg: pos)^d^	60 (71%)	32: 28	53%: 47%

*AD* Alzheimer’s disease; *PD* Parkinson’s disease; *FTD* Frontotemporal dementia; *WGS* whole genome sequencing

^a^For clarity, only the most common clinical diagnoses are listed here. The complete diagnoses list for the cohort can be found in Fig. S1a

^b^Regarding the five missing cases, four have a Thal phase ≥3 and one case ≥2

^c^In one patient both, a PSEN1 and a TREM2 mutation, were found. Only the PSEN1 mutation is listed here

^d^Binary values of the TDP43 status, based on the assessment of amygdala, hippocampus and medulla oblongata where available, were extracted from the biobank database

Formalin-fixed and paraffin-embedded tissue was manually sliced into 5 µm thick slices. Further processing was conducted in a Ventana Bench-Mark Ultra system (Roche). Slices were pretreated with 80% formic acid for 15 min and Cell Conditioning (CC1) Tris-based buffer (Roche). The monoclonal antibody, clone 4G8 (BioLegend, #800,701; dilution 1:5000), was applied as primary antibody for Aβ staining with a Ventana antibody dilution buffer (Roche, #251–018). Detection was conducted with an ultraView Universal DAB Detection Kit (#760–500, Roche), and hematoxylin and bluing reagent (Roche) was used for nuclear counterstain. Details about the staining for phosphorylated tau with the monoclonal antibody AT8 (ThermoFisher, #MN1020; dilution 1:400) and for α-syn with a monoclonal antibody (clone 42; BDTransduction, #610,787; dilution 1:1000) staining were described elsewhere before [58].

**Fig. 1:**
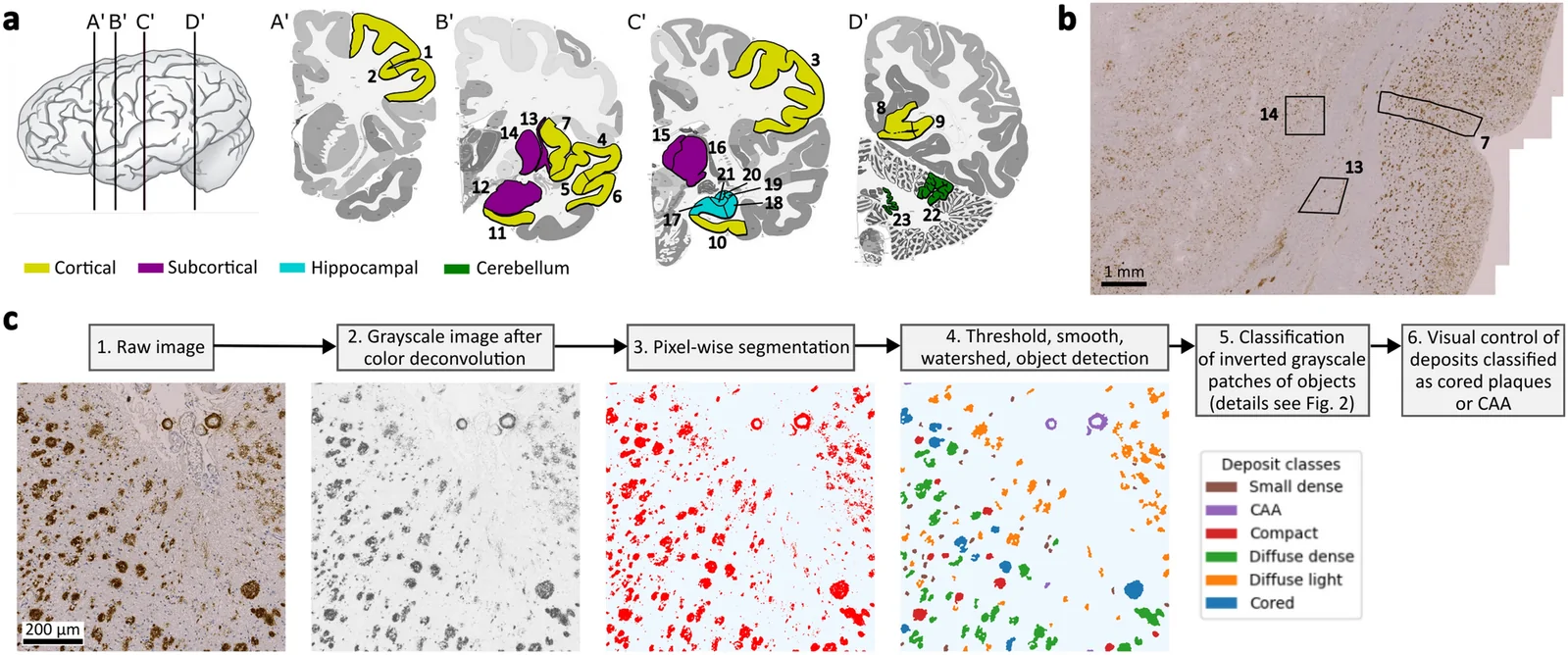
Region annotation and Aβ deposit quantification. **a** 23 brain regions were included in the analysis, comprising eleven cortical, five subcortical, five hippocampal, and two cerebellar regions. Regions are labeled in coronal slices of the gyral Allen Human Brain Atlas (slices A’ to D’: 18, 34, 55, and 83), adapted from [58] under the CC BY 4.0 license [2, 26, 58]. **b** In every region, a representative area was selected following a standardized protocol (Table S1). **c** The raw immunohistochemical image (1) was preprocessed with color deconvolution (2), and deposits were detected by a random-forest pixel classifier with ilastik (3). Segmentation masks were processed by thresholding, smoothing, watershed and object detection (4). Aβ deposits were extracted from the inverted grayscale image based on these masks, classified into six deposit classes with a convolutional neural network (5) and manually reclassified where necessary (6). 1 middle frontal gyrus, 2 sulcus of middle frontal gyrus, 3 parietal gyrus, 4 superior temporal gyrus, 5 sulcus between superior and middle temporal gyrus, 6 middle temporal gyrus, 7 insular gyrus, 8 striate area (occipital) gyrus, 9 striate area (occipital) sulcus, 10 parahippocampal gyrus, 11 entorhinal cortex, 12 amygdala, 13 claustrum, 14 putamen, 15 medial thalamus, 16 lateral thalamus, 17 subiculum, 18 CA1 of hippocampus, 19 CA2, 20 CA3, 21 CA4, 22 cerebellar cortex, 23 dentate nucleus, CAA cerebral amyloid angiopathy

Depending on the availability of Aβ stains across AD cases of the Neurobiobank Munich, up to 23 brain regions per case were included for further analysis (Fig. 1, Fig. S2). The regions comprise eleven cortical regions, five subcortical regions, CA1-4 and subiculum as hippocampal subregions, and the cerebellar cortex as well as the dentate nucleus of the cerebellum.

The ApoE genotype and AD-related mutations were extracted from whole genome sequencing data, described elsewhere before [58, 69]. In short, DNA was isolated from fresh frozen cerebellar tissue with the QIAmp DNA Mini Kit (Qiagen, 51,304). The TruSeq PCR-free genomic DNA library prep kit (Illumina, FC-121-3003) was used for library preparation. Sequencing of 2×150 bp paired-end libraries was conducted on an Illumina NovaSeq machine to a depth of at least 35X. A Snakemake pipeline incorporating the GATK best practices was used for alignment and variant calling. Quality control was done with FastQC. After adapter trimming, BWA-MEM2 was used for alignment to the hs1/T2T genome assembly (chm13v2.0). Variant calling, recalibration, and joint genotyping were performed with GATK (version 4.0). The ApoE specific variants (rsID/hs1 coordinates: rs429358/chr19:47,733,380; rs7412/chr19:47,733,518) were gathered to determine the ApoE genotype.

### Image analysis and deposit detection

Image acquisition and initial deposit detection were conducted as previously described [58]. In short, Aβ stains were digitized using a Zeiss Axio Scan Z.1 scanner with a 20× magnification. Following a standardized protocol (Table S1) [58], gray matter regions were annotated manually in QuPath (version 0.5.1) [4] in α-synuclein stains of the same samples where available and were registered onto Aβ and tau stains with non-rigid co-registration by DeeperHistreg in Python (Python version 3.10.12) [58, 86–88]. Cortical regions were annotated with rectangles spanning from the white matter to the cortex surface to consistently cover all layers. Annotations were corrected if necessary to avoid artifacts or large blood vessels. Standardized sizes for cortical regions, around 1 mm^2^, and non-cortical regions, around 0.7 mm^2^, were aimed for. In the implementation, the annotation sizes vary to a limited degree due to anatomical conditions, such as small regions of interest and the precise plane of sectioning during autopsy (see Table S1 for details).

For further computations, the annotated regions were partitioned into tiles of 4096*4096 pixels (900*900 µm^2^). As part of the preprocessing, Aβ and tau tiles underwent color-deconvolution to extract the brown DAB signal and conversion to grayscale. A random forest pixel classifier, trained in ilastik (version 1.4.0) for Aβ deposit detection and a second pixel classifier for tau detection [58], were applied to these grayscale images, resulting in probability maps. Deposit segmentations were extracted by thresholding the probability maps with 0.7 for both Aβ and tau. Subsequently, Aβ and tau covered area were calculated as the ratio of the segmented area divided by the whole area of the annotated region of interest.

**Fig. 2:**
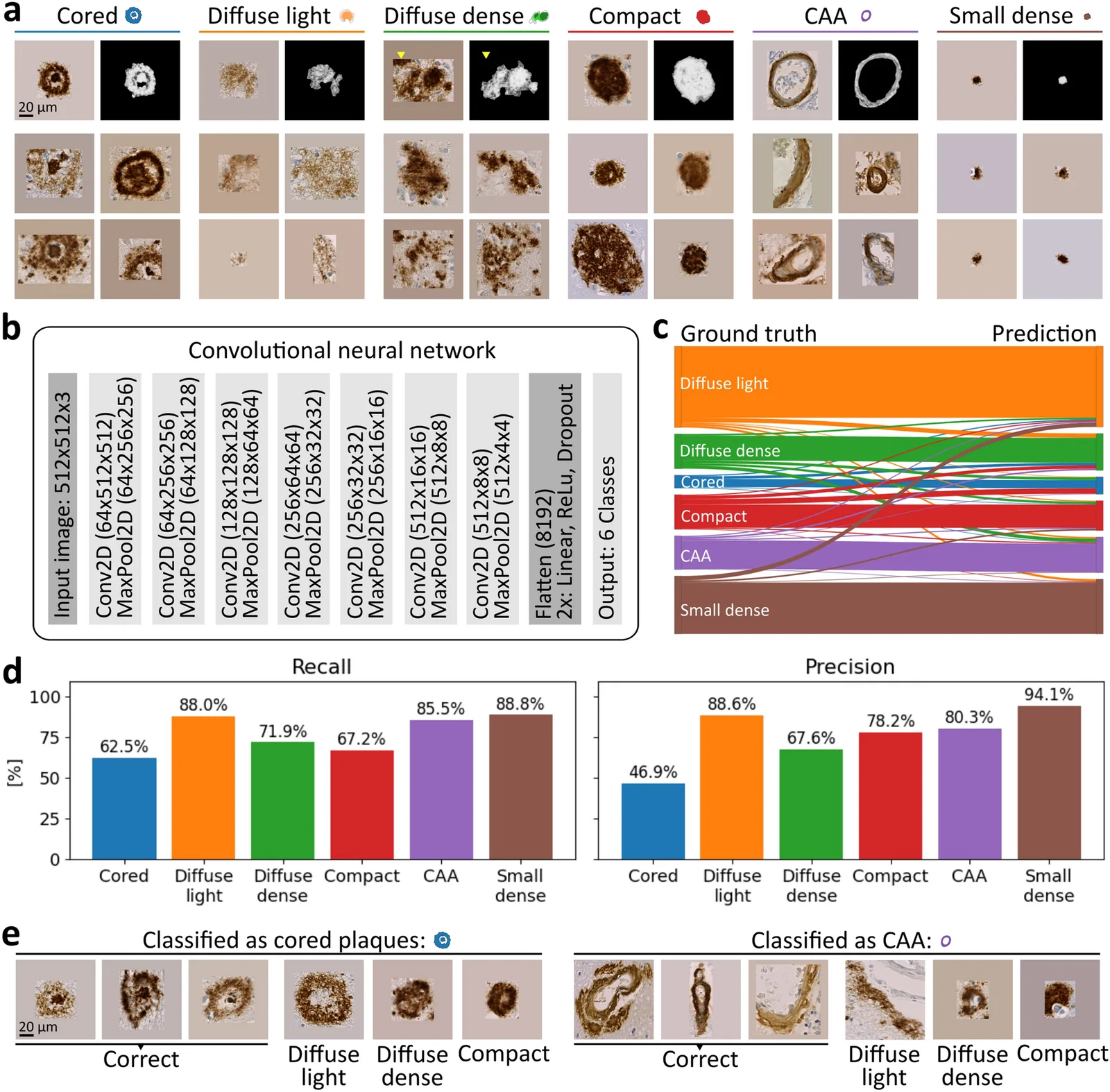
Aβ plaque classification. **a** Exemplary immunohistochemical images of six Aβ plaque classes. In the top row, exemplary images are shown with the related inverted gray scale images, which were used as inputs for the classification model. Rows two and three show four further examples of each class. The yellow arrowhead in the image of a diffuse dense plaque indicates that background deposits that do not belong to the main deposit of interest are removed for the classification of grayscale images. **b** Architecture of the convolutional neural network for the classification of standardized images into six classes. **c** Sankey plot to visualize the performance of the model in the test set. Diagonal links illustrate false predictions. **d** Recall (true positives / (true positives + false negatives)) and precision (true positives / (true positives + false positives)) of the model for each class in the test set, matching the data of the Sankey plot. **e** Example images of correctly and misclassified deposits. CAA cerebral amyloid angiopathy

To extract individual Aβ deposits, the deposit segmentation mask was passed through a smoothing step to capture specifically cored and diffuse plaques in one piece each. E.g., the dense core and the more diffuse surrounding Aβ positivity of a cored plaque should be detected as a single, continuous plaque instead of two separate components. As this step leads to a conflation of moderately defined, diffuse plaques, a watershed step was added for disproportionately large deposits (> 80,000 pixels, which equals 3865 µm^2^). Small deposits were removed by a size threshold of (< 2070 pixels, which equals 100 µm^2^) because they mostly cannot be meaningfully differentiated, and to reduce the computational workload. Deposits that were located outside of the annotated area of interest with more than 50% of their deposit area were excluded as external deposits. The thus defined object masks (Fig. 1c) were applied to the underlying grayscale or color image to extract 512*512-pixel squares (Fig. 2a), centered on the object mask. Deposits were captured from grayscale images for machine learning-based classification and from raw color images for manual annotation. If deposits were larger, they were scaled down to fit the standardized square size. Adjacent deposits that were accidentally depicted on the squares, although they did not correspond to the target deposit, were removed from the grayscale images.

### Aβ deposit classification

While former models, including the model by Tang et al. [71], concentrated on three classes, namely cored, diffuse and CAA, we aimed to distinguish more classes: a) to capture and describe a wider range and heterogeneity of plaques and b) to be able to meaningfully classify all detected deposits. The classes were defined based on descriptions in the literature and morphologically reproducible distinguishability. Preliminary attempts to manually classify the deposits eventually led to a distinction between cored plaques, diffuse light plaques, diffuse dense plaques, compact plaques, CAA, and small dense plaques. Descriptions are listed in Table 2. Deposits for training the classifier were extracted from a selection of whole image tiles of the study cohort that contained region annotations in at least parts of the tile. These tiles were visually chosen to cover a wide range of morphologies. Particular attention was paid to ensuring a diversity of cored plaques and CAA, which would otherwise be even more underrepresented in terms of numbers. Eventually, the training set was partially overlapping, but not completely a subset of the later evaluated deposits. In total, 3139 deposits from training images of 52 patients across regions were manually classified by one rater (AN) with visual confirmation by board-certified neuropathologists. While the manual classification by one primary rater introduced a bias in the classifier, this bias remained consistent across the deposit evaluation and later correction. In spite of preliminary plaque definitions and accompanying reference images, an unambiguous classification of many deposits is not possible; consistency despite subjective categorization is a realistic approximation to this subtle task. 15% of the deposits from every class were randomly assigned to the test set, while the remaining 85% were assigned to the training set. From the latter, 20% were randomly chosen for validation during hyperparameter optimization (Table 3).

**Table 2:** Overview of deposit classes and their descriptions

Class	Description
Cored	Classic plaque with dense core, free or poor ring, and denser, often diffuse rim
Diffuse light	Diffuse plaque with mostly low intensity and without dense centers; often with indistinct edges; also includes many pial and subpial deposits
Diffuse dense	Diffuse plaque with dense, irregular centers, and indistinct edges
Compact	Compact plaque with comparably homogeneous intensity and mostly sharp border; includes cotton wool and coarse-grained plaques
CAA	Cerebral amyloid angiopathy: often circular Aβ deposits in blood vessel walls or parts of blood vessel walls
Small dense	Small, mostly dense and often round deposit, partly intracellular Aβ deposit

*CAA* cerebral amyloid angiopathy

**Table 3:** Composition of the training, validation, and test set with class-wise and general performance of the convolutional neural network

Class	*n* (all)	*n* (train)	*n* (validation)	*n* (test)	Recall (test)	Precision (test)
Cored	161	108	29	24	62.5%	46.9%
Diffuse light	999	688	161	150	88.0%	88.6%
Diffuse dense	424	291	69	64	71.9%	67.6%
Compact	430	290	76	64	67.2%	78.2%
CAA	412	280	70	62	85.5%	80.3%
Small dense	713	478	128	107	88.8%	94.1%
Total	3139	2135	533	471	81.5%	82.4%

Recall (true positives / (true positives + false negatives)); precision (true positives / (true positives + false positives)); *CAA* cerebral amyloid angiopathy

While classes were balanced with additional data augmentation for training, the test set remained imbalanced, representing more realistic distributions. For the actual model application, apparent false-positive predictions for the classes ‘cored plaques’ and ‘CAA’ were corrected by visual adjudication, while false negatives assigned to other classes could not be systematically recovered

In relation to the underrepresentation of each class, the training images of the classes were expanded by data augmentation, including rotation, flipping, color jitter, shifting, shearing and scaling, resulting in a class-balanced training.

Appropriate to the task of morphological classification of images, a convolutional neural network (CNN) was created, based on the model architecture of the high-performance Aβ plaque classification from Tang et al. [71]. Due to the increased number of classes, a convolutional layer was added to the architecture implemented by Tang et al. [71] to represent the higher complexity. Additionally, the parameters were adapted to the input size of 512*512 pixels. The resulting structure includes seven blocks of 2D convolutional layers with 3×3 kernels and accompanying max-pooling for size reduction (Fig. 2). Model parameters were optimized by a cross-entropy loss.

The CNNs were further optimized regarding batch size, number of epochs, learning rate, severity of data augmentation, and distinction of diffuse plaques (Fig. S3). Experiments revealed comparable performance with batch sizes of 18 and 24, stabilization of parameters until epoch 60, and best performance with a learning rate of 0.0001 and moderate data augmentation. Moderate data augmentation with torchvision (version 0.18.1) included random rotation, horizontal and vertical flip, color jitter (transforms.ColorJitter(brightness=0.1, contrast=0.1, saturation=0.1, hue=0.02)), and further low-grade geometric augmentations (transforms.RandomAffine(0, translate=(0.05, 0.05), scale=(0.95, 1.05), shear=10)). Additionally, the distinction of two classes for diffuse plaques, namely lighter and denser diffuse plaques, did not lead to deterioration, but rather to an equal or improved overall performance. After preselection of three candidate models with the validation set, a final model was chosen with the highest performance in the holdout test set (Table 3). This model was trained with a batch size of 24 for 56 epochs with moderate data augmentation, optimal learning rate, and distinction of six classes.

Code and models, including the ilastik random forest pixel classifier and the convolutional neural network parameters for deposit classification, are publicly available on GitHub (https://github.com/cor2ni/Abeta_histo_classifier).

### Statistical analysis

Brain regions were compared regarding absolute deposit loads and deposit class proportions, applying Kruskal–Wallis tests and Dunn’s post hoc analyses with FDR Benjamini–Hochberg correction for multiple testing. To evaluate the effect of uneven area sampling between subjects, i.e., not every brain region was available for Aβ staining in every subject, we introduced a variable called *complete subset*, for 40 subjects which completed staining in nine selected brain regions (frontal, occipital, hippocampal and cerebellum regions). The Kruskal–Wallis tests and Dunn’s post hoc analysis with FDR correction were repeated in this complete subset. Additionally, Wilcoxon signed-rank tests with FDR correction were applied in the complete subset to account for paired measurements by patient IDs. Furthermore, we applied multiple linear regression models for each of these nine brain regions separately: total plaque counts, absolute and relative class-wise plaque counts were dependent variables; *complete subset* was the independent variable; and age at death, sex and ApoE genotype were covariates. This allowed us to assess whether this complete subset behaves differently from the overall cohort.

To examine correlations between deposit classes across cortical regions, the absolute and percental load of each class was correlated with the overall load of Aβ deposits in the same region by Pearson correlation with subsequent FDR correction, after accounting for inherent region differences by linear regression. As a control analysis to correct for within-patient dependence, *patient ID* was added next to the *region* in the linear regression model. The whole approach was repeated to correlate absolute and percental loads of each class with the absolute and percental loads of the other classes. Respective results were visualized as heatmaps. As an exemplary, region-specific visualization, absolute and relative Aβ deposit classes of the middle frontal gyrus were correlated with each other using Pearson correlation and visualized as scatter plots. The approach was repeated for correlating Aβ classes across cortical regions with the entorhinal tau covered area, presented as a heatmap, and for correlating Aβ classes in the middle frontal gyrus with the entorhinal tau covered area, presented as scatter plots.

To investigate the associations between Aβ deposit classes and putatively predicting or affected variables, multiple linear regression models were applied across cortical regions. In detail, the absolute (or relative) deposit class loads were the dependent variable. The independent variables of interest were Braak and Braak stage as a measurement for tau (additional correction for age at death, sex and ApoE genotype), α-syn co-pathology (additional correction for age at death, sex and ApoE genotype), TDP43 co-pathology (additional correction for age at death, sex and ApoE genotype), sex (additional correction for age at death and ApoE genotype), ApoE genotype (additional correction for age at death and sex), genetic AD (additional correction for sex and ApoE genotype), age at clinical disease onset (additional correction for sex and ApoE genotype), disease duration (additional correction for age at death, sex and ApoE genotype) and age at death (additional correction for sex and ApoE genotype). Additionally, all models included the region name as a categorical covariate to correct for brain region differences. P-values were adjusted with FDR Benjamini–Hochberg correction for multiple testing of each independent variable comparison, respectively. As control analyses for within-patient dependence, these regression models were repeated as linear mixed-effects models with a random effect for patient ID. However, with subsequent FDR correction, there remained no significant results, suggesting an overcorrection. For this reason, and to avoid making the paper more difficult to read, only the results of the linear regression models are reported below.

To integrate several factors into a single analysis, linear mixed-effects models were created with deposits/mm^2^ of each class as the dependent variable; brain region, sex, ApoE4 status, age at onset, and disease duration as independent variables; and patient ID as a random effect:

Deposit class x ~ C(region) + C(sex) + C(ApoE4) + age at onset + disease duration + (1 | patient ID).

Categorical variables are indicated with a C(). *P*-values were adjusted with the FDR Benjamini–Hochberg correction for multiple testing across all linear mixed-effects models. Furthermore, multiple linear regression models were created for several exemplary regions with the class-wise deposit density as the dependent variable, and sex, ApoE4 status, age at onset and disease duration as independent variables. The significance level was set to *p* < 0.05 (*), *p* < 0.01 (**), and *p* < 0.001 (***) for all analyses. Plots and statistics were implemented with Python (version 3.10.12).

## Results

We disentangled the commonalities and heterogeneity of Aβ pathology across Alzheimer’s disease (AD) cases by automatically quantifying and classifying Aβ deposits across 84 humans, including up to 23 brain regions per case. In total, 57,233 Aβ deposits were extracted from immunohistochemical stains of 1179 annotated regions and assigned to six deposit classes (cored, diffuse light, diffuse dense, compact, CAA, small dense) using a convolutional neural network (CNN).

Readers should interpret the results with consideration of the dataset, which contains a recruitment bias based on voluntary brain donation (Table 1). Due to 29% of familial AD cases, the cohort has an average age at clinical disease onset of 61.6 ± 12.2 years and an average age at death of 73.9 ± 11.4 years. 55% of the cohort are female. All cases clinically presented with dementia. Most cases showed high Braak and Braak stages (V or VI) and a Thal phase 5. To provide reasonable templates for correlation analyses in future studies without disclosing individual data due to data protection, Table S19 supplies region and sub-cohort specific Aβ deposit counts per mm^2^. The respective column names are explained in Table S18.

### Aβ deposit classifier performance

The distinction of Aβ deposits into six classes (Fig. 2) is a compromise between a large number of distinguishable classes and a sufficiently high frequency for each class. While cored plaques and CAA are commonly known, diffuse plaques were further subdivided into diffuse light and diffuse dense plaques. Compact plaques were merged with rarer coarse-grained and cotton-wool plaques. Small dense plaques cover the remaining deposits. The respective contextualization within the literature is addressed in the discussion. The smallest (< 100 µm^2^) Aβ objects were excluded to keep the computational effort reasonable. For standardized, large-scale, quantitative classification of Aβ deposits, a convolutional neural network was trained and optimized (Fig. S3) to assign input images to six classes. The model was trained on 2135 images, validated on 533 images, and tested on a held-out test set of 471 manually classified images.

In the test set, the model achieved a high recall of 81.5% (true positives / (true positives + false negatives)) and a high precision of 82.4% (true positives / (true positives + false positives)) (Table 3). The recall was greater than 85% in three deposit classes, namely diffuse light, CAA, and small dense plaques. Diffuse dense and compact plaques were classified most of the time correctly with a recall of 71.9% and 67.2%. Cored plaques achieved the lowest recall with 62.5%, with most incorrect calls being classified as diffuse dense deposits. The precision was the highest regarding small dense deposits (94.1%) and diffuse light deposits (88.6%), which also represent the most common deposits. The model precision was medium high for CAA (80.3%), compact (78.2%), and diffuse dense plaques (67.6%). Cored plaques were detected with a relatively low precision of 46.9% in the test set. To improve the low precision in cored plaques and CAA, the two smallest classes, all deposits that were classified as cored plaques or CAA were visually checked and reclassified by one rater (AN) as needed. This included 2955 deposits labeled as cored plaques, of which 68.7% were manually reassigned to other classes, and 2276 deposits labeled as CAA, of which 82.7% were reassigned to other classes (Fig. S4). The difference in precision compared to the test set is likely due to the smaller proportion of cored plaques and CAA in the application dataset compared to the test set and a fraction of deposits with a morphology that is difficult to classify. These results underscore the importance of manual verification of predictions as cored plaques and CAA from our model, while the other classes were significantly larger in number and thereby more robust in the large-scale application. Furthermore, the fact that cored plaques can only be reliably identified if the core is in the sectional plane, is a limitation of the two-dimensional approach which leads to a general underestimation of this plaque type. The precision between subsets of classes likely also reflects ambiguity of morphologies. Overall, the model demonstrates solid and realistic classifications across a large dataset with some manual assistance. The advantages of the presented convolutional neural network lie in the distinction between six classes and in the fact that all detected deposits can be categorized into these classes.

### Regional differences in Aβ deposition

**Fig. 3:**
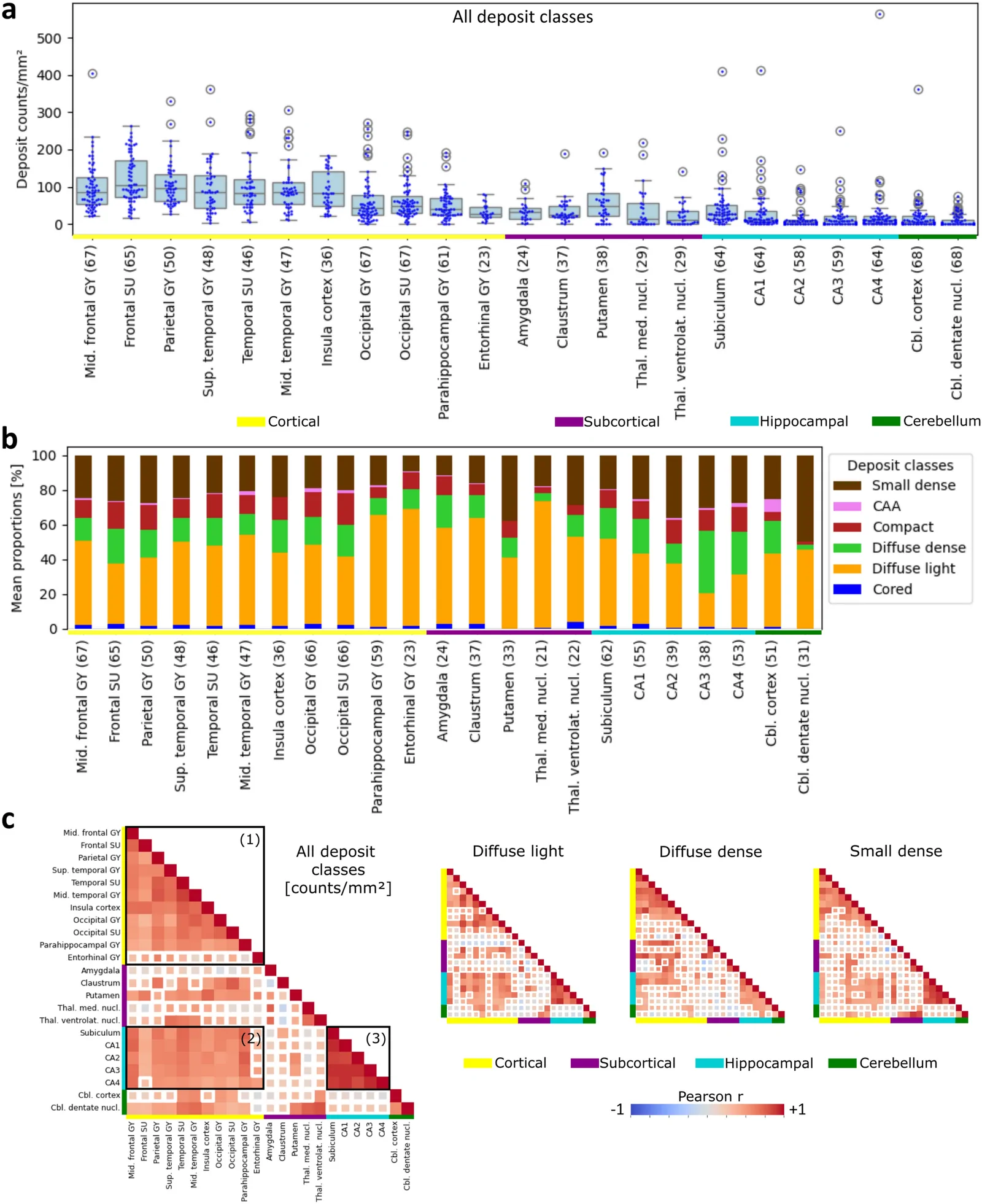
Aβ deposit classes across regions. **a** Deposit counts per mm^2^ summing up all deposit classes per region. **b** Mean proportions of deposit classes [%] in each region. In a and b, the numbers in brackets behind region names indicate how many cases are included in the above plot. Numbers in plot b can be lower as proportions of plaques cannot be calculated in images with zero detected deposits. **c** The heatmaps show Pearson correlation coefficients between brain regions regarding the total plaque count per mm^2^ (large plot) and the counts of diffuse light, diffuse dense and small dense plaques per mm^2^ (small plots). Non-significant correlations are labeled with a white frame. Of particular note are: (1) Positive correlations of Aβ plaque counts within the cortical brain regions. The exceptional role of the entorhinal cortex may be partly due to a limited number of cases for this region; (2) The hippocampal Aβ plaque counts correlate positively with the cortical plaque load; (3) The Aβ plaque counts are highly positively correlated within the hippocampal brain regions. CAA cerebral amyloid angiopathy, Cbl. cerebellum, GY gyrus, med. medial, mid. middle, nucl. nucleus, SU sulcus, Sup. superior, Thal. thalamus, vetrolat. Ventrolateral

While most cortical regions showed comparably high deposit counts (median between 81/mm^2^ in the insula cortex to 104/mm^2^ in the frontal sulcus), the occipital gyrus and sulcus, parahippocampal, and entorhinal cortex showed significantly lower deposit counts (median range 28/mm^2^ in the entorhinal cortex to 50/mm^2^ in the occipital sulcus) (Fig. 3a, S6, Table S2). Comparing the regions with a Kruskal–Wallis test and Dunn’s post hoc analysis with FDR correction for multiple testing (Table S4), the cortical regions predominantly showed significantly higher total Aβ deposit counts than the subcortical, hippocampal, and cerebellar regions (median range 0/mm^2^ in the dentate nucleus of the cerebellum to 49/mm^2^ in the putamen). The overall distribution persisted when comparing a subset of 40 cases with nine complete sampling areas with a Kruskal–Wallis test and Dunn’s post hoc analysis or Wilcoxon signed-rank test and subsequent FDR correction (Fig. S5, Table S11), limiting a potential sampling bias. Additionally, total and class-wise plaque densities between the complete subset (38 cases due to missing metadata in two cases) and the whole cohort were compared with multiple linear regression, correcting for age at death, sex and ApoE in the same nine brain regions. There was no significant difference after FDR correction, supporting the representativeness of the cohort. These results suggest the highest Aβ deposition in cortical regions with relatively homogeneous loads, with lower values only in the occipital or entorhinal regions. Subcortical, hippocampal, and cerebellar regions were less affected, suggesting fewer stressors, mechanisms sparing these regions, or more protective factors for Aβ deposition in these areas.

In a second step, the proportional load of each deposit class in relation to the total count of Aβ deposits in a specific region was compared between regions (Fig. 3b, Table S3). Diffuse light plaques predominated in most of the regions (median range from 12% in the CA3 region of the hippocampus to 78% in the medial nucleus of the thalamus). Small dense plaques ranked second and diffuse dense plaques ranked third in most of the regions. Compact plaques followed in fourth place. In general, the proportions of cored plaques and CAA played a subordinate role across regions with median percentages around 0%. These results suggest that a large proportion of the plaques correspond to poorly differentiated diffuse and often small deposits. In comparison, relatively few deposits belong to specific groups, such as CAA, compact or cored plaques. Across brain regions, cortical regions tended to have higher proportions of cored and compact plaques compared to non-cortical brain regions (Table S5, S8). Diffuse light plaques were especially dominant in the parahippocampal and entorhinal cortex and in the medial thalamic nucleus, while their proportion was relatively low in the CA3 and CA4 region of the hippocampus (Table S6). Frontal and occipital sulcal cortex showed increased proportions of CAA (Table S9). The entorhinal cortex presented with a relatively low proportion of small dense plaques (Table S10). The results largely agreed with the outcomes of a complete data subset in nine brain regions with predominantly similar results for the Kruskal–Wallis test with Dunn’s post hoc analysis and the Wilcoxon signed-rank test (Table S12–17). Comparing proportional deposit densities between the whole cohort and a complete subset (consisting of 14 cases due to incomplete metadata or no detected deposits in one or more of the nine brain regions) using multiple linear regression, there were no significant differences before or after FDR correction, also limiting a potential bias from sampling.

To evaluate if Aβ loads are correlated between brain regions, a Pearson correlation analysis was applied between the total plaque counts of 23 regions with subsequent FDR correction for multiple testing (Fig. 3c, S7, S8). Aβ deposit counts of all cortical regions were positively correlated except for the entorhinal cortex, partly due to fewer available stains in this region (Fig. S8). High positive correlations were also observed within the hippocampal regions and between the hippocampal and cortical regions. There were no significant negative correlations observed between Aβ loads of brain regions. Correlating the Aβ loads of specific deposit classes between brain regions (Fig. 3c, S9), the three positively associated region groups, namely cortical regions, hippocampal regions, and their combination, were also apparent for diffuse light, diffuse dense, and small dense plaques. There were rare significant positive correlations between brain regions regarding the densities of CAA, compact, or cored plaques.

### Correlations between Aβ deposits

**Fig. 4:**
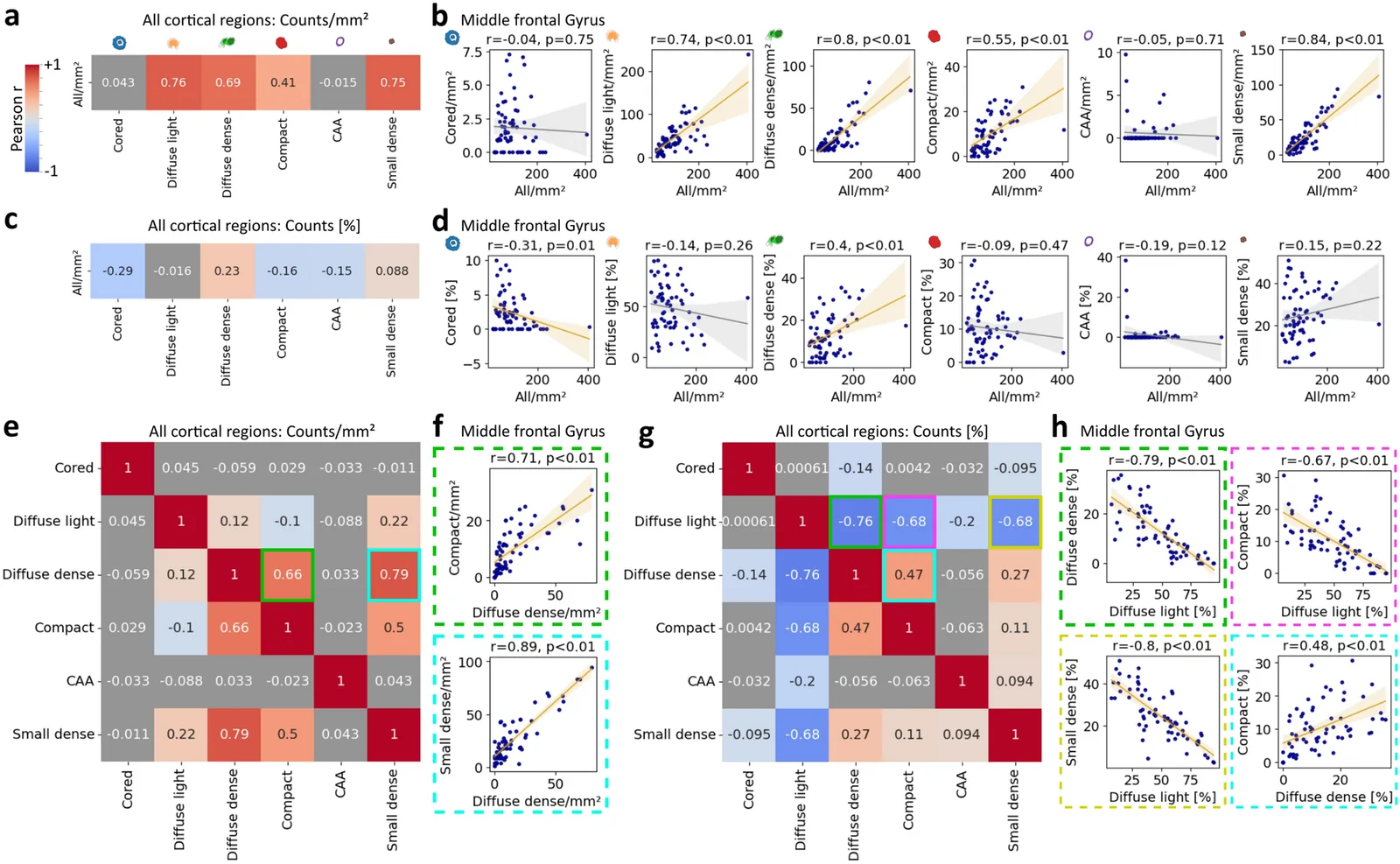
Associations between deposit classes. Non-significant correlations are grayed out in heatmaps and scatter plots. **a** Pearson correlation coefficients r across cortical regions between the total count of deposits (All/mm^2^) and counts/mm^2^ of individual deposit classes. Statistics were corrected for individual region names by linear regression; the significance level was set to <0.05 after false discovery rate correction. **b** Scatterplots illustrate the correlation analysis in parallel to (a) using the middle frontal gyrus as an example region. See Fig. S10 and S11 for results of other cortical regions. **c** Pearson correlation coefficients r across cortical regions between the total count of deposits (All/mm^2^) and the proportion of individual deposit classes in percent. Statistics in parallel to (a). **d** Scatterplots illustrate the correlation analysis in parallel to (c) using the middle frontal gyrus as an example region. **e** Heatmap showing correlations between plaque classes (counts/mm^2^) across cortical regions. Statistics in parallel to (a). **f** Scatterplots illustrate the two highest correlations of (e) in the middle frontal gyrus as an example region. (g) Heatmap showing correlations between plaque class proportions [%] across cortical regions. Statistics in parallel to (a). **h** Scatterplots illustrate the four strongest correlations of (g) in the middle frontal gyrus as an example region. Related false discovery rate corrected p-values from the heatmaps in this Fig. are presented in Fig. S12. Similar heatmaps with additional statistical correction for patient ID are presented in Fig. S13. Subfigures a-d with Aβ covered area instead of deposit densities (All/mm^2^) are shown in Fig. S14. CAA cerebral amyloid angiopathy

Next, the counts of individual deposit classes per region were correlated with the absolute counts of all deposits in the same region by Pearson correlation. This was done across cortical regions, after correcting for the region names by regression, and with subsequent FDR correction (Fig. 4a, S12), and for the middle frontal gyrus (MFG) separately for visualization purposes (Fig. 4b). Across cortical regions and in the middle frontal gyrus, there was a high positive correlation between the total absolute plaque count and the absolute counts of diffuse light (across cortex: *r*=0.76, *p*<0.001; MFG: *r*=0.74, *p*<0.01) and small dense plaques (across cortex: *r*=0.75, *p*<0.001; MFG: *r*=0.84, *p*<0.01), a moderate correlation with diffuse dense plaques (across cortex: *r*=0.69, *p*<0.001; MFG: *r*=0.8, *p*<0.01) and a low correlation with compact plaques (across cortex: *r*=0.41, *p*<0.001; MFG: *r*=0.55, *p*<0.01). In contrast, the densities of cored plaques and CAA did not correlate with the total Aβ plaque load. The latter results suggest a special role for cored plaques and CAA as not simply linearly accumulating.

To examine whether cases with higher total deposit counts show different class compositions, the above analyses were repeated with relative instead of absolute plaque counts (Fig. 4c and d). While the proportions of diffuse dense (across cortex: *r*=0.23, *p*<0.001; MFG: *r*=0.4, *p*<0.01) and small dense plaques (across cortex: *r*=0.088, *p*=0.043) increased weakly across cortical regions with increasing total plaque counts, the proportions of cored (across cortex: *r*=− 0.29, *p*<0.001; MFG: *r*=−0.31, *p*=0.01) and compact plaques (across cortex: *r*=−0.16, *p*<0.001), as well as CAA (across cortex: *r*=−0.15, *p*=0.001), were weakly negatively correlated. Keeping in mind that all included cases show advanced AD-related pathology and dementia, these findings indicate that diffuse dense plaques can broadly accumulate before death, while densities of cored plaques, compact plaques, and CAA do not increase to the same extent.

As an alternative measure for overall deposit loads, we correlated absolute and proportional loads of each deposit class with the Aβ covered area in the same cortical region (Fig. S14). The results largely correspond to the findings described above with deposit counts/mm^2^. A particularly strong correlation between diffuse dense plaques and Aβ covered area is noteworthy (across cortex: *r*=0.78, *p*<0.001; MFG: *r*=0.86, *p*<0.01), suggesting that particularly the number of diffuse dense plaques increases in severely Aβ-affected AD cases.

Furthermore, correlations between deposit class densities across cortical regions were examined with Pearson correlation and FDR correction, after adjusting for individual regions through linear regression. The overall heatmap and exemplary scatter plots from the MFG are shown in Fig. 4e and f. Of particular note are moderate to high positive correlations between diffuse dense and small dense plaques (across cortex: *r*=0.79, *p*<0.001; MFG: *r*=0.89, *p*<0.01), and diffuse dense and compact plaques (across cortex: *r*=0.66, *p*<0.001; MFG: *r*=0.71, *p*<0.01), suggesting that they not only show overlaps in terms of image morphology, but may also arise under the same conditions. Diffuse light plaques present minor positive correlations with small dense (across cortex: *r*=0.22, *p*<0.001) and diffuse dense plaques (across cortex: *r*=0.12, *p*=0.012). A weak negative correlation was observed between diffuse light and compact plaques (across cortex: *r*=−0.1, *p*=0.037), suggesting that cases tend to lean towards one or the other direction.

To investigate the associations between deposit classes, corrected for the total deposit counts, the correlation analysis was repeated with relative plaque counts (Fig. 4g and h). As before, there was a positive, but weaker correlation between diffuse dense and compact plaques (across cortex: *r*=0.47, *p*<0.001; MFG: *r*=0.48, *p*<0.01) and diffuse dense and small dense plaques (across cortex: *r*=0.27, *p*<0.001). This analysis further highlights a negative correlation between diffuse light plaques and diffuse dense (across cortex: *r*=− 0.76, *p*<0.001; MFG: *r*=−0.79, *p*<0.01), compact (across cortex: *r*=− 0.68, *p*<0.001; MFG: *r*=− 0.67, *p*<0.01), small dense plaques (across cortex: *r*=−0.68, *p*<0.001; MFG: *r*=−0.8, *p*<0.01), and CAA (across cortex: *r*=−0.2, *p*<0.001). These findings suggest separate mechanisms that lead to either diffuse light Aβ deposition in the cortex or formation of denser and more compact deposits, however, independently of cored plaques. When patient ID was included in the above analyses by linear regression, the Pearson correlation coefficients appeared slightly lower; however, overall, the results remained comparable (Fig. S13).

### Associations between Aβ deposits, tau and co-pathologies

**Fig. 5:**
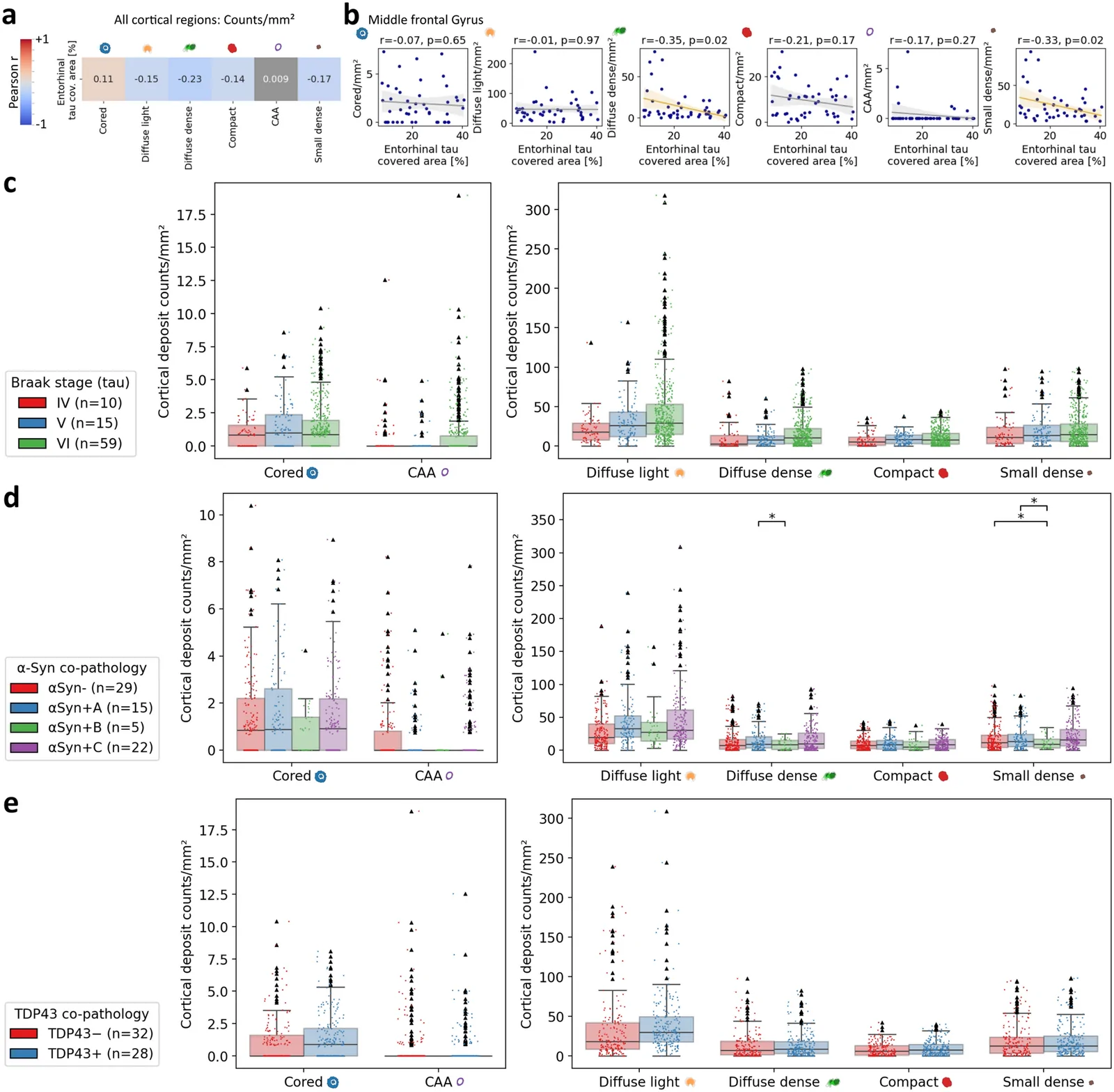
Associations between Aβ deposit classes and other protein deposits. **a** Pearson correlation coefficients r across cortical regions between the tau covered area in the entorhinal cortex (%) and counts/mm^2^ of individual deposit classes. Statistics were corrected for individual region names by linear regression; the significance level was set to <0.05 after false discovery rate correction. Non-significant correlations are grayed out. **b** Scatterplots illustrate the Pearson correlation analysis in parallel to (a) with tau covered area in the entorhinal cortex on the x-axis and Aβ deposit densities in the middle frontal gyrus on the y-axis. **c** Comparison of cortical Aβ deposit class densities between cases with different Braak and Braak (tau) stages. **d** Comparison of cortical Aβ deposit class densities between αSyn co-pathology groups. **e** Comparison between TDP43 co-pathology groups. In (c), (d) and (e), groups were compared with multiple linear regression correcting for ApoE genotype, age at death, sex, region names, and false discovery rate correction for multiple testing. The numbers (n =) indicate how many cases were included in the scatter dots. In the linear regression analyses, individual cases with incomplete data for the covariates were excluded. The parallel analysis with proportional Aβ deposit class loads is shown in Fig. S15. αSyn- no α-syn co-pathology, αSyn+A amygdala predominant αSyn co-pathology, αSyn+B brainstem predominant αSyn co-pathology, αSyn+C cortical disseminated αSyn co-pathology, *CAA* cerebral amyloid angiopathy, *cov.* covered

To examine whether specific Aβ plaques are correlated with the extent of an overall tau load measurement, the associations between absolute counts/mm^2^ of each Aβ deposit class with the tau-covered area in percent in the entorhinal cortex were examined across cortical regions (Fig. 5a, Fig. S15). After adjusting the Aβ values for individual regions using linear regression, the Aβ class counts and tau area ratio were examined for correlation using Pearson correlation and FDR correction. Only cored plaques showed a significant positive correlation with entorhinal tau (*r*=0.11, *p*=0.034). The entorhinal tau load was significantly negatively correlated with diffuse light (*r*=−0.15, *p*=0.006), diffuse dense (*r*=−0.23, *p*<0.001), compact (*r*=−0.14, *p*=0.01), and small dense plaques (*r*=−0.17, *p*=0.002) across cortical regions. No correlation between CAA/mm^2^ and the tau-covered area in the entorhinal cortex was observed. Additionally correcting for patient ID with linear regression led to no significant results (Fig. S13). Correlating percental loads of Aβ plaques across cortical regions with the entorhinal tau load, there was a significant positive correlation with cored plaques (*r*=0.14, *p*=0.027) and a significant negative correlation with diffuse dense plaques (*r*=− 0.12, *p*=0.049). In total, these results suggest that cored plaques are more likely to be associated with a higher entorhinal tau load, while other plaque classes accumulate more with lower tau loads.

To check whether Aβ and tau are correlated within the same cortical region, Aβ deposit densities were Pearson correlated with the tau covered area of the same region (Fig. S16 and S17). With few exceptions, including the occipital cortex showing weak positive correlations for several Aβ deposit classes, the Aβ classes and local tau load were mostly not correlated. This finding suggests there may hardly be a direct, local connection between tau and Aβ.

To examine the association between Aβ deposit densities and Braak and Braak stages as a tau measure, we applied pairwise multiple linear regression models, correcting for ApoE genotype, age, sex and region names (Fig. 5c, Fig. S15). Plaque loads were comparable between Braak and Braak stages IV to VI with no significant differences after FDR correction. Before FDR correction, there was a trend towards increased densities of cored plaques in the Braak and Braak stage V group compared to IV (*β*=0.87, p_without FDR_=0.008, p_FDR_=0.05) and VI (*β*=−0.46, p_without FDR_=0.029, p_FDR_=0.11). Additionally, there was a trend towards increased loads of diffuse dense plaques in Braak and Braak stage VI (compared to IV: *β*=2.86, p_without FDR_=0.015, p_FDR_=0.07; compared to V: *β*=4.87, p_without FDR_=0.008, p_FDR_=0.05) and increased CAA densities in Braak stage VI compared to stage V (*β*=0.51, p_without FDR_=0.006, p_FDR_=0.05). Regarding proportional plaque loads, Braak and Braak stage VI was associated with increased proportions of diffuse dense plaques (compared to stage IV: *β*=2.3, p_FDR_=0.03) and decreased proportions of small dense plaques (compared to stage IV: *β*=−2.4, p_FDR_=0.03). These findings suggest a trend towards slightly higher Aβ deposit loads and a tendency towards higher proportions of diffuse dense plaques with higher Braak and Braak stages, while the cortical density of diffuse dense plaques was negatively correlated with the entorhinal tau load. Thus, high Braak and Braak stages should not be equated with high entorhinal tau load, pointing to a nuanced picture.

To test whether α-syn co-pathology distribution groups, namely α-syn negative, amygdala predominant, brainstem predominant, and disseminated cortical α-syn pathology vary in their cortical Aβ class loads, pairwise multiple linear regression models were applied, correcting for ApoE genotype, age, sex and region names (Fig. 5c, Fig. S15). Plaque loads were mostly comparable across all groups. After FDR correction for multiple testing, three group differences stayed significant: the density of diffuse dense (*β*=−9.0, *p*=0.017) and small dense plaques (*β*=−11.7, *p*=0.011) was smaller in cases with brainstem predominant α-syn co-pathology than in cases with amygdala predominance; the latter finding was also true for α-syn negative cases showing higher amounts of small dense plaques than the brainstem predominant α-syn positive group (*β*=−5.1, *p*=0.017). These findings suggest a deviation of dense Aβ deposition in the α-syn brainstem group. Regarding percentage loads, the proportion of small dense plaques was significantly smaller in cases with amygdala predominant α-syn than in α-syn negative cases (*β*=−4.9, *p*=0.042) or cortical α-syn positive cases (*β*=2.25, *p*=0.042). In general, we did not find a strong association between α-syn co-pathology and different Aβ plaque classes.

We compared AD cases with and without TDP43 co-pathology with multiple linear regression, correcting for region names, ApoE genotype, sex and age at death (Fig. 5e, Fig. S15). TDP43 deposition was examined in the hippocampus, amygdala and medulla oblongata where available. There were lower densities of CAA and small dense plaques in TDP43-positive cases, which stayed not significant after FDR correction. Comparing Aβ class proportions, there were increased rates of diffuse light plaques in TDP43-positive cases (*β*=11.0, *p*=0.001) and decreased rates of small dense plaques (*β*=−2.6, *p*=0.001) and CAA (*β*=−6.1, *p*<0.001). These findings suggest an association between diffuse light Aβ plaques and TDP43 deposits, possibly due to overlaps in impaired protein degradation and clearance pathways. Small dense plaques and CAA occurred proportionally more frequently in cases without TDP43 deposition.

### Associations between Aβ deposits, sex, and genotypes

**Fig. 6:**
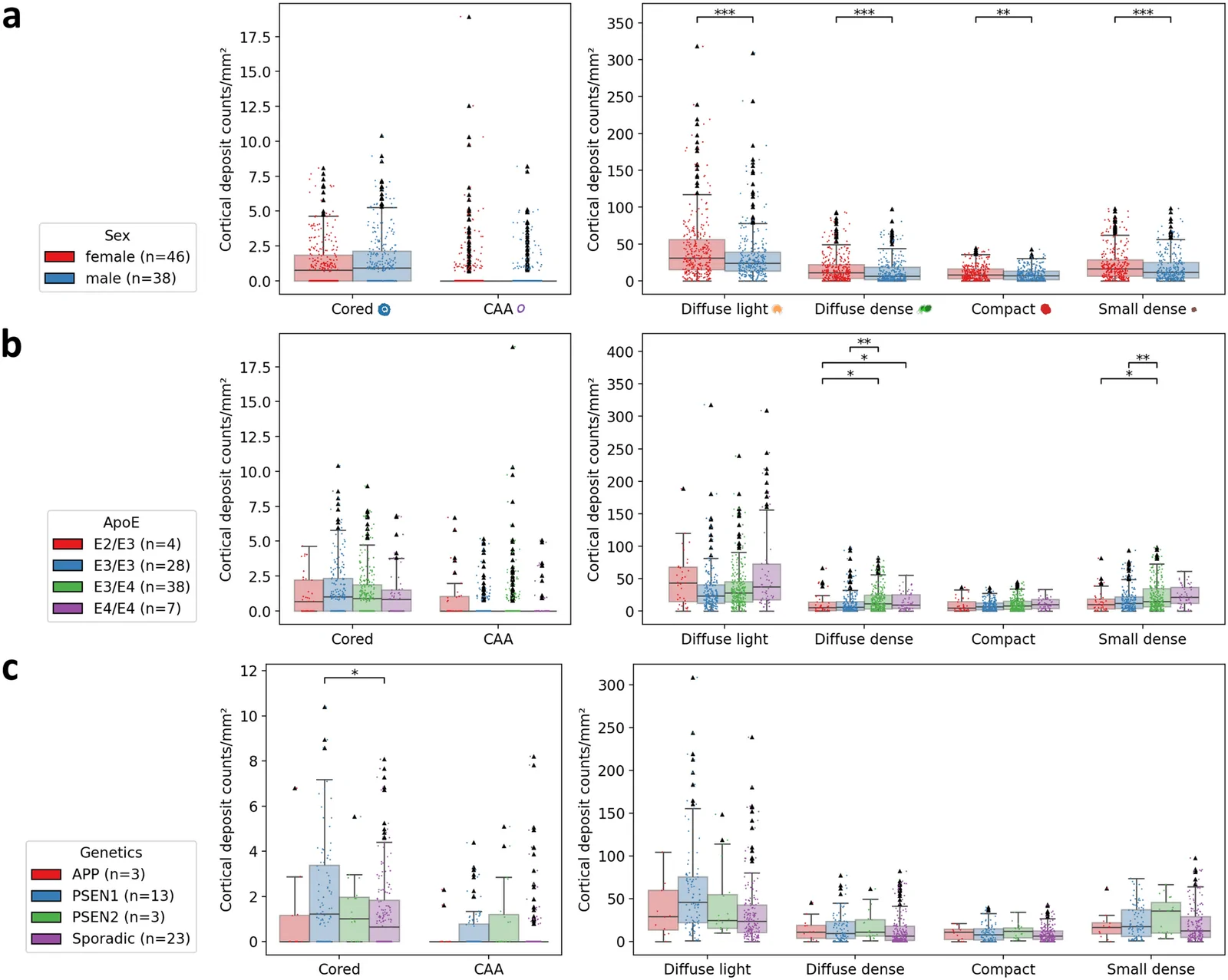
Associations between Aβ deposit classes and genetic factors. **a** Comparison of cortical Aβ deposit class densities between female and male Alzheimer’s disease (AD) cases. Groups were compared with multiple linear regression correcting for ApoE genotype, age at death, and region names. **b** Comparison between ApoE genotypes. Groups were compared with multiple linear regression correcting for sex, age at death, and region names. **c** Comparison between familial and sporadic Alzheimer’s disease cases. Groups were compared with multiple linear regression correcting for sex, ApoE genotype, and region names. Sporadic cases were defined as AD with a reported age of onset >65 years and an inconspicuous whole genome sequencing for AD-related variants. In (a), (b) and (c), p-values were corrected for multiple testing by respective false discovery rate correction. The numbers (n =) indicate how many cases were included in the scatter dots. In the linear regression analyses, individual cases with incomplete data for the covariates were excluded. The parallel analysis with proportional Aβ deposit class loads is shown in Fig. S18. CAA cerebral amyloid angiopathy

Correcting for age at death, ApoE genotype, and region names (Fig. 6a, Fig. S18) in multiple linear regression models, female cases presented with significantly higher absolute densities of diffuse light (*β*=−17.3, *p*<0.001), diffuse dense (*β*=−6.1, *p*<0.001), compact (*β*=−2.2, *p*=0.007), and small dense deposits (*β*=−6.1, *p*<0.001) but, surprisingly, equal counts of cored plaques and CAA. Since the differing absolute values shift the ratios, males showed significantly higher proportions of cored plaques (*β*=0.67, *p*=0.046) and lower proportions of diffuse dense plaques (*β*=−3.0, *p*=0.024). These findings indicate that females generally accumulate higher Aβ plaque loads, except for cored plaques, which do not show a sex tendency in this cohort of advanced AD cases.

To investigate the effect of the ApoE genotype on cortical Aβ deposit classes, the absolute and relative amounts of each deposit class were compared between ApoE genotypes (E2/E3, E3/E3, E3/E4, E4/E4) with pairwise multiple linear regression, correcting for age at death, sex, and region names (Fig. 6b, Fig. S18). There were significantly higher densities of diffuse dense and small dense plaques in cases with at least one E4 allele in comparison with cases without E4. The same trend, although not significant, was observed for compact and partly for diffuse light plaques. No difference was observed for cored plaques, statistically limited by a low density and methodological constraints. There was a non-significant trend of more CAA in cases with an E2 allele (E2/E3 vs. E3/E3: *β*=−0.40, p_without FDR_=0.06, p_FDR_=0.16). Comparing proportions of deposit counts across ApoE genotypes, cases with E2/E3 constellation showed higher proportions of diffuse light deposits (*β*=−8.3, *p*=0.018) and smaller proportions of small dense deposits (*β*=4.0, *p*=0.018) than AD cases with E3/E4 genotype. These results suggest that the ApoE genotype partly affects the Aβ plaque composition, towards more dense deposits in cases with an E4 allele.

To examine the effect of specific AD-related mutations on cortical Aβ plaque classes, cases with APP, PSEN1 or PSEN2 mutation as well as sporadic case were compared pairwise with multiple linear regression, correcting for ApoE genotype, sex and region names (Fig. 6c, Fig. S18). In this analysis, *sporadic* cases were defined as AD with a reported age of onset >65 years and an inconspicuous whole-genome sequencing for AD-related variants. After FDR correction for multiple testing, there remained only one significant difference with PSEN1 mutation carriers having absolutely more cored plaques than sporadic AD cases (*β*=−0.48, *p*=0.019). Without FDR correction, cases with PSEN2 mutation showed slightly higher diffuse light deposit counts than sporadic cases (*β*=−24.8, p_without FDR_=0.005, p_FDR_=0.10), more diffuse dense plaques than PSEN1 mutation carriers (*β*=7.8, p_without FDR_=0.046, p_FDR_=0.25), more compact plaques than PSEN1 mutation carriers (*β*=4.28, p_without FDR_=0.045, p_FDR_=0.25), and more small dense plaques than PSEN1 mutation carriers (*β*=10.7, p_without FDR_=0.031, p_FDR_=0.25), however, with a limited number of only three PSEN2 carriers. In summary, the findings suggest that a PSEN1 mutation is associated with more cored Aβ plaques. Conclusions regarding PSEN2 are not reasonable due to the small number of cases.

### Associations between Aβ deposits, age at disease onset, disease duration and age at death

**Fig. 7:**
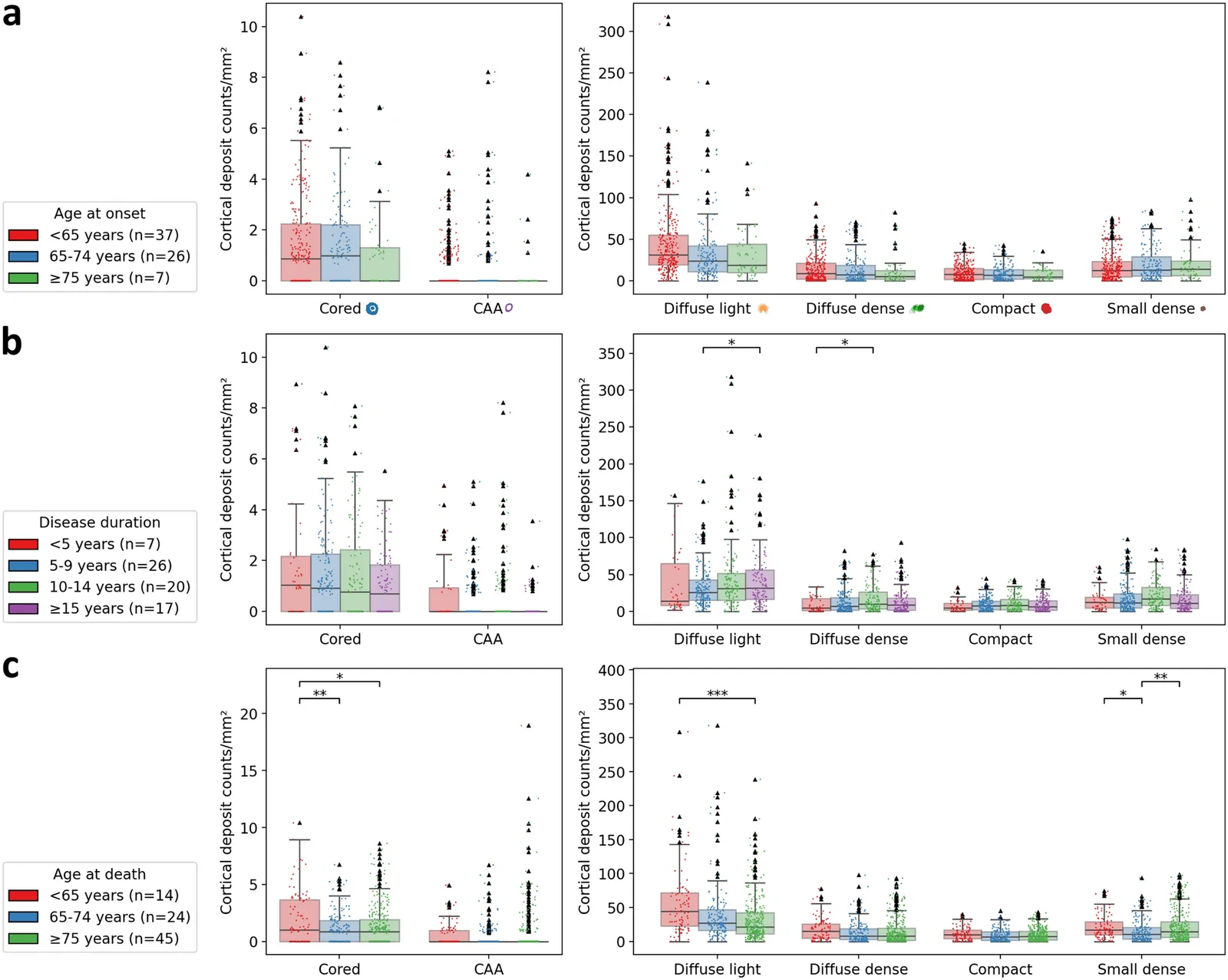
Associations between Aβ deposit classes and age. **a** Comparison of cortical Aβ deposit class densities between early, middle-aged and late onset Alzheimer’s disease (AD) cases. Groups were compared with multiple linear regression correcting for ApoE genotype, sex, and region names. **b** Comparison between different disease duration groups. Groups were compared with multiple linear regression correcting for sex, ApoE genotype, age at death, and region names. **c** Comparison between three groups with varying age at death. Groups were compared with multiple linear regression correcting for sex, ApoE genotype, and region names. In (a), (b) and (c), p-values were corrected for multiple testing by respective false discovery rate correction. The numbers (n =) indicate how many cases were included in the scatter dots. In the linear regression analyses, individual cases with incomplete data for the covariates were excluded. The parallel analysis with proportional Aβ deposit class loads is shown in Fig. S19. CAA cerebral amyloid angiopathy

Comparing the plaque densities between three groups of age at clinical onset (< 65 years, 65–74 years and ≥75 years) (Fig. 7a, Fig. S19) with multiple linear regression, correcting for ApoE genotype, sex and region name, the oldest group with onset ≥75 years tended to have fewer cored plaques than the middle group (65–74 years) (*β*=−0.68, p_without FDR_=0.014, p_FDR_=0.25) and fewer diffuse dense plaques than the youngest group (< 65 years) (*β*=−2.46, p_without FDR_=0.032, p_FDR_=0.25). Regarding proportional Aβ deposit loads, the youngest group (< 65 years) showed significantly higher loads of diffuse light plaques than the middle-aged group (65–74 years) (*β*=−7.4, *p*=0.013) and fewer small dense plaques than the middle aged (*β*=5.9, *p*<0.001) and the oldest group (≥ 75 years) (*β*=3.3, *p*<0.001). These findings suggest that cored plaques and a higher amount of diffuse light plaques are associated with younger age at onset, keeping in mind that familial AD cases are included in the cohort.

To estimate the association between disease duration and cortical Aβ deposition, the absolute and relative amounts of each deposit class were compared between four groups (< 5 years, 5–9 years, 10–14 years, ≥15 years). The multiple linear regression models were corrected for age at death, sex, ApoE genotype, and region names (Fig. 7b, Fig. S19). A longer disease duration (≥ 15 years) was associated with higher absolute counts of diffuse light plaques than a duration of 5–9 years (*β*=6.4, *p*=0.040). A longer duration of 10–14 years was associated with higher diffuse dense plaque counts than <5 years (β=5.2, *p*=0.026). The results suggest an accumulation of diffuse plaques over time with dementia. Cored plaques, CAA, Compact and small dense plaques were comparable between disease duration groups. Comparing the class proportions, a short disease duration (< 5 years) was significantly associated with a higher proportion of cored plaques than in cases with long disease duration (≥ 15 years) (*β*=−0.57, *p*=0.016) and CAA compared to 5–9 years disease duration (*β*=−1.21, *p*=0.039) and ≥15 years disease duration (*β*=−0.62, *p*=0.016). While the proportions of diffuse light and diffuse dense deposits were mainly increasing with longer disease durations, the proportions of compact and small dense plaques tended to decrease with longer disease durations. Although the estimation of disease duration is partly subjective, the findings suggest that diffuse light and diffuse dense plaques can accumulate over time to a considerable extent without having an immediately life-limiting effect. In contrast, cases with short disease durations show equal amounts of cored plaques and CAA, which, therefore, might be crucial for clinical disease progression.

To assess the relationship between age at death and Aβ plaque loads, the absolute and relative amounts of each deposit class were compared between three age groups (< 65 years, 65–74 years, ≥75 years) with multiple linear regression across cortical regions, correcting for sex, ApoE genotype, and region names (Fig. 7c, Fig. S19). It is noticeable that a young age at death (< 65 years) was significantly associated with higher absolute amounts of cored plaques compared to the middle-aged group 65–74 years (*β*=−0.88, *p*=0.0025) and to the aged group ≥75 years (*β*=−0.34, *p*=0.019). Furthermore, <65 years patients had higher loads of diffuse light deposits than ≥75 years patients (*β*=− 11.6, *p*<0.001). Regarding small dense plaques, the middle group (65–74 years) showed lower densities than the younger group (< 65 years) (*β*=−5.4, *p*=0.043) and the older group (≥ 75 years) (*β*=6.0, *p*=0.005). On a proportional level, the oldest group (≥ 75 years) showed a significantly lower proportion of diffuse light plaques than 65–74 years old patients (*β*=−7.0, *p*=0.027) and increased rates of small dense plaques compared to <65 years (*β*=2.7, *p*=0.003) and 65–74 years groups (*β*=6.8, *p*<0.001). Together, these findings indicate that patients dying younger bear higher loads of cored and diffuse light Aβ plaques before death, which might be partly explained by familial AD cases in the cohort.

### Integration of Aβ deposit determinants

**Fig. 8:**
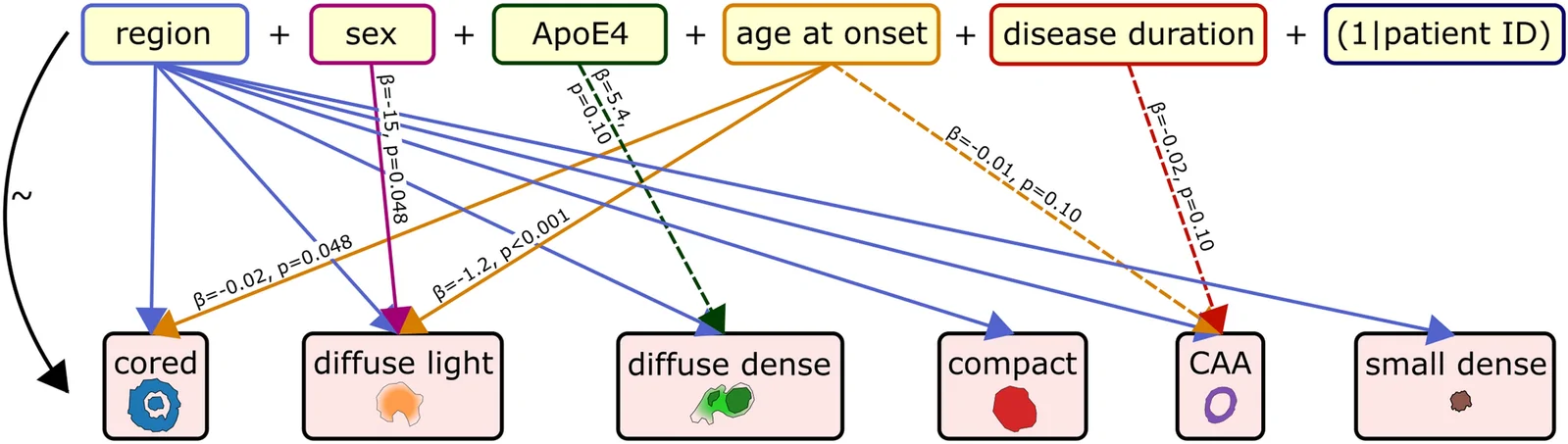
Integrative analysis of potential Aβ plaque composition determinants. To investigate the associations of various factors (upper line in yellow) with densities (counts/mm^2^) of the six deposit classes (lower line in red), a linear mixed-effect model was created for each deposit class separately. Only cortical brain regions were included. Region name, sex (female vs. male), ApoE4 (no E4 vs. at least one E4 allele), age at onset, and disease duration were independent factors, patient ID was added as a random effect (1|patient ID), and the deposit classes were the dependent variable, respectively. Significant effects after correction for false discovery rate (FDR) due to multiple testing are indicated with solid arrow lines, and associations that were only significant before FDR correction are labeled with dashed lines. Results for exemplary cortical brain regions are shown in Fig. S20. CAA cerebral amyloid angiopathy

Finally, sex, ApoE, age at onset and disease duration were integrated into linear mixed-effects models across cortical brain regions, correcting for the brain region and a random factor for individual subjects (Fig. 8). Excluding data points with incomplete metadata, 461 observations per deposit class from 64 patients were included in this analysis. There was a significant association between female sex and increased densities of diffuse light plaques (coefficient *β*=−15, *p*=0.048). An association between the ApoE4 allele and diffuse dense plaques was detected, but did not survive FDR correction (*β*=5.4, p_without FDR_=0.040, *p*=0.10). A lower age at onset, also indicating familial AD cases, was significantly associated with higher densities of cored plaques (*β*=−0.02, *p*=0.048), diffuse light plaques (*β*=−1.2, *p*<0.001), and CAA (*β*=−0.01, p_without FDR_=0.041, *p*=0.10), while the latter did not remain significant after FDR correction. A shorter disease duration was associated with higher CAA densities, which also did not survive FDR correction (*β*=−0.02, p_without FDR_=0.037, *p*=0.10).

Repeating this analysis with multiple linear regression models in individual brain regions, e.g., middle frontal gyrus, occipital gyrus and sulcus, the results were partially replicated, while other correlations differed (Fig. S20). An association repeatedly emerged between younger age at onset and higher densities of cored and diffuse light plaques, as well as between female sex and increased diffuse light plaque loads, whereas a relationship between disease duration and CAA was not observed in the exemplary regions. According to R^2^ and adjusted R^2^ values, indicating how well the models predict the variance of the dependent variable, the factors sex, ApoE, age at onset and disease duration hardly explain the densities of compact plaques or CAA (R^2^_adj_ ≤ 7%), weakly explain the density of cored plaques (R^2^_adj_ up to 13%), and partly explain the densities of diffuse light (R^2^_adj_ up to 37%), diffuse dense (R^2^_adj_ up to 19%) and small dense plaques (R^2^_adj_ up to 36%) with the highest scores in the occipital sulcus.

## Discussion

Detecting Aβ deposits in 84 cases of Alzheimer’s disease (AD), we successfully distinguished six different deposit classes with a convolutional neural network. A high and intercorrelated Aβ load was observed in cortical regions compared to lower loads in subcortical areas. Diffuse light and small dense plaques dominated in terms of numbers. The relationships between deposits, associated factors and determinants are complex and not uniform across the different types of deposits and brain regions.

### Aβ deposit classification

Based on manual classification of 3139 Aβ deposits, a convolutional neural network was trained, optimized and tested to distinguish six deposit classes, reaching an overall recall of 81.5% and a precision of 82.4% in the test set. Subsequent visual control of cored plaques and CAA prediction ensured no false positives in these two groups which have a tendency towards reduced precision due to the low count compared to the other classes. Distinct cotton-wool plaques [45] and coarse-grained plaques [9, 10] were too rare for a stable prediction and were pooled in the compact plaque class.

Our convolutional neural network architecture was an extension of the model by Tang et al. [71], which classifies Aβ deposits into diffuse, cored plaques and CAA with a very high accuracy/recall of 0.987. It should be noted that the test set in Tang et al. [71] was unbalanced towards diffuse plaques and the model can, therefore, achieve a very high general accuracy. Besides the addition of a convolutional layer and larger, grayscale images as input, the main improvement is that our newly trained model distinguishes more classes and can thereby better reflect reality, capturing numerous deposits that do not explicitly fall into cored or diffuse plaques and also smaller deposits that were more generously excluded in Tang et al. [71]. Another model, e.g. the model by Wong et al. [89], shows a comparable performance to our model, but is limited to cored plaques and CAA. The model by Amin et al. [3] performed highly, but is also restricted to three deposit classes and not realistic for our study approach. Thus, the performance of our model is lower or comparable to models in the literature, but improved by partial visual controls and tailored to the specific dataset and research question, covering a wide range of Aβ deposits and thus being closer to reality.

### Aβ deposition across brain regions

Aβ plaques were quantified in advanced AD cases stained with the antibody clone 4G8 across brain regions. The Aβ loads were highest in the frontal, parietal, temporal and insula cortex and lower in the occipital, parahippocampal and entorhinal cortex, subcortical, hippocampal and cerebellar regions. The findings align with the literature and follow the Thal stages, progressing from high amounts in the neocortex to the later and less affected allocortex and hippocampus and finally the cerebellum [73].

Interestingly, the regions that are commonly affected regarding intracellular tau, α-syn and TDP43 pathology, such as the amygdala, hippocampus, and entorhinal cortex, are less affected by Aβ. Projection neurons affected by tau pathology, being associated with Aβ deposition in the targeted cortical regions via Aβ release from terminal axon segments, might explain a part of this pathology distribution [13, 14]. The released Aβ peptides can diffuse into the extracellular space [13] and accumulate into oligomers and larger aggregates later on [38]. This theory is supported by the fact that there were hardly any correlations between tau and Aβ within the same brain region. A targeted correlation analysis regarding connected brain regions, especially the brainstem-cortical axis, is needed to pursue this theory further.

A focus on connections would also be interesting in terms of the positive correlations of the plaque loads within cortical regions, within hippocampal regions, and between cortical and hippocampal regions. However, connectivity alone does not explain the extent of Aβ accumulation; for instance, claustrum and thalamus are diffusely connected regions, but exhibit only medium plaque loads.

Across regions, diffuse plaques dominated the scene. Fleecy diffuse plaques are known to appear in early stages [74]. Although at low percentages, there are trends towards more compact and cored plaques in cortical regions. This could be due to a developmental course from diffuse to ‘mature’ plaques in more and earlier affected cortical regions or because of regional factors that increase the Aβ load and the probability for these more defined plaque types to occur [72]. The density of cored plaques did not correlate with the overall Aβ plaque density in the same region, which points to a separate mechanism of origin for cored plaques.

In the following paragraphs, we will firstly discuss the most outstanding findings for each Aβ plaque class, including individually associated factors. Afterwards, the probable determinants are discussed in a synopsis.

### Cored plaques

Cored plaques were defined as Aβ plaques with a dense core with a free ring and a denser rim. Compared to other deposits, their density was relatively low. The true number of cored plaques was probably underestimated as the core is sometimes not visible depending on the sectional plane. In previous studies, the observed density of cored plaques was described as higher, with around 20% of the Aβ plaques in advanced AD [25], but it is mostly in line with the counts of neuritic senile plaques in Delaère et al. [20]. In the manually annotated Aβ plaque set in Tang et al. [71], the number of cored plaques compared to dominating diffuse plaques was also low with 2.2% of annotated images, pointing in our direction. Furthermore, we included many small dense plaques in our analyses, which are commonly excluded in other studies, further reducing the proportion of cored plaques. In our study, the proportional load of cored plaques decreased in cases with generally higher Aβ loads, questioning the simplicity of a proposed linear development from diffuse to compact to cored plaques.

It is striking that the cored plaque density remained stable across most of the cohort of advanced AD cases, independently of sex and ApoE genotype. The effect of ApoE4 on cored plaques is inconsistent in the literature [7, 17, 59] and is also biased by a cohort of advanced AD cases in our study. There were higher densities of cored plaques in people with a younger age at death and patients with a PSEN1 mutation. Although not significant in every study [90], there is a trend towards higher Aβ loads, especially Aβ42, in familial and early onset AD cases in the literature [29, 42, 51].

Interestingly, cored plaques were the only plaque class showing a positive correlation with the entorhinal tau load. A large proportion of cored plaques are also neuritic plaques [22, 25], which are associated with activated microglia and reactive astrocytes [22]. Especially cored, neuritic plaques, tau and synaptic and neuron loss are correlated with cognitive decline [7, 23, 50, 64, 66]. In summary, cored plaques could be more clinically relevant than other plaque types despite the comparably low density.

### Diffuse light plaques

Diffuse light plaques, defined as mostly low-intensity Aβ, represent the largest group of plaques across most of the brain regions. They occur early in the disease [56], mainly consist of Aβ42 and usually lack fibrils [37, 92]. In our study, diffuse light plaques showed low positive correlations with diffuse dense and small dense plaques and were slightly negatively correlated with the density of compact plaques. Hypothetical explanations could be that, with enough diffuse light plaques, some large diffuse light plaques will develop to diffuse dense plaques by continuous Aβ production and some small diffuse light plaques might develop to small dense plaques [49, 82, 93], probably accompanied by a shift from Aβ42 to Aβ40 [54, 55]. Apart from that, mouse experiments suggested distinct mechanisms showing diffuse plaque accumulation does not unconditionally increase compact plaque load [48]. It is, therefore, also conceivable that diffuse light plaques resolve and denser and compact deposits form by different mechanisms, instead of a direct transition from one form to the other.

The density of diffuse light plaques was negatively correlated with the entorhinal tau load. This finding is in line with previous studies showing that diffuse Aβ plaques do not correlate with synapse loss or functional scores [50, 52] and were less or not associated with neuritic components or microglia [25, 36, 91].

### Diffuse dense plaques

With increasing deposit loads, the proportion of diffuse dense plaques, here defined as diffuse plaques with dense, irregular centers and indistinct edges, increased in cortical regions. Thereby, the density of diffuse dense plaques correlated positively with compact and small dense plaques and weakly with diffuse light plaques. There was a higher cortical density of diffuse dense plaques in female cases and in ApoE4 carriers.

Transitional Aβ plaque forms between specified plaques are hardly discussed in the literature, which makes it difficult to interpret these findings. However, a spectrum of fine diffuse and dense diffuse plaques as well as the appearance of amyloid fibrils in denser parts of some diffuse plaques were described before [49, 92]. In our analyses, two results stand out: Firstly, with increasing total Aβ plaque load, the proportional share of diffuse dense plaques is the only one that shows a discernible increase. Thus, either a high Aβ plaque density might lead to denser cores in diffuse plaques or confounders, causing generally high Aβ loads, also promote diffuse dense plaque accumulation. Secondly, ApoE4 was repetitively associated with higher densities of diffuse dense plaques in different statistical models, suggesting that ApoE4 contributes to the compactness of Aβ plaques. This result is in line with a study in mice showing more dense plaque morphologies in ApoE4 carriers [61]. The exact mechanisms of ApoE4 increasing the AD risk and the Aβ load are still under discussion; an enhanced aggregation and an adverse effect on the Aβ clearance are assumed [46].

### Compact plaques

Compact plaques, having a mostly sharp border and often oval or round shape, included subtypes, such as cotton-wool plaques and coarse-grained plaques. We refrained from differentiating these subclasses to avoid making the classes too small, and thereby further reducing the performance of the classifier. Despite the name *compact*, a porous structure is common due to constant aggregation and resolution in the plaque [18]. The density of compact plaques correlated positively with diffuse dense and small dense plaques, suggesting a relationship and possibly a transition between diffuse dense, small dense and compact plaques. On the other hand, there was a negative correlation with diffuse light plaques, indicating some conditions in AD cases might lead towards more compact plaques, such as CLAC (collagenous Alzheimer amyloid plaque component), suggested by Hashimoto et al. [33] or more diffuse plaques via different pathways [48].

### Cerebral amyloid angiopathy

Cerebral amyloid angiopathy (CAA) of the Aβ type is here defined as Aβ deposition in leptomeningeal or intraparenchymal blood vessel walls, mostly small arteries and less often capillaries. CAA was slightly pronounced in the frontal sulcus and especially in the occipital cortex, in line with the literature [77, 79].

We neither found a correlation between CAA and the overall Aβ plaque load across cortical regions nor between CAA and other Aβ plaque classes. Thus, a statistical correlation between CAA and dense Aβ depositions, as suggested by Kumar-Singh [41], was not proven in our dataset. We found a comparable CAA density in female and male patients and, thereby, were also unable to confirm a higher CAA load in male cases that has been described before [68]. These divergent results could be attributed to the relatively small annotated brain regions in our study, whereby low-density deposits are not always represented in a characterizable manner. Before correction for multiple testing, there were higher CAA densities in AD cases with earlier age at onset and shorter disease duration, in agreement with former findings [76].

In previous literature, ApoE4 was described as a risk factor for CAA, while ApoE2 was associated with CAA in larger arteries [68, 73]. Although we did not find a significant difference in our cohort regarding the ApoE status, there was a non-significant trend towards higher CAA density in ApoE2 carriers vs. E3 homozygous patients. Whether a higher density of CAA suggests an overloaded perivascular clearance [8, 41] and if it is independently associated with cognitive decline [11] cannot be further verified based on the data obtained.

### Small dense plaques

Small dense plaques were defined as small (but larger than 100 µm^2^), mostly compact Aβ accumulations. Similarly to diffuse dense plaques, this class is not explicitly defined in the literature, but was considered necessary for this analysis to accommodate the variability in the cases. A proportion of the detected small dense plaques probably corresponds to cores of cored plaques and burned-out plaques [41, 82] although there was no positive correlation between small dense plaques and cored plaques. Another portion likely corresponds to larger accumulations of intracellular deposits [19]. Some will be nonspecific, condensed extracellular deposits, which may have arisen from previously small, diffuse light or diffuse dense deposits or will develop towards diffuse dense or compact plaques. It is conceivable that some of these deposits are in the process of growing or disappearing [15, 70, 94], however, the proportions are unclear.

The density of small dense plaques correlated positively with the total Aβ plaque load and with the densities of diffuse dense, compact, and weaker with diffuse light plaques, suggesting developmental overlaps between these classes. Following previous observations [65], there was no trend from small dense plaques to larger (diffuse dense or compact plaques) across the spectrum of less to severely affected regions or across disease durations. The ApoE4 genotype was associated with more small dense plaques, in line with a higher condensation observed in E4 allele carriers as discussed for diffuse dense plaques [61].

### Integration of Aβ deposit determinants

Sex, ApoE, age at onset and disease duration were integrated in a statistical model to predict plaque densities in cortical regions. We found complex, often weak and partly region dependent relationships between these factors and individual Aβ plaque classes. Among the stronger effects were a positive correlation between female sex and a higher density of diffuse light plaques. Furthermore, younger age at clinical disease onset, partly due to familial AD cases, was associated with higher densities of cored and diffuse light plaques. Although not significant after FDR correction, there was a trend towards more diffuse dense and small dense plaques in ApoE4 carriers.

The results suggest that risk and clinical factors are not uniformly associated with all forms of Aβ deposits, but rather correlate with specific deposit classes in specific regions, e.g., ApoE4 with denser deposits and young age at onset with cored plaques. The exact results vary depending on the co-factors in statistical models, which points to often weak effects and interdependencies between factors. Interactions between different factors were not explicitly addressed in our simplified model. However, in the literature, there are indications that age, sex and ApoE status have different effects in different combinations [28, 84] and confound the disease duration [75]. These factors were also associated with different ratios of soluble and fibrillar Aβ in non-demented people at varying degrees across brain regions [16].

In summary, relationships appear complex and result in distinct compositions within individual brains. This study may serve as groundwork for cross-regional imaging and molecular analyses by providing a metadata-differentiated dataset for further correlative analyses (Table S18, S19). However, a significant portion of the plaque density variability remains unexplained by the factors investigated in this study, suggesting that further determinants, such as lifestyle elements and co-morbidities, may also play a role.

### Limitations and further questions

This study is an approach to investigate determinants and associated factors of Aβ deposition in a large AD cohort across brain regions. Numerous Aβ deposits were automatically detected and classified into six deposit classes. Observations were correlated with co-pathology, genetic and clinical data, providing a comprehensive overview.

An important limitation of this project is the bias in the dataset, based on voluntary brain donation in a Western country. This leads to a missing ethnic diversity and an overrepresentation of familial AD cases. The effect of AD-related mutations on the Aβ plaque composition was analyzed separately to estimate the impact. Second, there is an unbalanced sampling of areas between subjects which might introduce a bias in the downstream analyses. Control analyses in a complete data subset excluded big distortional effects, but a bias in individual analyses is still conceivable. Third, the annotated rectangles in each region are relatively small, bearing the risk that these are not representative of the whole region. This applies in particular to deposits with low densities, i.e., CAA and partly cored plaques, or regions with low Aβ loads. Statements regarding CAA density are, therefore, made with caution in this analysis. Fourth, the plaque classifier is robust in many examples, but partly shows limited performance. The wide range of morphologies, which often cannot be unambiguously classified even by humans, further complicates the process. Moreover, a portion of the plaques was likely not sectioned representatively, e.g., the number of cored plaques is likely underestimated as the core is sometimes not visible depending on the sectional plane. Fifth, Aβ oligomers are not reflected in this study and small plaques (< 100 µm^2^) were also removed during preprocessing. Consequently, presumably clinically relevant deposits are missing in the downstream analysis and need clarification in further studies. Sixth, this study only covers a fraction of the factors that are directly or indirectly associated with Aβ deposition. Numerous components, such as lifestyle, education, secondary diagnoses, and blood vessel pathology, as well as details about the clinical presentation, are missing in this analysis. Finally, causal conclusions remain speculative due to the correlative, post-mortem attempt.

## Conclusion

Quantifying and classifying Aβ deposits in histological stains of Alzheimer’s disease cases, we found cortically dominated and, to a large extent, diffusely undifferentiated Aβ plaques. Only cored plaques were positively correlated with the entorhinal tau load. Female sex was associated with higher densities of diffuse plaques and ApoE4 genotype with denser plaque types. Earlier age at onset and death were associated with higher densities of diffuse light plaques. The effect sizes depended on the region and convergence of determinants. Compared to diffuse, small and compact plaques, cored plaques and CAA stand out statistically despite a relatively low density, suggesting special roles in the course of Alzheimer’s disease.

## Supplementary Information

Below is the link to the electronic supplementary material.

Supplementary file1 (PDF 7539 KB)

Supplementary file2 (CSV 98 KB)

## Data Availability

Supplementary Table S19, with column explanations in Table S18, provides mean and median regional Aβ deposit counts per mm² for the whole study cohort and specified sub-cohorts as templates for further correlative analyses. Code and models for Aβ deposit segmentation and classification are publicly available on GitHub (https://github.com/cor2ni/Abeta_histo_classifier). Code for this study was partially debugged, amended or generated with ChatGPT-4 and − 5. The authors reviewed, verified and adapted the code to ensure the intended function. Further supporting data and code applied in this study can be obtained from the corresponding author upon reasonable request.

## Abbreviations

α-syn: Alpha-Synuclein
Aβ: Amyloid beta
AD: Alzheimer’s disease
APP: Amyloid precursor protein
CAA: Cerebral amyloid angiopathy
CNN: Convolutional neural network
DAB: Diaminobenzidine
FDR: False discovery rate
IQR: Interquartile range
MFG: Middle frontal gyrus
