# Supplementary material for: Amyloid-β plaque diversity across brain regions in Alzheimer’s disease and its relation to major clinicopathological traits
Source: Acta Neuropathol. 2026 Oct 7;152(1):46. doi: 10.1007/s00401-026-03096-1 (PMC13645940; doi:10.1007/s00401-026-03096-1)
Supplement: Supplementary file 1 [file 401_2026_3096_MOESM1_ESM.pdf]

**Supplementary Tables and Figures for**

**Title: Amyloid-β plaque diversity across brain regions in Alzheimer's disease and its relation to major clinicopathological traits**

**Authors:** Antonia Neubauer[1,2,3], Paul Feyen[1,2], Susanna Pekrun [1,2], Sigrun Roeber[1,2], Viktoria Ruf [1,2], Felix L. Strübing[1,2], Jochen Herms[1,2,3]

**Affiliations:**
[1]*Institute of Neuropathology, LMU Medizin, Ludwig-Maximilians-Universität München, Munich, Germany*
[2]*German Center for Neurodegenerative Diseases (DZNE), Munich, Germany*
[3]*Munich Cluster for Systems Neurology (SyNergy), Munich, Germany*

**Corresponding author:** Antonia.Neubauer@med.uni-muenchen.de

## Table of Contents

### Supplementary Tables

***Table S1:*** *Overview of 23 annotated brain regions with size and annotation comments* ........ 3
***Table S2:*** *Median [first and third quartile] of Aβ deposit counts/mm² in each brain region* ........ 5
***Table S3:*** *Median [first and third quartile] of Aβ deposit count proportions in percent in each brain region* ........ 6
***Table S4:*** *Comparison of total Aβ deposit counts/mm² between brain regions* ........ 7
***Table S5:*** *Comparison of proportional loads of cored plaques across brain regions [%]* ........ 8
***Table S6:*** *Comparison of proportional loads of diffuse light plaques across brain regions [%]* ........ 9
***Table S7:*** *Comparison of proportional loads of diffuse dense plaques across brain regions [%]* ........ 10
***Table S8:*** *Comparison of proportional loads of compact plaques across brain regions [%]* ........ 11
***Table S9:*** *Comparison of proportional loads of cerebral amyloid angiopathy (CAA) across brain regions [%]* ........ 12
***Table S10:*** *Comparison of proportional loads of small dense plaques across brain regions [%]* ........ 13
***Table S11:*** *Comparison of total Aβ deposit counts/mm² between brain regions in a complete subset* ........ 14
***Table S12:*** *Comparison of proportional loads of cored plaques across brain regions [%] in a complete subset* ........ 15
***Table S13:*** *Comparison of proportional loads of diffuse light plaques across brain regions [%] in complete subset* ........ 16
***Table S14:*** *Comparison of proportional loads of diffuse dense plaques across brain regions [%] in complete subset* ........ 17
***Table S15:*** *Comparison of proportional loads of compact plaques across brain regions [%] in complete subset* ........ 18
***Table S16:*** *Comparison of proportional loads of cerebral amyloid angiopathy (CAA) across brain regions [%] in complete subset* ........ 19
***Table S17:*** *Comparison of proportional loads of small dense plaques across brain regions [%] in complete subset* ........ 20
***Table S18:*** *Explanation of column names of the separate supplementary csv Table S19* ........ 21

**Supplementary Figures**


***Fig. S1:*** *Overview of the study cohort* ........ 22
***Fig. S2:*** *Staining availability* ........ 23
***Fig. S3:*** *Convolutional neural network optimization* ........ 24
***Fig. S4:*** *Manual reclassification of deposits which were classified as 'cored plaques' or 'CAA'.* ........ 25
***Fig. S5:*** *Aβ deposit classes across regions with a data subset* ........ 26
***Fig. S6:*** *Aβ deposit counts per mm² separately for each deposit class and brain region* ........ 27
***Fig. S7:*** *Pearson correlation between regional deposit counts of all classes together (/mm²)* ........ 28
***Fig. S8:*** *P-values and n of the above Pearson correlation analysis* ........ 29
***Fig. S9:*** *Class-wise Aβ plaque correlations between brain regions* ........ 30
***Fig. S10:*** *Regional associations between Aβ deposit classes and total Aβ load* ........ 31
***Fig. S11:*** *This Fig. continues the preceding Fig.* ........ 32
***Fig. S12:*** *False discovery rate corrected p-values of Pearson correlation analyses* ........ 33
***Fig. S13:*** *Associations between deposit classes with correction for patient ID* ........ 34
***Fig. S14:*** *Associations between deposit classes and Aβ covered area* ........ 35
***Fig. S15:*** *Associations between Aβ deposit classes and other protein deposits* ........ 36
***Fig. S16:*** *Correlations between Aβ and tau load within cortical regions* ........ 37
***Fig. S17:*** *This Fig. continues the preceding Fig.* ........ 38
***Fig. S18:*** *Associations between Aβ deposit classes and genetic factors* ........ 39
***Fig. S19:*** *Associations between Aβ deposit classes and age* ........ 40
***Fig. S20:*** *Integrative analysis of potential Aβ plaque composition determinants for exemplary brain regions* ........ 41

**Supplementary Tables**

***Table S1:*** *Overview of 23 annotated brain regions with size and annotation comments*

*Representative areas without artifacts or large blood vessels were selected for all regions. For cortical regions, the annotation was aligned vertically to the brain surface to cover all cortical layers. "Number" refers to the numbering in Fig. 1 of the main manuscript. One "tile" equals 4096*4096 pixels (900 x 900 µm²). The contents of the table and this caption have been adopted from Neubauer et al. Alpha-synuclein deposition patterns in Alzheimer's disease: association with cortical amyloid beta and variable tau load. Acta Neuropathol 2025;150:46. https://doi.org/10.1007/s00401-025-02952-w. The region annotations follow the same protocol but are slightly reduced and resorted for this analysis; the table was extended by case availability and annotation sizes.*

| Number | Region | Comment | Label size (rough estimate) | Region cluster | Cases available per region | Median size of region annotations (± standard deviation) [mm²] | Range of size [mm²] |
|---|---|---|---|---|---|---|---|
| 1 | middle frontal gyrus | at the level of the nucleus accumbens | 3x0.5 tiles | cortical | 67 | 1.08 (±0.22) | 0.63–1.79 |
| 2 | sulcus of middle frontal gyrus | | 3x0.5 tiles | cortical | 65 | 0.87 (±0.18) | 0.57–1.41 |
| 3 | parietal gyrus | inferior parietal lobulus at the level of the splenium | 3x0.5 tiles | cortical | 50 | 1.01 (±0.13) | 0.71–1.31 |
| 4 | superior temporal gyrus | at the level of the amygdala | 3x0.5 tiles | cortical | 48 | 1.10 (±0.20) | 0.71–1.50 |
| 5 | sulcus between superior and middle temporal gyrus on the side of the middle gyrus | | 3x0.5 tiles | cortical | 46 | 0.82 (±0.17) | 0.53–1.20 |
| 6 | middle temporal gyrus | | 3x0.5 tiles | cortical | 47 | 1.02 (±0.20) | 0.58–1.49 |
| 7 | long and short insular gyri | at the level of basal ganglia with putamen, globus pallidus and corpus of the caudate nucleus | 3x0.5 tiles | cortical | 36 | 0.88 (±0.25) | 0.55–1.52 |
| 8 | striate area gyrus | Broca areal 17 | 3x0.5 tiles | cortical | 67 | 0.91 (±0.18) | 0.60–1.33 |
| 9 | striate area sulcus | | 3x0.5 tiles | cortical | 67 | 0.77 (±0.17) | 0.48–1.35 |
| 10 | parahippocampal gyrus | at the level of the lateral geniculate nucleus | 3x0.5 tiles | cortical | 61 | 0.87 (±0.21) | 0.42–1.71 |
| 11 | entorhinal cortex | | 3x0.5 tiles | cortical | 23 | 1.11 (±0.25) | 0.61–1.53 |
| 12 | amygdala (lateral nucleus) | at the level of (trans-)entorhinal cortex | 1x1 tile | subcortical | 24 | 0.75 (±0.04) | 0.72–0.89 |
| 13 | edge between ventral and dorsal claustrum | Same level as insular cortex | 1x1 tile | subcortical | 37 | 0.66 (±0.12) | 0.49–1.05 |
| 14 | putamen | | 1x1 tile | subcortical | 38 | 0.74 (±0.07) | 0.65–1.05 |
| 15 | mediodorsal nucleus of thalamus | at the level of substantia nigra | 1x1 tile | subcortical | 29 | 0.71 (±0.03) | 0.66–0.77 |
| 16 | lateral nucleus of thalamus | | 1x1 tile | subcortical | 29 | 0.72 (±0.03) | 0.66–0.77 |

| | | | | | | | |
|---|---|---|---|---|---|---|---|
| 17 | subiculum | at the level of the lateral geniculate nucleus | 1x1 tile | hippocampal | 64 | 0.73 (±0.21) | 0.33–1.39 |
| 18 | CA1 region of hippocampus | | 1x1 tile | hippocampal | 64 | 0.67 (±0.20) | 0.31–1.34 |
| 19 | CA2 region of hippocampus | | 1x1 tile | hippocampal | 58 | 0.45 (±0.17) | 0.24–0.98 |
| 20 | CA3 region of hippocampus | | 1x1 tile | hippocampal | 59 | 0.51 (±0.19) | 0.29–1.37 |
| 21 | CA4 region of hippocampus | | 1x1 tile | hippocampal | 64 | 0.73 (±0.14) | 0.47–1.48 |
| 22 | cerebellar cortex | at the level of the dentate nucleus | 1x1 tile | cerebellum | 68 | 0.52 (±0.11) | 0.26–0.89 |
| 23 | cerebellum, dentate nucleus | | 1x1 tile | cerebellum | 68 | 0.62 (±0.07) | 0.38–0.75 |

***Table S2:*** *Median [first and third quartile] of Aβ deposit counts/mm² in each brain region*

| Region | All deposits [/mm²] | Cored [/mm²] | Diffuse light [/mm²] | Diffuse dense [/mm²] | Compact [/mm²] | CAA [/mm²] | Small dense [/mm²] |
|---|---|---|---|---|---|---|---|
| Mid. frontal GY | 85.3 [52.4, 125.2] | 1.2 [0.0, 2.5] | 38.7 [24.5, 61.1] | 8.1 [3.3, 20.7] | 8.8 [3.2, 15.5] | 0.0 [0.0, 0.0] | 18.0 [9.7, 33.4] |
| Frontal SU | 104.8 [72.0, 169.5] | 1.7 [0.0, 3.8] | 30.1 [19.2, 48.3] | 17.0 [9.2, 40.1] | 15.9 [8.5, 28.3] | 0.0 [0.0, 1.1] | 25.3 [14.0, 52.6] |
| Parietal GY | 95.2 [62.1, 134.4] | 1.1 [0.0, 2.5] | 27.7 [13.7, 48.2] | 14.1 [5.5, 27.5] | 11.1 [6.9, 21.2] | 0.0 [0.0, 0.0] | 25.1 [15.3, 37.0] |
| Sup. temporal GY | 85.8 [43.3, 129.9] | 0.7 [0.0, 2.2] | 33.5 [13.3, 57.2] | 9.9 [2.8, 22.4] | 7.6 [2.3, 13.8] | 0.0 [0.0, 0.0] | 17.6 [7.9, 38.7] |
| Temporal SU | 83.8 [53.6, 119.5] | 0.9 [0.0, 1.8] | 32.7 [13.8, 51.6] | 12.8 [2.7, 30.1] | 11.3 [3.1, 17.1] | 0.0 [0.0, 0.0] | 19.8 [7.5, 29.8] |
| Mid. temporal GY | 86.8 [54.4, 112.1] | 0.9 [0.0, 1.8] | 34.9 [20.2, 61.1] | 7.9 [2.0, 22.2] | 6.9 [2.9, 14.1] | 0.0 [0.0, 0.8] | 16.1 [6.2, 28.1] |
| Insula cortex | 81.7 [48.6, 140.5] | 0.0 [0.0, 1.2] | 29.9 [15.8, 49.4] | 13.5 [4.2, 28.7] | 9.6 [4.9, 14.8] | 0.0 [0.0, 0.0] | 13.1 [9.4, 38.8] |
| Occipital GY | 42.7 [23.9, 77.2] | 0.8 [0.0, 1.8] | 17.1 [8.0, 30.4] | 6.9 [1.8, 14.9] | 4.5 [2.4, 8.5] | 0.0 [0.0, 0.8] | 6.8 [2.9, 17.5] |
| Occipital SU | 49.5 [30.9, 74.2] | 0.0 [0.0, 1.8] | 16.7 [9.4, 26.8] | 8.4 [3.2, 18.6] | 8.6 [3.9, 14.6] | 0.0 [0.0, 1.4] | 9.8 [4.6, 18.2] |
| Parahippocampal GY | 40.8 [25.1, 68.5] | 0.0 [0.0, 1.1] | 25.2 [15.2, 35.7] | 3.1 [0.0, 9.9] | 1.2 [0.0, 3.8] | 0.0 [0.0, 0.0] | 6.5 [2.8, 12.6] |
| Entorhinal GY | 28.3 [19.1, 45.5] | 0.0 [0.0, 0.4] | 17.6 [12.1, 27.7] | 1.6 [0.8, 3.2] | 1.6 [0.0, 4.4] | 0.0 [0.0, 0.0] | 2.0 [1.0, 3.9] |
| Amygdala | 31.7 [14.2, 44.3] | 0.0 [0.0, 1.3] | 14.4 [8.0, 27.1] | 1.8 [0.0, 9.9] | 1.3 [0.0, 4.6] | 0.0 [0.0, 0.0] | 2.0 [0.9, 4.4] |
| Claustrum | 24.4 [18.2, 49.5] | 0.0 [0.0, 1.2] | 15.0 [8.5, 21.5] | 1.7 [0.0, 5.4] | 1.1 [0.0, 3.3] | 0.0 [0.0, 0.0] | 4.2 [1.4, 8.1] |
| Putamen | 48.8 [23.0, 82.7] | 0.0 [0.0, 0.0] | 10.9 [2.5, 33.4] | 2.6 [0.0, 13.8] | 2.5 [0.0, 10.8] | 0.0 [0.0, 0.0] | 13.2 [2.9, 35.2] |
| Thal. med. nucl. | 12.8 [0.0, 56.6] | 0.0 [0.0, 0.0] | 10.0 [0.0, 37.0] | 0.0 [0.0, 2.8] | 0.0 [0.0, 0.0] | 0.0 [0.0, 0.0] | 1.4 [0.0, 10.3] |
| Thal. ventrolat. nucl. | 10.0 [1.4, 35.3] | 0.0 [0.0, 0.0] | 4.1 [0.0, 11.3] | 1.3 [0.0, 4.2] | 0.0 [0.0, 1.5] | 0.0 [0.0, 0.0] | 4.1 [0.0, 10.6] |
| Subiculum | 27.9 [14.0, 51.8] | 0.0 [0.0, 0.3] | 11.0 [7.1, 21.9] | 4.3 [0.0, 10.6] | 1.8 [0.0, 5.1] | 0.0 [0.0, 0.0] | 4.9 [1.4, 11.8] |
| CA1 | 12.0 [6.3, 33.9] | 0.0 [0.0, 0.0] | 4.6 [0.0, 13.0] | 2.1 [0.0, 5.7] | 0.6 [0.0, 2.7] | 0.0 [0.0, 0.0] | 2.2 [0.0, 8.3] |
| CA2 | 4.6 [0.0, 10.0] | 0.0 [0.0, 0.0] | 0.0 [0.0, 4.6] | 0.0 [0.0, 1.7] | 0.0 [0.0, 2.3] | 0.0 [0.0, 0.0] | 0.0 [0.0, 3.8] |
| CA3 | 7.3 [0.0, 20.6] | 0.0 [0.0, 0.0] | 0.0 [0.0, 2.9] | 2.2 [0.0, 6.0] | 0.0 [0.0, 2.4] | 0.0 [0.0, 0.0] | 0.0 [0.0, 4.3] |
| CA4 | 10.7 [3.8, 20.5] | 0.0 [0.0, 0.0] | 1.6 [0.0, 5.9] | 2.1 [0.0, 5.6] | 1.4 [0.0, 2.7] | 0.0 [0.0, 0.0] | 2.0 [0.0, 4.2] |
| Cbl. cortex | 6.3 [1.4, 20.8] | 0.0 [0.0, 0.0] | 1.8 [0.0, 10.8] | 1.5 [0.0, 2.5] | 0.0 [0.0, 0.0] | 0.0 [0.0, 0.0] | 1.7 [0.0, 3.9] |
| Cbl. dentate nucl. | 0.0 [0.0, 11.2] | 0.0 [0.0, 0.0] | 0.0 [0.0, 2.7] | 0.0 [0.0, 0.0] | 0.0 [0.0, 0.0] | 0.0 [0.0, 0.0] | 0.0 [0.0, 7.2] |

CA cornu ammonis (hippocampus), Cbl. cerebellum, GY gyrus, med. medial, mid. middle, nucl. nucleus, SU sulcus, Sup. superior, Thal thalamus, ventrolat. ventrolateral

***Table S3:*** *Median [first and third quartile] of Aβ deposit count proportions in percent in each brain region*

| Region | Cored [%] | Diffuse light [%] | Diffuse dense [%] | Compact [%] | CAA [%] | Small dense [%] |
|---|---|---|---|---|---|---|
| Mid. frontal GY | 2.0 [0.0, 3.2] | 51.6 [31.1, 63.2] | 10.3 [4.5, 20.2] | 9.5 [5.2, 14.0] | 0.0 [0.0, 0.0] | 23.5 [16.2, 34.2] |
| Frontal SU | 1.4 [0.0, 4.7] | 27.1 [19.4, 53.8] | 18.1 [12.2, 29.0] | 14.7 [8.5, 20.6] | 0.0 [0.0, 1.1] | 25.6 [16.6, 36.0] |
| Parietal GY | 1.6 [0.0, 2.8] | 37.4 [17.7, 56.7] | 15.5 [8.5, 24.2] | 11.6 [8.2, 20.7] | 0.0 [0.0, 0.0] | 27.9 [21.7, 34.7] |
| Sup. temporal GY | 0.5 [0.0, 3.6] | 42.8 [23.6, 69.9] | 14.1 [5.1, 20.0] | 8.8 [3.8, 15.5] | 0.0 [0.0, 0.0] | 24.6 [14.4, 34.4] |
| Temporal SU | 0.6 [0.0, 2.7] | 44.4 [26.7, 61.5] | 16.7 [5.0, 25.4] | 12.2 [4.7, 21.1] | 0.0 [0.0, 0.0] | 19.6 [10.4, 30.1] |
| Mid. temporal GY | 1.0 [0.0, 2.6] | 55.4 [35.2, 72.4] | 10.8 [3.4, 18.5] | 8.0 [3.0, 16.3] | 0.0 [0.0, 0.4] | 19.7 [12.8, 27.7] |
| Insula cortex | 0.0 [0.0, 2.1] | 35.6 [25.2, 53.8] | 19.4 [7.3, 28.9] | 13.0 [6.4, 18.2] | 0.0 [0.0, 0.0] | 23.8 [16.9, 31.9] |
| Occipital GY | 1.0 [0.0, 4.1] | 46.2 [24.4, 63.4] | 14.1 [7.2, 22.6] | 12.1 [3.9, 19.9] | 0.0 [0.0, 0.7] | 17.6 [10.4, 25.5] |
| Occipital SU | 0.0 [0.0, 4.2] | 36.4 [24.9, 52.9] | 18.9 [9.5, 25.0] | 15.3 [7.7, 25.0] | 0.0 [0.0, 1.3] | 19.8 [14.3, 24.7] |
| Parahippocampal GY | 0.0 [0.0, 1.9] | 65.2 [48.8, 84.0] | 6.5 [0.3, 15.8] | 3.7 [0.0, 10.3] | 0.0 [0.0, 0.0] | 15.2 [8.5, 25.0] |
| Entorhinal GY | 0.0 [0.0, 0.6] | 75.0 [66.2, 84.2] | 7.1 [2.0, 17.0] | 5.7 [0.0, 15.3] | 0.0 [0.0, 0.0] | 6.9 [4.0, 14.8] |
| Amygdala | 0.0 [0.0, 3.6] | 60.2 [26.1, 86.7] | 14.4 [0.0, 27.7] | 4.6 [0.0, 15.8] | 0.0 [0.0, 0.0] | 9.2 [1.8, 14.6] |
| Claustrum | 0.0 [0.0, 4.2] | 60.0 [42.9, 87.5] | 6.1 [0.0, 22.2] | 0.9 [0.0, 12.5] | 0.0 [0.0, 0.0] | 15.6 [4.2, 23.5] |
| Putamen | 0.0 [0.0, 0.0] | 24.2 [8.3, 70.5] | 9.0 [0.0, 18.6] | 8.3 [0.0, 15.0] | 0.0 [0.0, 0.0] | 40.0 [17.6, 50.0] |
| Thal. med. nucl. | 0.0 [0.0, 0.0] | 78.4 [57.4, 100.0] | 1.7 [0.0, 5.1] | 0.0 [0.0, 0.8] | 0.0 [0.0, 0.0] | 18.9 [0.0, 25.9] |
| Thal. ventrolat. nucl. | 0.0 [0.0, 3.5] | 46.4 [29.0, 74.3] | 9.2 [0.0, 20.0] | 0.0 [0.0, 6.5] | 0.0 [0.0, 0.0] | 29.6 [4.7, 43.3] |
| Subiculum | 0.0 [0.0, 1.3] | 46.8 [28.7, 73.4] | 16.3 [0.6, 29.4] | 6.2 [0.0, 12.5] | 0.0 [0.0, 0.0] | 16.5 [7.1, 26.8] |
| CA1 | 0.0 [0.0, 0.5] | 38.7 [16.7, 66.4] | 16.7 [0.0, 33.3] | 5.3 [0.0, 16.3] | 0.0 [0.0, 0.0] | 20.0 [8.8, 34.8] |
| CA2 | 0.0 [0.0, 0.0] | 33.3 [0.0, 58.6] | 0.0 [0.0, 18.3] | 0.0 [0.0, 20.0] | 0.0 [0.0, 0.0] | 37.5 [0.0, 51.9] |
| CA3 | 0.0 [0.0, 0.0] | 11.7 [0.0, 27.7] | 32.5 [10.5, 53.4] | 2.7 [0.0, 22.6] | 0.0 [0.0, 0.0] | 24.0 [6.7, 45.8] |
| CA4 | 0.0 [0.0, 0.0] | 28.0 [5.9, 50.0] | 22.7 [5.2, 40.9] | 9.1 [0.0, 24.0] | 0.0 [0.0, 0.0] | 22.2 [10.0, 40.0] |
| Cbl. cortex | 0.0 [0.0, 0.0] | 50.0 [5.0, 66.7] | 14.3 [0.0, 33.3] | 0.0 [0.0, 1.7] | 0.0 [0.0, 4.3] | 25.0 [5.6, 33.8] |
| Cbl. dentate nucl. | 0.0 [0.0, 0.0] | 28.6 [14.4, 90.0] | 0.0 [0.0, 0.0] | 0.0 [0.0, 0.0] | 0.0 [0.0, 0.0] | 53.3 [10.0, 82.2] |

CA cornu ammonis (hippocampus), Cbl. cerebellum, GY gyrus, med. medial, mid. middle, nucl. nucleus, SU sulcus, Sup. superior, Thal thalamus, ventrolat. ventrolateral

***Table S4:*** *Comparison of total Aβ deposit counts/mm² between brain regions*

*Kruskal-Wallis tests and Dunn's post hoc analysis with FDR Benjamini-Hochberg correction for multiple testing. Regions are indicated with background colors, significant, false discovery rate corrected p-values are labeled in green font color.*

| | MF_GY | F_SU | P_GY | ST_GY | T_SU | MT_GY | Insula | O_GY | O_SU | Phip_GY | Ent_GY | Amyg | Cla | Put | Thal_MNcl | Thal_VLNcl | Sub | CA1 | CA2 | CA3 | CA4 | Cbl_cor | Cbl_Dentncl |
|---|---|---|---|---|---|---|---|---|---|---|---|---|---|---|---|---|---|---|---|---|---|---|---|
| **MF_GY** | 1.0 | 0.27 | 0.54 | 0.67 | 0.92 | 0.83 | 0.72 | <0.01 | <0.01 | <0.01 | <0.01 | <0.01 | <0.01 | <0.01 | <0.01 | <0.01 | <0.01 | <0.01 | <0.01 | <0.01 | <0.01 | <0.01 | <0.01 |
| **F_SU** | 0.27 | 1.0 | 0.7 | 0.14 | 0.27 | 0.21 | 0.19 | <0.01 | <0.01 | <0.01 | <0.01 | <0.01 | <0.01 | <0.01 | <0.01 | <0.01 | <0.01 | <0.01 | <0.01 | <0.01 | <0.01 | <0.01 | <0.01 |
| **P_GY** | 0.54 | 0.7 | 1.0 | 0.32 | 0.52 | 0.44 | 0.38 | <0.01 | <0.01 | <0.01 | <0.01 | <0.01 | <0.01 | <0.01 | <0.01 | <0.01 | <0.01 | <0.01 | <0.01 | <0.01 | <0.01 | <0.01 | <0.01 |
| **ST_GY** | 0.67 | 0.14 | 0.32 | 1.0 | 0.77 | 0.85 | 0.98 | <0.01 | 0.049 | <0.01 | <0.01 | <0.01 | <0.01 | <0.01 | <0.01 | <0.01 | <0.01 | <0.01 | <0.01 | <0.01 | <0.01 | <0.01 | <0.01 |
| **T_SU** | 0.92 | 0.27 | 0.52 | 0.77 | 1.0 | 0.92 | 0.81 | <0.01 | 0.02 | <0.01 | <0.01 | <0.01 | <0.01 | <0.01 | <0.01 | <0.01 | <0.01 | <0.01 | <0.01 | <0.01 | <0.01 | <0.01 | <0.01 |
| **MT_GY** | 0.83 | 0.21 | 0.44 | 0.85 | 0.92 | 1.0 | 0.88 | <0.01 | 0.028 | <0.01 | <0.01 | <0.01 | <0.01 | <0.01 | <0.01 | <0.01 | <0.01 | <0.01 | <0.01 | <0.01 | <0.01 | <0.01 | <0.01 |
| **Insula** | 0.72 | 0.19 | 0.38 | 0.98 | 0.81 | 0.88 | 1.0 | 0.013 | 0.07 | <0.01 | <0.01 | <0.01 | <0.01 | 0.015 | <0.01 | <0.01 | <0.01 | <0.01 | <0.01 | <0.01 | <0.01 | <0.01 | <0.01 |
| **O_GY** | <0.01 | <0.01 | <0.01 | <0.01 | <0.01 | <0.01 | 0.013 | 1.0 | 0.49 | 0.74 | 0.17 | 0.16 | 0.07 | 0.81 | 0.013 | <0.01 | 0.12 | <0.01 | <0.01 | <0.01 | <0.01 | <0.01 | <0.01 |
| **O_SU** | <0.01 | <0.01 | <0.01 | 0.049 | 0.02 | 0.028 | 0.07 | 0.49 | 1.0 | 0.29 | 0.05 | 0.047 | 0.013 | 0.39 | <0.01 | <0.01 | 0.018 | <0.01 | <0.01 | <0.01 | <0.01 | <0.01 | <0.01 |
| **Phip_GY** | <0.01 | <0.01 | <0.01 | <0.01 | <0.01 | <0.01 | <0.01 | 0.74 | 0.29 | 1.0 | 0.27 | 0.26 | 0.15 | 0.97 | 0.032 | <0.01 | 0.24 | <0.01 | <0.01 | <0.01 | <0.01 | <0.01 | <0.01 |
| **Ent_GY** | <0.01 | <0.01 | <0.01 | <0.01 | <0.01 | <0.01 | <0.01 | 0.17 | 0.05 | 0.27 | 1.0 | 1.0 | 0.92 | 0.29 | 0.49 | 0.15 | 0.81 | 0.21 | <0.01 | 0.011 | 0.039 | <0.01 | <0.01 |
| **Amyg** | <0.01 | <0.01 | <0.01 | <0.01 | <0.01 | <0.01 | <0.01 | 0.16 | 0.047 | 0.26 | 1.0 | 1.0 | 0.92 | 0.28 | 0.49 | 0.15 | 0.81 | 0.2 | <0.01 | <0.01 | 0.036 | <0.01 | <0.01 |
| **Cla** | <0.01 | <0.01 | <0.01 | <0.01 | <0.01 | <0.01 | <0.01 | 0.07 | 0.013 | 0.15 | 0.92 | 0.92 | 1.0 | 0.18 | 0.51 | 0.14 | 0.68 | 0.18 | <0.01 | <0.01 | 0.022 | <0.01 | <0.01 |
| **Put** | <0.01 | <0.01 | <0.01 | <0.01 | <0.01 | <0.01 | 0.015 | 0.81 | 0.39 | 0.97 | 0.29 | 0.28 | 0.18 | 1.0 | 0.045 | <0.01 | 0.29 | <0.01 | <0.01 | <0.01 | <0.01 | <0.01 | <0.01 |
| **Thal_MNcl** | <0.01 | <0.01 | <0.01 | <0.01 | <0.01 | <0.01 | <0.01 | 0.013 | <0.01 | 0.032 | 0.49 | 0.49 | 0.51 | 0.045 | 1.0 | 0.46 | 0.25 | 0.66 | 0.018 | 0.07 | 0.2 | 0.06 | <0.01 |
| **Thal_VLNcl** | <0.01 | <0.01 | <0.01 | <0.01 | <0.01 | <0.01 | <0.01 | <0.01 | <0.01 | <0.01 | 0.15 | 0.15 | 0.14 | <0.01 | 0.46 | 1.0 | 0.033 | 0.7 | 0.16 | 0.38 | 0.72 | 0.36 | 0.04 |
| **Sub** | <0.01 | <0.01 | <0.01 | <0.01 | <0.01 | <0.01 | <0.01 | 0.12 | 0.018 | 0.24 | 0.81 | 0.81 | 0.68 | 0.29 | 0.25 | 0.033 | 1.0 | 0.032 | <0.01 | <0.01 | <0.01 | <0.01 | <0.01 |
| **CA1** | <0.01 | <0.01 | <0.01 | <0.01 | <0.01 | <0.01 | <0.01 | <0.01 | <0.01 | <0.01 | 0.21 | 0.2 | 0.18 | <0.01 | 0.66 | 0.7 | 0.032 | 1.0 | 0.018 | 0.1 | 0.31 | 0.08 | <0.01 |
| **CA2** | <0.01 | <0.01 | <0.01 | <0.01 | <0.01 | <0.01 | <0.01 | <0.01 | <0.01 | <0.01 | <0.01 | <0.01 | <0.01 | <0.01 | 0.018 | 0.16 | <0.01 | 0.018 | 1.0 | 0.54 | 0.2 | 0.54 | 0.5 |
| **CA3** | <0.01 | <0.01 | <0.01 | <0.01 | <0.01 | <0.01 | <0.01 | <0.01 | <0.01 | <0.01 | 0.011 | <0.01 | <0.01 | <0.01 | 0.07 | 0.38 | <0.01 | 0.1 | 0.54 | 1.0 | 0.53 | 0.98 | 0.18 |
| **CA4** | <0.01 | <0.01 | <0.01 | <0.01 | <0.01 | <0.01 | <0.01 | <0.01 | <0.01 | <0.01 | 0.039 | 0.036 | 0.022 | <0.01 | 0.2 | 0.72 | <0.01 | 0.31 | 0.2 | 0.53 | 1.0 | 0.5 | 0.036 |
| **Cbl_cor** | <0.01 | <0.01 | <0.01 | <0.01 | <0.01 | <0.01 | <0.01 | <0.01 | <0.01 | <0.01 | <0.01 | <0.01 | <0.01 | <0.01 | 0.06 | 0.36 | <0.01 | 0.08 | 0.54 | 0.98 | 0.5 | 1.0 | 0.17 |
| **Cbl_Dentncl** | <0.01 | <0.01 | <0.01 | <0.01 | <0.01 | <0.01 | <0.01 | <0.01 | <0.01 | <0.01 | <0.01 | <0.01 | <0.01 | <0.01 | <0.01 | 0.04 | <0.01 | <0.01 | 0.5 | 0.18 | 0.036 | 0.17 | 1.0 |

Amyg amygdala, Cbl cerebellum, Cla claustrum, cor cortex, Dentncl dentate nucleus, Ent entorhinal, F frontal, GY gyrus, MF middle frontal, MNcl medial nucleus, MT middle temporal, O occipital, P parietal, Phip parahippocampal, Put putamen, ST superior temporal, SU sulcus, Sub subiculum, T temporal, Thal thalamus, VlNcl ventrolateral nucleus

***Table S5:*** *Comparison of proportional loads of cored plaques across brain regions [%]*
*Kruskal-Wallis tests and Dunn's post hoc analysis with FDR Benjamini-Hochberg correction for multiple testing. Regions are indicated with background colors, significant, false discovery rate corrected p-values are labeled in green font color.*

| | MF_GY | F_SU | P_GY | ST_GY | T_SU | MT_GY | Insula | O_GY | O_SU | Phip_GY | Ent_GY | Amyg | Cla | Put | Thal_MNcl | Thal_VLNcl | Sub | CA1 | CA2 | CA3 | CA4 | Cbl_cor | Cbl_Dentncl |
|---|---|---|---|---|---|---|---|---|---|---|---|---|---|---|---|---|---|---|---|---|---|---|---|
| **MF_GY** | 1.0 | 0.93 | 0.84 | 0.17 | 0.17 | 0.25 | 0.08 | 0.35 | 0.19 | <0.01 | 0.01 | 0.046 | 0.015 | <0.01 | <0.01 | 0.05 | <0.01 | <0.01 | <0.01 | <0.01 | <0.01 | <0.01 | <0.01 |
| **F_SU** | 0.93 | 1.0 | 0.77 | 0.14 | 0.14 | 0.21 | 0.06 | 0.3 | 0.15 | <0.01 | <0.01 | 0.039 | 0.012 | <0.01 | <0.01 | 0.046 | <0.01 | <0.01 | <0.01 | <0.01 | <0.01 | <0.01 | <0.01 |
| **P_GY** | 0.84 | 0.77 | 1.0 | 0.3 | 0.3 | 0.4 | 0.15 | 0.57 | 0.35 | <0.01 | 0.024 | 0.09 | 0.038 | <0.01 | <0.01 | 0.1 | <0.01 | <0.01 | <0.01 | <0.01 | <0.01 | <0.01 | <0.01 |
| **ST_GY** | 0.17 | 0.14 | 0.3 | 1.0 | 1.0 | 0.88 | 0.71 | 0.68 | 0.88 | 0.14 | 0.18 | 0.45 | 0.32 | <0.01 | <0.01 | 0.47 | 0.07 | 0.07 | <0.01 | <0.01 | <0.01 | <0.01 | <0.01 |
| **T_SU** | 0.17 | 0.14 | 0.3 | 1.0 | 1.0 | 0.88 | 0.71 | 0.68 | 0.88 | 0.15 | 0.18 | 0.46 | 0.33 | <0.01 | <0.01 | 0.47 | 0.07 | 0.08 | <0.01 | <0.01 | <0.01 | <0.01 | <0.01 |
| **MT_GY** | 0.25 | 0.21 | 0.4 | 0.88 | 0.88 | 1.0 | 0.59 | 0.8 | 0.99 | 0.1 | 0.14 | 0.37 | 0.25 | <0.01 | <0.01 | 0.38 | 0.043 | 0.046 | <0.01 | <0.01 | <0.01 | <0.01 | <0.01 |
| **Insula** | 0.08 | 0.06 | 0.15 | 0.71 | 0.71 | 0.59 | 1.0 | 0.37 | 0.58 | 0.41 | 0.38 | 0.76 | 0.65 | <0.01 | 0.034 | 0.76 | 0.25 | 0.25 | <0.01 | 0.032 | <0.01 | <0.01 | <0.01 |
| **O_GY** | 0.35 | 0.3 | 0.57 | 0.68 | 0.68 | 0.8 | 0.37 | 1.0 | 0.77 | 0.032 | 0.07 | 0.22 | 0.12 | <0.01 | <0.01 | 0.24 | 0.011 | 0.013 | <0.01 | <0.01 | <0.01 | <0.01 | <0.01 |
| **O_SU** | 0.19 | 0.15 | 0.35 | 0.88 | 0.88 | 0.99 | 0.58 | 0.77 | 1.0 | 0.07 | 0.12 | 0.35 | 0.22 | <0.01 | <0.01 | 0.37 | 0.029 | 0.032 | <0.01 | <0.01 | <0.01 | <0.01 | <0.01 |
| **Phip_GY** | <0.01 | <0.01 | <0.01 | 0.14 | 0.15 | 0.1 | 0.41 | 0.032 | 0.07 | 1.0 | 0.84 | 0.77 | 0.81 | 0.026 | 0.13 | 0.78 | 0.77 | 0.77 | 0.013 | 0.14 | 0.019 | <0.01 | <0.01 |
| **Ent_GY** | 0.01 | <0.01 | 0.024 | 0.18 | 0.18 | 0.14 | 0.38 | 0.07 | 0.12 | 0.84 | 1.0 | 0.68 | 0.71 | 0.13 | 0.3 | 0.69 | 1.0 | 0.99 | 0.09 | 0.37 | 0.13 | 0.09 | 0.06 |
| **Amyg** | 0.046 | 0.039 | 0.09 | 0.45 | 0.46 | 0.37 | 0.76 | 0.22 | 0.35 | 0.77 | 0.68 | 1.0 | 0.95 | 0.034 | 0.12 | 1.0 | 0.6 | 0.6 | 0.022 | 0.14 | 0.032 | 0.02 | 0.016 |
| **Cla** | 0.015 | 0.012 | 0.038 | 0.32 | 0.33 | 0.25 | 0.65 | 0.12 | 0.22 | 0.81 | 0.71 | 0.95 | 1.0 | 0.024 | 0.1 | 0.95 | 0.6 | 0.6 | 0.012 | 0.12 | 0.019 | 0.01 | <0.01 |
| **Put** | <0.01 | <0.01 | <0.01 | <0.01 | <0.01 | <0.01 | <0.01 | <0.01 | <0.01 | 0.026 | 0.13 | 0.034 | 0.024 | 1.0 | 0.79 | 0.039 | 0.05 | 0.06 | 0.93 | 0.52 | 0.88 | 0.99 | 0.77 |
| **Thal_MNcl** | <0.01 | <0.01 | <0.01 | <0.01 | <0.01 | <0.01 | 0.034 | <0.01 | <0.01 | 0.13 | 0.3 | 0.12 | 0.1 | 0.79 | 1.0 | 0.13 | 0.21 | 0.22 | 0.72 | 0.8 | 0.88 | 0.77 | 0.59 |
| **Thal_VLNcl** | 0.05 | 0.046 | 0.1 | 0.47 | 0.47 | 0.38 | 0.76 | 0.24 | 0.37 | 0.78 | 0.69 | 1.0 | 0.95 | 0.039 | 0.13 | 1.0 | 0.61 | 0.6 | 0.025 | 0.15 | 0.037 | 0.024 | 0.019 |
| **Sub** | <0.01 | <0.01 | <0.01 | 0.07 | 0.07 | 0.043 | 0.25 | 0.011 | 0.029 | 0.77 | 1.0 | 0.6 | 0.6 | 0.05 | 0.21 | 0.61 | 1.0 | 0.99 | 0.028 | 0.25 | 0.041 | 0.024 | 0.021 |
| **CA1** | <0.01 | <0.01 | <0.01 | 0.07 | 0.08 | 0.046 | 0.25 | 0.013 | 0.032 | 0.77 | 0.99 | 0.6 | 0.6 | 0.06 | 0.22 | 0.6 | 0.99 | 1.0 | 0.034 | 0.27 | 0.05 | 0.029 | 0.025 |
| **CA2** | <0.01 | <0.01 | <0.01 | <0.01 | <0.01 | <0.01 | <0.01 | <0.01 | <0.01 | 0.013 | 0.09 | 0.022 | 0.012 | 0.93 | 0.72 | 0.025 | 0.028 | 0.034 | 1.0 | 0.41 | 0.79 | 0.94 | 0.84 |
| **CA3** | <0.01 | <0.01 | <0.01 | <0.01 | <0.01 | <0.01 | 0.032 | <0.01 | <0.01 | 0.14 | 0.37 | 0.14 | 0.12 | 0.52 | 0.8 | 0.15 | 0.25 | 0.27 | 0.41 | 1.0 | 0.59 | 0.44 | 0.31 |
| **CA4** | <0.01 | <0.01 | <0.01 | <0.01 | <0.01 | <0.01 | <0.01 | <0.01 | <0.01 | 0.019 | 0.13 | 0.032 | 0.019 | 0.88 | 0.88 | 0.037 | 0.041 | 0.05 | 0.79 | 0.59 | 1.0 | 0.84 | 0.63 |
| **Cbl_cor** | <0.01 | <0.01 | <0.01 | <0.01 | <0.01 | <0.01 | <0.01 | <0.01 | <0.01 | <0.01 | 0.09 | 0.02 | 0.01 | 0.99 | 0.77 | 0.024 | 0.024 | 0.029 | 0.94 | 0.44 | 0.84 | 1.0 | 0.77 |
| **Cbl_Dentncl** | <0.01 | <0.01 | <0.01 | <0.01 | <0.01 | <0.01 | <0.01 | <0.01 | <0.01 | <0.01 | 0.06 | 0.016 | <0.01 | 0.77 | 0.59 | 0.019 | 0.021 | 0.025 | 0.84 | 0.31 | 0.63 | 0.77 | 1.0 |

Amyg amygdala, Cbl cerebellum, Cla claustrum, cor cortex, Dentncl dentate nucleus, Ent entorhinal, F frontal, GY gyrus, MF middle frontal, MNcl medial nucleus, MT middle temporal, O occipital, P parietal, Phip parahippocampal, Put putamen, ST superior temporal, SU sulcus, Sub subiculum, T temporal, Thal thalamus, VlNcl ventrolateral nucleus

***Table S6:*** *Comparison of proportional loads of diffuse light plaques across brain regions [%]*
*Kruskal-Wallis tests and Dunn's post hoc analysis with FDR Benjamini-Hochberg correction for multiple testing. Regions are indicated with background colors, significant, false discovery rate corrected p-values are labeled in green font color.*

| | MF_GY | F_SU | P_GY | ST_GY | T_SU | MT_GY | Insula | O_GY | O_SU | Phip_GY | Ent_GY | Amyg | Cla | Put | Thal_MNcl | Thal_VLNcl | Sub | CA1 | CA2 | CA3 | CA4 | Cbl_cor | Cbl_Dentncl |
|---|---|---|---|---|---|---|---|---|---|---|---|---|---|---|---|---|---|---|---|---|---|---|---|
| **MF_GY** | 1.0 | 0.016 | 0.12 | 0.83 | 0.68 | 0.66 | 0.38 | 0.67 | 0.14 | 0.013 | 0.04 | 0.57 | 0.12 | 0.2 | <0.01 | 0.98 | 0.91 | 0.17 | 0.06 | <0.01 | <0.01 | 0.26 | 0.5 |
| **F_SU** | 0.016 | 1.0 | 0.6 | 0.06 | 0.11 | <0.01 | 0.35 | 0.07 | 0.45 | <0.01 | <0.01 | 0.016 | <0.01 | 0.62 | <0.01 | 0.11 | 0.013 | 0.45 | 0.88 | 0.022 | 0.56 | 0.36 | 0.3 |
| **P_GY** | 0.12 | 0.6 | 1.0 | 0.25 | 0.35 | 0.05 | 0.69 | 0.3 | 0.88 | <0.01 | <0.01 | 0.07 | <0.01 | 0.97 | <0.01 | 0.29 | 0.1 | 0.86 | 0.75 | <0.01 | 0.27 | 0.75 | 0.62 |
| **ST_GY** | 0.83 | 0.06 | 0.25 | 1.0 | 0.86 | 0.53 | 0.55 | 0.86 | 0.29 | 0.011 | 0.031 | 0.47 | 0.1 | 0.33 | <0.01 | 0.88 | 0.75 | 0.33 | 0.14 | <0.01 | 0.013 | 0.43 | 0.66 |
| **T_SU** | 0.68 | 0.11 | 0.35 | 0.86 | 1.0 | 0.41 | 0.67 | 0.98 | 0.4 | <0.01 | 0.02 | 0.37 | 0.06 | 0.44 | <0.01 | 0.77 | 0.62 | 0.44 | 0.21 | <0.01 | 0.026 | 0.56 | 0.77 |
| **MT_GY** | 0.66 | <0.01 | 0.05 | 0.53 | 0.41 | 1.0 | 0.2 | 0.38 | 0.06 | 0.09 | 0.13 | 0.85 | 0.34 | 0.1 | 0.043 | 0.74 | 0.74 | 0.08 | 0.027 | <0.01 | <0.01 | 0.12 | 0.3 |
| **Insula** | 0.38 | 0.35 | 0.69 | 0.55 | 0.67 | 0.2 | 1.0 | 0.64 | 0.77 | <0.01 | <0.01 | 0.2 | 0.022 | 0.75 | <0.01 | 0.53 | 0.34 | 0.81 | 0.49 | <0.01 | 0.13 | 0.91 | 0.91 |
| **O_GY** | 0.67 | 0.07 | 0.3 | 0.86 | 0.98 | 0.38 | 0.64 | 1.0 | 0.34 | <0.01 | 0.014 | 0.35 | 0.044 | 0.39 | <0.01 | 0.77 | 0.6 | 0.38 | 0.17 | <0.01 | 0.013 | 0.51 | 0.75 |
| **O_SU** | 0.14 | 0.45 | 0.88 | 0.29 | 0.4 | 0.06 | 0.77 | 0.34 | 1.0 | <0.01 | <0.01 | 0.09 | <0.01 | 0.92 | <0.01 | 0.33 | 0.11 | 0.97 | 0.63 | <0.01 | 0.16 | 0.85 | 0.69 |
| **Phip_GY** | 0.013 | <0.01 | <0.01 | 0.011 | <0.01 | 0.09 | <0.01 | <0.01 | <0.01 | 1.0 | 0.83 | 0.29 | 0.66 | <0.01 | 0.5 | 0.09 | 0.021 | <0.01 | <0.01 | <0.01 | <0.01 | <0.01 | <0.01 |
| **Ent_GY** | 0.04 | <0.01 | <0.01 | 0.031 | 0.02 | 0.13 | <0.01 | 0.014 | <0.01 | 0.83 | 1.0 | 0.28 | 0.58 | <0.01 | 0.7 | 0.1 | 0.05 | <0.01 | <0.01 | <0.01 | <0.01 | <0.01 | 0.014 |
| **Amyg** | 0.57 | 0.016 | 0.07 | 0.47 | 0.37 | 0.85 | 0.2 | 0.35 | 0.09 | 0.29 | 0.28 | 1.0 | 0.56 | 0.11 | 0.12 | 0.64 | 0.63 | 0.1 | 0.041 | <0.01 | <0.01 | 0.14 | 0.28 |
| **Cla** | 0.12 | <0.01 | <0.01 | 0.1 | 0.06 | 0.34 | 0.022 | 0.044 | <0.01 | 0.66 | 0.58 | 0.56 | 1.0 | <0.01 | 0.31 | 0.25 | 0.15 | <0.01 | <0.01 | <0.01 | <0.01 | <0.01 | 0.043 |
| **Put** | 0.2 | 0.62 | 0.97 | 0.33 | 0.44 | 0.1 | 0.75 | 0.39 | 0.92 | <0.01 | <0.01 | 0.11 | <0.01 | 1.0 | <0.01 | 0.35 | 0.17 | 0.91 | 0.75 | 0.012 | 0.3 | 0.81 | 0.67 |
| **Thal_MNcl** | <0.01 | <0.01 | <0.01 | <0.01 | <0.01 | 0.043 | <0.01 | <0.01 | <0.01 | 0.5 | 0.7 | 0.12 | 0.31 | <0.01 | 1.0 | 0.037 | 0.014 | <0.01 | <0.01 | <0.01 | <0.01 | <0.01 | <0.01 |
| **Thal_VLNcl** | 0.98 | 0.11 | 0.29 | 0.88 | 0.77 | 0.74 | 0.53 | 0.77 | 0.33 | 0.09 | 0.1 | 0.64 | 0.25 | 0.35 | 0.037 | 1.0 | 0.91 | 0.35 | 0.18 | <0.01 | 0.037 | 0.44 | 0.62 |
| **Sub** | 0.91 | 0.013 | 0.1 | 0.75 | 0.62 | 0.74 | 0.34 | 0.6 | 0.11 | 0.021 | 0.05 | 0.63 | 0.15 | 0.17 | 0.014 | 0.91 | 1.0 | 0.14 | 0.05 | <0.01 | <0.01 | 0.22 | 0.44 |
| **CA1** | 0.17 | 0.45 | 0.86 | 0.33 | 0.44 | 0.08 | 0.81 | 0.38 | 0.97 | <0.01 | <0.01 | 0.1 | <0.01 | 0.91 | <0.01 | 0.35 | 0.14 | 1.0 | 0.63 | <0.01 | 0.17 | 0.88 | 0.72 |
| **CA2** | 0.06 | 0.88 | 0.75 | 0.14 | 0.21 | 0.027 | 0.49 | 0.17 | 0.63 | <0.01 | <0.01 | 0.041 | <0.01 | 0.75 | <0.01 | 0.18 | 0.05 | 0.63 | 1.0 | 0.029 | 0.51 | 0.53 | 0.43 |
| **CA3** | <0.01 | 0.022 | <0.01 | <0.01 | <0.01 | <0.01 | <0.01 | <0.01 | <0.01 | <0.01 | <0.01 | <0.01 | <0.01 | 0.012 | <0.01 | <0.01 | <0.01 | <0.01 | 0.029 | 1.0 | 0.12 | <0.01 | <0.01 |
| **CA4** | <0.01 | 0.56 | 0.27 | 0.013 | 0.026 | <0.01 | 0.13 | 0.013 | 0.16 | <0.01 | <0.01 | <0.01 | <0.01 | 0.3 | <0.01 | 0.037 | <0.01 | 0.17 | 0.51 | 0.12 | 1.0 | 0.12 | 0.11 |
| **Cbl_cor** | 0.26 | 0.36 | 0.75 | 0.43 | 0.56 | 0.12 | 0.91 | 0.51 | 0.85 | <0.01 | <0.01 | 0.14 | <0.01 | 0.81 | <0.01 | 0.44 | 0.22 | 0.88 | 0.53 | <0.01 | 0.12 | 1.0 | 0.82 |
| **Cbl_Dentncl** | 0.5 | 0.3 | 0.62 | 0.66 | 0.77 | 0.3 | 0.91 | 0.75 | 0.69 | <0.01 | 0.014 | 0.28 | 0.043 | 0.67 | <0.01 | 0.62 | 0.44 | 0.72 | 0.43 | <0.01 | 0.11 | 0.82 | 1.0 |

Amyg amygdala, Cbl cerebellum, Cla claustrum, cor cortex, Dentncl dentate nucleus, Ent entorhinal, F frontal, GY gyrus, MF middle frontal, MNcl medial nucleus, MT middle temporal, O occipital, P parietal, Phip parahippocampal, Put putamen, ST superior temporal, SU sulcus, Sub subiculum, T temporal, Thal thalamus, VlNcl ventrolateral nucleus

***Table S7:*** *Comparison of proportional loads of diffuse dense plaques across brain regions [%]*
*Kruskal-Wallis tests and Dunn's post hoc analysis with FDR Benjamini-Hochberg correction for multiple testing. Regions are indicated with background colors, significant, false discovery rate corrected p-values are labeled in green font color.*

| | MF_GY | F_SU | P_GY | ST_GY | T_SU | MT_GY | Insula | O_GY | O_SU | Phip_GY | Ent_GY | Amyg | Cla | Put | Thal_MNcl | Thal_VLNcl | Sub | CA1 | CA2 | CA3 | CA4 | Cbl_cor | Cbl_Dentncl |
|---|---|---|---|---|---|---|---|---|---|---|---|---|---|---|---|---|---|---|---|---|---|---|---|
| **MF_GY** | 1.0 | 0.016 | 0.3 | 0.94 | 0.33 | 0.83 | 0.12 | 0.3 | 0.06 | 0.24 | 0.65 | 0.73 | 0.59 | 0.63 | 0.016 | 0.75 | 0.35 | 0.28 | 0.17 | <0.01 | 0.022 | 0.47 | <0.01 |
| **F_SU** | 0.016 | 1.0 | 0.28 | 0.034 | 0.27 | 0.014 | 0.74 | 0.21 | 0.66 | <0.01 | 0.022 | 0.21 | <0.01 | 0.012 | <0.01 | 0.034 | 0.2 | 0.28 | <0.01 | 0.24 | 0.98 | 0.17 | <0.01 |
| **P_GY** | 0.3 | 0.28 | 1.0 | 0.41 | 0.98 | 0.24 | 0.59 | 0.95 | 0.55 | 0.024 | 0.22 | 0.72 | 0.16 | 0.18 | <0.01 | 0.27 | 0.91 | 0.98 | 0.018 | 0.026 | 0.31 | 0.81 | <0.01 |
| **ST_GY** | 0.94 | 0.034 | 0.41 | 1.0 | 0.44 | 0.76 | 0.18 | 0.43 | 0.12 | 0.23 | 0.59 | 0.8 | 0.55 | 0.58 | 0.016 | 0.7 | 0.47 | 0.38 | 0.17 | <0.01 | 0.05 | 0.58 | <0.01 |
| **T_SU** | 0.33 | 0.27 | 0.98 | 0.44 | 1.0 | 0.26 | 0.58 | 0.98 | 0.53 | 0.031 | 0.23 | 0.75 | 0.18 | 0.2 | <0.01 | 0.28 | 0.94 | 0.97 | 0.022 | 0.026 | 0.3 | 0.85 | <0.01 |
| **MT_GY** | 0.83 | 0.014 | 0.24 | 0.76 | 0.26 | 1.0 | 0.09 | 0.24 | 0.045 | 0.41 | 0.8 | 0.59 | 0.79 | 0.81 | 0.034 | 0.9 | 0.27 | 0.22 | 0.28 | <0.01 | 0.019 | 0.36 | <0.01 |
| **Insula** | 0.12 | 0.74 | 0.59 | 0.18 | 0.58 | 0.09 | 1.0 | 0.52 | 0.98 | <0.01 | 0.09 | 0.41 | 0.05 | 0.06 | <0.01 | 0.13 | 0.48 | 0.59 | <0.01 | 0.17 | 0.76 | 0.43 | <0.01 |
| **O_GY** | 0.3 | 0.21 | 0.95 | 0.43 | 0.98 | 0.24 | 0.52 | 1.0 | 0.44 | 0.021 | 0.22 | 0.76 | 0.16 | 0.18 | <0.01 | 0.28 | 0.97 | 0.94 | 0.016 | 0.016 | 0.24 | 0.88 | <0.01 |
| **O_SU** | 0.06 | 0.66 | 0.55 | 0.12 | 0.53 | 0.045 | 0.98 | 0.44 | 1.0 | <0.01 | 0.06 | 0.36 | 0.025 | 0.034 | <0.01 | 0.09 | 0.41 | 0.55 | <0.01 | 0.1 | 0.7 | 0.36 | <0.01 |
| **Phip_GY** | 0.24 | <0.01 | 0.024 | 0.23 | 0.031 | 0.41 | <0.01 | 0.021 | <0.01 | 1.0 | 0.75 | 0.21 | 0.68 | 0.67 | 0.17 | 0.66 | 0.026 | 0.02 | 0.76 | <0.01 | <0.01 | 0.05 | <0.01 |
| **Ent_GY** | 0.65 | 0.022 | 0.22 | 0.59 | 0.23 | 0.8 | 0.09 | 0.22 | 0.06 | 0.75 | 1.0 | 0.48 | 0.98 | 0.98 | 0.14 | 0.94 | 0.24 | 0.21 | 0.57 | <0.01 | 0.027 | 0.3 | <0.01 |
| **Amyg** | 0.73 | 0.21 | 0.72 | 0.8 | 0.75 | 0.59 | 0.41 | 0.76 | 0.36 | 0.21 | 0.48 | 1.0 | 0.44 | 0.46 | 0.019 | 0.55 | 0.8 | 0.7 | 0.15 | 0.025 | 0.23 | 0.88 | <0.01 |
| **Cla** | 0.59 | <0.01 | 0.16 | 0.55 | 0.18 | 0.79 | 0.05 | 0.16 | 0.025 | 0.68 | 0.98 | 0.44 | 1.0 | 0.98 | 0.09 | 0.95 | 0.18 | 0.14 | 0.48 | <0.01 | 0.012 | 0.24 | <0.01 |
| **Put** | 0.63 | 0.012 | 0.18 | 0.58 | 0.2 | 0.81 | 0.06 | 0.18 | 0.034 | 0.67 | 0.98 | 0.46 | 0.98 | 1.0 | 0.09 | 0.97 | 0.21 | 0.17 | 0.48 | <0.01 | 0.016 | 0.26 | <0.01 |
| **Thal_MNcl** | 0.016 | <0.01 | <0.01 | 0.016 | <0.01 | 0.034 | <0.01 | <0.01 | <0.01 | 0.17 | 0.14 | 0.019 | 0.09 | 0.09 | 1.0 | 0.12 | <0.01 | <0.01 | 0.29 | <0.01 | <0.01 | <0.01 | 0.4 |
| **Thal_VLNcl** | 0.75 | 0.034 | 0.27 | 0.7 | 0.28 | 0.9 | 0.13 | 0.28 | 0.09 | 0.66 | 0.94 | 0.55 | 0.95 | 0.97 | 0.12 | 1.0 | 0.3 | 0.25 | 0.49 | <0.01 | 0.043 | 0.37 | <0.01 |
| **Sub** | 0.35 | 0.2 | 0.91 | 0.47 | 0.94 | 0.27 | 0.48 | 0.97 | 0.41 | 0.026 | 0.24 | 0.8 | 0.18 | 0.21 | <0.01 | 0.3 | 1.0 | 0.9 | 0.02 | 0.015 | 0.23 | 0.93 | <0.01 |
| **CA1** | 0.28 | 0.28 | 0.98 | 0.38 | 0.97 | 0.22 | 0.59 | 0.94 | 0.55 | 0.02 | 0.21 | 0.7 | 0.14 | 0.17 | <0.01 | 0.25 | 0.9 | 1.0 | 0.016 | 0.025 | 0.31 | 0.8 | <0.01 |
| **CA2** | 0.17 | <0.01 | 0.018 | 0.17 | 0.022 | 0.28 | <0.01 | 0.016 | <0.01 | 0.76 | 0.57 | 0.15 | 0.48 | 0.48 | 0.29 | 0.49 | 0.02 | 0.016 | 1.0 | <0.01 | <0.01 | 0.034 | 0.019 |
| **CA3** | <0.01 | 0.24 | 0.026 | <0.01 | 0.026 | <0.01 | 0.17 | 0.016 | 0.1 | <0.01 | <0.01 | 0.025 | <0.01 | <0.01 | <0.01 | <0.01 | 0.015 | 0.025 | <0.01 | 1.0 | 0.24 | 0.014 | <0.01 |
| **CA4** | 0.022 | 0.98 | 0.31 | 0.05 | 0.3 | 0.019 | 0.76 | 0.24 | 0.7 | <0.01 | 0.027 | 0.23 | 0.012 | 0.016 | <0.01 | 0.043 | 0.23 | 0.31 | <0.01 | 0.24 | 1.0 | 0.21 | <0.01 |
| **Cbl_cor** | 0.47 | 0.17 | 0.81 | 0.58 | 0.85 | 0.36 | 0.43 | 0.88 | 0.36 | 0.05 | 0.3 | 0.88 | 0.24 | 0.26 | <0.01 | 0.37 | 0.93 | 0.8 | 0.034 | 0.014 | 0.21 | 1.0 | <0.01 |
| **Cbl_Dentncl** | <0.01 | <0.01 | <0.01 | <0.01 | <0.01 | <0.01 | <0.01 | <0.01 | <0.01 | <0.01 | <0.01 | <0.01 | <0.01 | <0.01 | 0.4 | <0.01 | <0.01 | <0.01 | 0.019 | <0.01 | <0.01 | <0.01 | 1.0 |

Amyg amygdala, Cbl cerebellum, Cla claustrum, cor cortex, Dentncl dentate nucleus, Ent entorhinal, F frontal, GY gyrus, MF middle frontal, MNcl medial nucleus, MT middle temporal, O occipital, P parietal, Phip parahippocampal, Put putamen, ST superior temporal, SU sulcus, Sub subiculum, T temporal, Thal thalamus, VlNcl ventrolateral nucleus

***Table S8:*** *Comparison of proportional loads of compact plaques across brain regions [%]*
*Kruskal-Wallis tests and Dunn's post hoc analysis with FDR Benjamini-Hochberg correction for multiple testing. Regions are indicated with background colors, significant, false discovery rate corrected p-values are labeled in green font color.*

| | MF_GY | F_SU | P_GY | ST_GY | T_SU | MT_GY | Insula | O_GY | O_SU | Phip_GY | Ent_GY | Amyg | Cla | Put | Thal_MNcl | Thal_VLNcl | Sub | CA1 | CA2 | CA3 | CA4 | Cbl_cor | Cbl_Dentncl |
|---|---|---|---|---|---|---|---|---|---|---|---|---|---|---|---|---|---|---|---|---|---|---|---|
| **MF_GY** | 1.0 | 0.06 | 0.18 | 0.97 | 0.38 | 0.93 | 0.38 | 0.47 | 0.015 | 0.012 | 0.31 | 0.29 | 0.015 | 0.34 | <0.01 | 0.012 | 0.16 | 0.12 | 0.17 | 0.21 | 0.93 | <0.01 | <0.01 |
| **F_SU** | 0.06 | 1.0 | 0.8 | 0.08 | 0.45 | 0.07 | 0.53 | 0.29 | 0.6 | <0.01 | 0.017 | 0.014 | <0.01 | 0.012 | <0.01 | <0.01 | <0.01 | <0.01 | <0.01 | <0.01 | 0.06 | <0.01 | <0.01 |
| **P_GY** | 0.18 | 0.8 | 1.0 | 0.19 | 0.72 | 0.17 | 0.8 | 0.49 | 0.4 | <0.01 | 0.037 | 0.032 | <0.01 | 0.032 | <0.01 | <0.01 | <0.01 | <0.01 | 0.011 | 0.013 | 0.16 | <0.01 | <0.01 |
| **ST_GY** | 0.97 | 0.08 | 0.19 | 1.0 | 0.38 | 0.97 | 0.38 | 0.46 | 0.021 | 0.025 | 0.36 | 0.34 | 0.026 | 0.39 | <0.01 | 0.019 | 0.23 | 0.19 | 0.23 | 0.26 | 0.97 | <0.01 | <0.01 |
| **T_SU** | 0.38 | 0.45 | 0.72 | 0.38 | 1.0 | 0.35 | 0.97 | 0.87 | 0.22 | <0.01 | 0.1 | 0.09 | <0.01 | 0.09 | <0.01 | <0.01 | 0.026 | 0.02 | 0.033 | 0.039 | 0.34 | <0.01 | <0.01 |
| **MT_GY** | 0.93 | 0.07 | 0.17 | 0.97 | 0.35 | 1.0 | 0.36 | 0.42 | 0.018 | 0.031 | 0.38 | 0.37 | 0.032 | 0.42 | <0.01 | 0.022 | 0.26 | 0.22 | 0.26 | 0.29 | 1.0 | <0.01 | <0.01 |
| **Insula** | 0.38 | 0.53 | 0.8 | 0.38 | 0.97 | 0.36 | 1.0 | 0.83 | 0.28 | <0.01 | 0.1 | 0.09 | <0.01 | 0.1 | <0.01 | <0.01 | 0.032 | 0.026 | 0.038 | 0.047 | 0.35 | <0.01 | <0.01 |
| **O_GY** | 0.47 | 0.29 | 0.49 | 0.46 | 0.87 | 0.42 | 0.83 | 1.0 | 0.1 | <0.01 | 0.12 | 0.1 | <0.01 | 0.11 | <0.01 | <0.01 | 0.028 | 0.022 | 0.038 | 0.048 | 0.41 | <0.01 | <0.01 |
| **O_SU** | 0.015 | 0.6 | 0.4 | 0.021 | 0.22 | 0.018 | 0.28 | 0.1 | 1.0 | <0.01 | <0.01 | <0.01 | <0.01 | <0.01 | <0.01 | <0.01 | <0.01 | <0.01 | <0.01 | <0.01 | 0.014 | <0.01 | <0.01 |
| **Phip_GY** | 0.012 | <0.01 | <0.01 | 0.025 | <0.01 | 0.031 | <0.01 | <0.01 | <0.01 | 1.0 | 0.45 | 0.46 | 0.88 | 0.31 | 0.09 | 0.52 | 0.32 | 0.4 | 0.44 | 0.4 | 0.026 | 0.09 | 0.011 |
| **Ent_GY** | 0.31 | 0.017 | 0.037 | 0.36 | 0.1 | 0.38 | 0.1 | 0.12 | <0.01 | 0.45 | 1.0 | 0.99 | 0.38 | 0.93 | 0.036 | 0.26 | 1.0 | 0.95 | 0.96 | 0.98 | 0.38 | 0.035 | <0.01 |
| **Amyg** | 0.29 | 0.014 | 0.032 | 0.34 | 0.09 | 0.37 | 0.09 | 0.1 | <0.01 | 0.46 | 0.99 | 1.0 | 0.39 | 0.91 | 0.036 | 0.26 | 0.99 | 0.97 | 0.97 | 0.99 | 0.36 | 0.035 | <0.01 |
| **Cla** | 0.015 | <0.01 | <0.01 | 0.026 | <0.01 | 0.032 | <0.01 | <0.01 | <0.01 | 0.88 | 0.38 | 0.39 | 1.0 | 0.26 | 0.17 | 0.71 | 0.27 | 0.35 | 0.38 | 0.35 | 0.028 | 0.21 | 0.032 |
| **Put** | 0.34 | 0.012 | 0.032 | 0.39 | 0.09 | 0.42 | 0.1 | 0.11 | <0.01 | 0.31 | 0.93 | 0.91 | 0.26 | 1.0 | 0.017 | 0.17 | 0.91 | 0.8 | 0.83 | 0.88 | 0.41 | 0.011 | <0.01 |
| **Thal_MNcl** | <0.01 | <0.01 | <0.01 | <0.01 | <0.01 | <0.01 | <0.01 | <0.01 | <0.01 | 0.09 | 0.036 | 0.036 | 0.17 | 0.017 | 1.0 | 0.39 | 0.013 | 0.02 | 0.026 | 0.023 | <0.01 | 0.74 | 0.69 |
| **Thal_VLNcl** | 0.012 | <0.01 | <0.01 | 0.019 | <0.01 | 0.022 | <0.01 | <0.01 | <0.01 | 0.52 | 0.26 | 0.26 | 0.71 | 0.17 | 0.39 | 1.0 | 0.17 | 0.22 | 0.24 | 0.22 | 0.02 | 0.53 | 0.17 |
| **Sub** | 0.16 | <0.01 | <0.01 | 0.23 | 0.026 | 0.26 | 0.032 | 0.028 | <0.01 | 0.32 | 1.0 | 0.99 | 0.27 | 0.91 | 0.013 | 0.17 | 1.0 | 0.93 | 0.94 | 0.97 | 0.25 | <0.01 | <0.01 |
| **CA1** | 0.12 | <0.01 | <0.01 | 0.19 | 0.02 | 0.22 | 0.026 | 0.022 | <0.01 | 0.4 | 0.95 | 0.97 | 0.35 | 0.8 | 0.02 | 0.22 | 0.93 | 1.0 | 1.0 | 0.97 | 0.2 | 0.011 | <0.01 |
| **CA2** | 0.17 | <0.01 | 0.011 | 0.23 | 0.033 | 0.26 | 0.038 | 0.038 | <0.01 | 0.44 | 0.96 | 0.97 | 0.38 | 0.83 | 0.026 | 0.24 | 0.94 | 1.0 | 1.0 | 0.97 | 0.25 | 0.019 | <0.01 |
| **CA3** | 0.21 | <0.01 | 0.013 | 0.26 | 0.039 | 0.29 | 0.047 | 0.048 | <0.01 | 0.4 | 0.98 | 0.99 | 0.35 | 0.88 | 0.023 | 0.22 | 0.97 | 0.97 | 0.97 | 1.0 | 0.28 | 0.017 | <0.01 |
| **CA4** | 0.93 | 0.06 | 0.16 | 0.97 | 0.34 | 1.0 | 0.35 | 0.41 | 0.014 | 0.026 | 0.38 | 0.36 | 0.028 | 0.41 | <0.01 | 0.02 | 0.25 | 0.2 | 0.25 | 0.28 | 1.0 | <0.01 | <0.01 |
| **Cbl_cor** | <0.01 | <0.01 | <0.01 | <0.01 | <0.01 | <0.01 | <0.01 | <0.01 | <0.01 | 0.09 | 0.035 | 0.035 | 0.21 | 0.011 | 0.74 | 0.53 | <0.01 | 0.011 | 0.019 | 0.017 | <0.01 | 1.0 | 0.34 |
| **Cbl_Dentncl** | <0.01 | <0.01 | <0.01 | <0.01 | <0.01 | <0.01 | <0.01 | <0.01 | <0.01 | 0.011 | <0.01 | <0.01 | 0.032 | <0.01 | 0.69 | 0.17 | <0.01 | <0.01 | <0.01 | <0.01 | <0.01 | 0.34 | 1.0 |

Amyg amygdala, Cbl cerebellum, Cla claustrum, cor cortex, Dentncl dentate nucleus, Ent entorhinal, F frontal, GY gyrus, MF middle frontal, MNcl medial nucleus, MT middle temporal, O occipital, P parietal, Phip parahippocampal, Put putamen, ST superior temporal, SU sulcus, Sub subiculum, T temporal, Thal thalamus, VlNcl ventrolateral nucleus

***Table S9:*** *Comparison of proportional loads of cerebral amyloid angiopathy (CAA) across brain regions [%]*
*Kruskal-Wallis tests and Dunn's post hoc analysis with FDR Benjamini-Hochberg correction for multiple testing. Regions are indicated with background colors, significant, false discovery rate corrected p-values are labeled in green font color.*

| | MF_GY | F_SU | P_GY | ST_GY | T_SU | MT_GY | Insula | O_GY | O_SU | Phip_GY | Ent_GY | Amyg | Cla | Put | Thal_MNcl | Thal_VLNcl | Sub | CA1 | CA2 | CA3 | CA4 | Cbl_cor | Cbl_Dentncl |
|---|---|---|---|---|---|---|---|---|---|---|---|---|---|---|---|---|---|---|---|---|---|---|---|
| **MF_GY** | 1.0 | 0.045 | 0.9 | 0.9 | 0.85 | 0.32 | 0.51 | 0.16 | 0.11 | 0.81 | 0.4 | 0.39 | 0.25 | 0.4 | 0.46 | 0.24 | 0.78 | 0.95 | 0.24 | 0.75 | 0.75 | 0.19 | 0.17 |
| **F_SU** | 0.045 | 1.0 | 0.1 | 0.045 | 0.13 | 0.47 | 0.017 | 0.63 | 0.77 | 0.11 | 0.019 | 0.017 | <0.01 | 0.015 | 0.028 | 0.012 | 0.019 | 0.047 | <0.01 | 0.031 | 0.15 | 0.66 | <0.01 |
| **P_GY** | 0.9 | 0.1 | 1.0 | 0.83 | 0.93 | 0.44 | 0.44 | 0.27 | 0.19 | 0.91 | 0.35 | 0.34 | 0.21 | 0.35 | 0.4 | 0.2 | 0.69 | 0.87 | 0.21 | 0.65 | 0.86 | 0.3 | 0.15 |
| **ST_GY** | 0.9 | 0.045 | 0.83 | 1.0 | 0.77 | 0.3 | 0.65 | 0.15 | 0.11 | 0.72 | 0.52 | 0.5 | 0.34 | 0.53 | 0.56 | 0.31 | 0.9 | 0.94 | 0.34 | 0.85 | 0.65 | 0.18 | 0.26 |
| **T_SU** | 0.85 | 0.13 | 0.93 | 0.77 | 1.0 | 0.52 | 0.4 | 0.32 | 0.25 | 0.97 | 0.32 | 0.31 | 0.19 | 0.32 | 0.36 | 0.18 | 0.62 | 0.82 | 0.18 | 0.58 | 0.92 | 0.35 | 0.13 |
| **MT_GY** | 0.32 | 0.47 | 0.44 | 0.3 | 0.52 | 1.0 | 0.12 | 0.85 | 0.75 | 0.52 | 0.11 | 0.1 | 0.039 | 0.1 | 0.13 | 0.047 | 0.19 | 0.31 | 0.039 | 0.2 | 0.61 | 0.85 | 0.028 |
| **Insula** | 0.51 | 0.017 | 0.44 | 0.65 | 0.4 | 0.12 | 1.0 | 0.049 | 0.037 | 0.34 | 0.86 | 0.85 | 0.75 | 0.91 | 0.9 | 0.63 | 0.75 | 0.58 | 0.75 | 0.85 | 0.31 | 0.06 | 0.58 |
| **O_GY** | 0.16 | 0.63 | 0.27 | 0.15 | 0.32 | 0.85 | 0.049 | 1.0 | 0.9 | 0.31 | 0.05 | 0.047 | 0.017 | 0.039 | 0.07 | 0.024 | 0.08 | 0.16 | 0.017 | 0.1 | 0.38 | 1.0 | 0.014 |
| **O_SU** | 0.11 | 0.77 | 0.19 | 0.11 | 0.25 | 0.75 | 0.037 | 0.9 | 1.0 | 0.24 | 0.039 | 0.037 | 0.013 | 0.028 | 0.049 | 0.017 | 0.047 | 0.11 | 0.012 | 0.07 | 0.3 | 0.9 | 0.012 |
| **Phip_GY** | 0.81 | 0.11 | 0.91 | 0.72 | 0.97 | 0.52 | 0.34 | 0.31 | 0.24 | 1.0 | 0.3 | 0.28 | 0.15 | 0.29 | 0.33 | 0.15 | 0.54 | 0.77 | 0.15 | 0.52 | 0.93 | 0.34 | 0.11 |
| **Ent_GY** | 0.4 | 0.019 | 0.35 | 0.52 | 0.32 | 0.11 | 0.86 | 0.05 | 0.039 | 0.3 | 1.0 | 0.99 | 0.92 | 0.93 | 0.98 | 0.83 | 0.6 | 0.46 | 0.92 | 0.71 | 0.27 | 0.06 | 0.81 |
| **Amyg** | 0.39 | 0.017 | 0.34 | 0.5 | 0.31 | 0.1 | 0.85 | 0.047 | 0.037 | 0.28 | 0.99 | 1.0 | 0.92 | 0.92 | 0.97 | 0.84 | 0.58 | 0.44 | 0.92 | 0.69 | 0.25 | 0.05 | 0.82 |
| **Cla** | 0.25 | <0.01 | 0.21 | 0.34 | 0.19 | 0.039 | 0.75 | 0.017 | 0.013 | 0.15 | 0.92 | 0.92 | 1.0 | 0.85 | 0.9 | 0.88 | 0.4 | 0.3 | 1.0 | 0.53 | 0.13 | 0.019 | 0.87 |
| **Put** | 0.4 | 0.015 | 0.35 | 0.53 | 0.32 | 0.1 | 0.91 | 0.039 | 0.028 | 0.29 | 0.93 | 0.92 | 0.85 | 1.0 | 0.97 | 0.75 | 0.63 | 0.47 | 0.85 | 0.75 | 0.26 | 0.047 | 0.71 |
| **Thal_MNcl** | 0.46 | 0.028 | 0.4 | 0.56 | 0.36 | 0.13 | 0.9 | 0.07 | 0.049 | 0.33 | 0.98 | 0.97 | 0.9 | 0.97 | 1.0 | 0.81 | 0.65 | 0.51 | 0.9 | 0.75 | 0.3 | 0.08 | 0.79 |
| **Thal_VLNcl** | 0.24 | 0.012 | 0.2 | 0.31 | 0.18 | 0.047 | 0.63 | 0.024 | 0.017 | 0.15 | 0.83 | 0.84 | 0.88 | 0.75 | 0.81 | 1.0 | 0.35 | 0.28 | 0.87 | 0.46 | 0.14 | 0.028 | 1.0 |
| **Sub** | 0.78 | 0.019 | 0.69 | 0.9 | 0.62 | 0.19 | 0.75 | 0.08 | 0.047 | 0.54 | 0.6 | 0.58 | 0.4 | 0.63 | 0.65 | 0.35 | 1.0 | 0.85 | 0.4 | 0.92 | 0.49 | 0.1 | 0.3 |
| **CA1** | 0.95 | 0.047 | 0.87 | 0.94 | 0.82 | 0.31 | 0.58 | 0.16 | 0.11 | 0.77 | 0.46 | 0.44 | 0.3 | 0.47 | 0.51 | 0.28 | 0.85 | 1.0 | 0.3 | 0.79 | 0.71 | 0.19 | 0.21 |
| **CA2** | 0.24 | <0.01 | 0.21 | 0.34 | 0.18 | 0.039 | 0.75 | 0.017 | 0.012 | 0.15 | 0.92 | 0.92 | 1.0 | 0.85 | 0.9 | 0.87 | 0.4 | 0.3 | 1.0 | 0.53 | 0.13 | 0.019 | 0.86 |
| **CA3** | 0.75 | 0.031 | 0.65 | 0.85 | 0.58 | 0.2 | 0.85 | 0.1 | 0.07 | 0.52 | 0.71 | 0.69 | 0.53 | 0.75 | 0.75 | 0.46 | 0.92 | 0.79 | 0.53 | 1.0 | 0.47 | 0.12 | 0.4 |
| **CA4** | 0.75 | 0.15 | 0.86 | 0.65 | 0.92 | 0.61 | 0.31 | 0.38 | 0.3 | 0.93 | 0.27 | 0.25 | 0.13 | 0.26 | 0.3 | 0.14 | 0.49 | 0.71 | 0.13 | 0.47 | 1.0 | 0.41 | 0.1 |
| **Cbl_cor** | 0.19 | 0.66 | 0.3 | 0.18 | 0.35 | 0.85 | 0.06 | 1.0 | 0.9 | 0.34 | 0.06 | 0.05 | 0.019 | 0.047 | 0.08 | 0.028 | 0.1 | 0.19 | 0.019 | 0.12 | 0.41 | 1.0 | 0.017 |
| **Cbl_Dentncl** | 0.17 | <0.01 | 0.15 | 0.26 | 0.13 | 0.028 | 0.58 | 0.014 | 0.012 | 0.11 | 0.81 | 0.82 | 0.87 | 0.71 | 0.79 | 1.0 | 0.3 | 0.21 | 0.86 | 0.4 | 0.1 | 0.017 | 1.0 |

Amyg amygdala, Cbl cerebellum, Cla claustrum, cor cortex, Dentncl dentate nucleus, Ent entorhinal, F frontal, GY gyrus, MF middle frontal, MNcl medial nucleus, MT middle temporal, O occipital, P parietal, Phip parahippocampal, Put putamen, ST superior temporal, SU sulcus, Sub subiculum, T temporal, Thal thalamus, VlNcl ventrolateral nucleus

***Table S10:*** *Comparison of proportional loads of small dense plaques across brain regions [%]*
*Kruskal-Wallis tests and Dunn’s post hoc analysis with FDR Benjamini-Hochberg correction for multiple testing. Regions are indicated with background colors, significant, false discovery rate corrected p-values are labeled in green font color.*

| | MF_GY | F_SU | P_GY | ST_GY | T_SU | MT_GY | Insula | O_GY | O_SU | Phip_GY | Ent_GY | Amyg | Cla | Put | Thal_MNcl | Thal_VLNcl | Sub | CA1 | CA2 | CA3 | CA4 | Cbl_cor | Cbl_Dentncl |
|---|---|---|---|---|---|---|---|---|---|---|---|---|---|---|---|---|---|---|---|---|---|---|---|
| **MF_GY** | 1.0 | 0.72 | 0.41 | 0.84 | 0.32 | 0.23 | 0.92 | 0.06 | 0.13 | 0.016 | <0.01 | <0.01 | 0.016 | 0.09 | 0.09 | 0.89 | 0.036 | 0.42 | 0.69 | 0.76 | 0.68 | 0.47 | 0.13 |
| **F_SU** | 0.72 | 1.0 | 0.66 | 0.6 | 0.17 | 0.11 | 0.7 | 0.019 | 0.06 | <0.01 | <0.01 | <0.01 | <0.01 | 0.17 | 0.047 | 0.89 | 0.011 | 0.23 | 0.92 | 0.54 | 0.43 | 0.27 | 0.23 |
| **P_GY** | 0.41 | 0.66 | 1.0 | 0.33 | 0.08 | 0.049 | 0.43 | <0.01 | 0.019 | <0.01 | <0.01 | <0.01 | <0.01 | 0.39 | 0.019 | 0.66 | <0.01 | 0.11 | 0.75 | 0.3 | 0.22 | 0.13 | 0.48 |
| **ST_GY** | 0.84 | 0.6 | 0.33 | 1.0 | 0.48 | 0.36 | 0.92 | 0.13 | 0.26 | 0.05 | <0.01 | <0.01 | 0.042 | 0.07 | 0.14 | 0.78 | 0.09 | 0.6 | 0.59 | 0.9 | 0.84 | 0.65 | 0.1 |
| **T_SU** | 0.32 | 0.17 | 0.08 | 0.48 | 1.0 | 0.85 | 0.46 | 0.52 | 0.75 | 0.28 | <0.01 | 0.02 | 0.21 | 0.011 | 0.41 | 0.4 | 0.41 | 0.84 | 0.2 | 0.6 | 0.61 | 0.82 | 0.019 |
| **MT_GY** | 0.23 | 0.11 | 0.049 | 0.36 | 0.85 | 1.0 | 0.35 | 0.66 | 0.89 | 0.37 | <0.01 | 0.033 | 0.28 | <0.01 | 0.5 | 0.31 | 0.54 | 0.72 | 0.14 | 0.47 | 0.48 | 0.68 | 0.01 |
| **Insula** | 0.92 | 0.7 | 0.43 | 0.92 | 0.46 | 0.35 | 1.0 | 0.14 | 0.26 | 0.06 | <0.01 | <0.01 | 0.047 | 0.11 | 0.14 | 0.84 | 0.1 | 0.56 | 0.68 | 0.84 | 0.78 | 0.6 | 0.15 |
| **O_GY** | 0.06 | 0.019 | <0.01 | 0.13 | 0.52 | 0.66 | 0.14 | 1.0 | 0.73 | 0.66 | 0.018 | 0.07 | 0.49 | <0.01 | 0.73 | 0.14 | 0.84 | 0.36 | 0.036 | 0.21 | 0.19 | 0.34 | <0.01 |
| **O_SU** | 0.13 | 0.06 | 0.019 | 0.26 | 0.75 | 0.89 | 0.26 | 0.73 | 1.0 | 0.41 | <0.01 | 0.033 | 0.3 | <0.01 | 0.55 | 0.24 | 0.6 | 0.6 | 0.08 | 0.36 | 0.36 | 0.56 | <0.01 |
| **Phip_GY** | 0.016 | <0.01 | <0.01 | 0.05 | 0.28 | 0.37 | 0.06 | 0.66 | 0.41 | 1.0 | 0.06 | 0.16 | 0.78 | <0.01 | 0.96 | 0.07 | 0.79 | 0.16 | 0.011 | 0.09 | 0.08 | 0.15 | <0.01 |
| **Ent_GY** | <0.01 | <0.01 | <0.01 | <0.01 | <0.01 | <0.01 | <0.01 | 0.018 | <0.01 | 0.06 | 1.0 | 0.7 | 0.13 | <0.01 | 0.13 | <0.01 | 0.03 | <0.01 | <0.01 | <0.01 | <0.01 | <0.01 | <0.01 |
| **Amyg** | <0.01 | <0.01 | <0.01 | <0.01 | 0.02 | 0.033 | <0.01 | 0.07 | 0.033 | 0.16 | 0.7 | 1.0 | 0.3 | <0.01 | 0.28 | <0.01 | 0.1 | 0.01 | <0.01 | <0.01 | <0.01 | 0.01 | <0.01 |
| **Cla** | 0.016 | <0.01 | <0.01 | 0.042 | 0.21 | 0.28 | 0.047 | 0.49 | 0.3 | 0.78 | 0.13 | 0.3 | 1.0 | <0.01 | 0.84 | 0.05 | 0.61 | 0.13 | 0.01 | 0.07 | 0.06 | 0.12 | <0.01 |
| **Put** | 0.09 | 0.17 | 0.39 | 0.07 | 0.011 | <0.01 | 0.11 | <0.01 | <0.01 | <0.01 | <0.01 | <0.01 | <0.01 | 1.0 | <0.01 | 0.25 | <0.01 | 0.015 | 0.26 | 0.07 | 0.04 | 0.019 | 0.91 |
| **Thal_MNcl** | 0.09 | 0.047 | 0.019 | 0.14 | 0.41 | 0.5 | 0.14 | 0.73 | 0.55 | 0.96 | 0.13 | 0.28 | 0.84 | <0.01 | 1.0 | 0.13 | 0.83 | 0.3 | 0.06 | 0.19 | 0.19 | 0.29 | <0.01 |
| **Thal_VLNcl** | 0.89 | 0.89 | 0.66 | 0.78 | 0.4 | 0.31 | 0.84 | 0.14 | 0.24 | 0.07 | <0.01 | <0.01 | 0.05 | 0.25 | 0.13 | 1.0 | 0.11 | 0.48 | 0.84 | 0.73 | 0.67 | 0.52 | 0.3 |
| **Sub** | 0.036 | 0.011 | <0.01 | 0.09 | 0.41 | 0.54 | 0.1 | 0.84 | 0.6 | 0.79 | 0.03 | 0.1 | 0.61 | <0.01 | 0.83 | 0.11 | 1.0 | 0.27 | 0.023 | 0.15 | 0.14 | 0.26 | <0.01 |
| **CA1** | 0.42 | 0.23 | 0.11 | 0.6 | 0.84 | 0.72 | 0.56 | 0.36 | 0.6 | 0.16 | <0.01 | 0.01 | 0.13 | 0.015 | 0.3 | 0.48 | 0.27 | 1.0 | 0.26 | 0.71 | 0.74 | 0.95 | 0.025 |
| **CA2** | 0.69 | 0.92 | 0.75 | 0.59 | 0.2 | 0.14 | 0.68 | 0.036 | 0.08 | 0.011 | <0.01 | <0.01 | 0.01 | 0.26 | 0.06 | 0.84 | 0.023 | 0.26 | 1.0 | 0.53 | 0.44 | 0.29 | 0.32 |
| **CA3** | 0.76 | 0.54 | 0.3 | 0.9 | 0.6 | 0.47 | 0.84 | 0.21 | 0.36 | 0.09 | <0.01 | <0.01 | 0.07 | 0.07 | 0.19 | 0.73 | 0.15 | 0.71 | 0.53 | 1.0 | 0.93 | 0.74 | 0.1 |
| **CA4** | 0.68 | 0.43 | 0.22 | 0.84 | 0.61 | 0.48 | 0.78 | 0.19 | 0.36 | 0.08 | <0.01 | <0.01 | 0.06 | 0.04 | 0.19 | 0.67 | 0.14 | 0.74 | 0.44 | 0.93 | 1.0 | 0.78 | 0.06 |
| **Cbl_cor** | 0.47 | 0.27 | 0.13 | 0.65 | 0.82 | 0.68 | 0.6 | 0.34 | 0.56 | 0.15 | <0.01 | 0.01 | 0.12 | 0.019 | 0.29 | 0.52 | 0.26 | 0.95 | 0.29 | 0.74 | 0.78 | 1.0 | 0.032 |
| **Cbl_Dentncl** | 0.13 | 0.23 | 0.48 | 0.1 | 0.019 | 0.01 | 0.15 | <0.01 | <0.01 | <0.01 | <0.01 | <0.01 | <0.01 | 0.91 | <0.01 | 0.3 | <0.01 | 0.025 | 0.32 | 0.1 | 0.06 | 0.032 | 1.0 |

Amyg amygdala, Cbl cerebellum, Cla claustrum, cor cortex, Dentncl dentate nucleus, Ent entorhinal, F frontal, GY gyrus, MF middle frontal, MNcl medial nucleus, MT middle temporal, O occipital, P parietal, Phip parahippocampal, Put putamen, ST superior temporal, SU sulcus, Sub subiculum, T temporal, Thal thalamus, VlNcl ventrolateral nucleus

**Table S11:** *Comparison of total Aβ deposit counts/mm² between brain regions in a complete subset*
*Complete subset is defined as all cases with completed Aβ staining in the listed nine brain regions (n=40). (a) Kruskal-Wallis tests and Dunn's post hoc analysis and (b) Wilcoxon signed-rank test with FDR Benjamini-Hochberg correction for multiple testing, respectively. Regions are indicated with background colors, significant, false discovery rate corrected p-values are labeled in green font color.*

| a) | MF_GY | F_SU | O_GY | O_SU | Phip_GY | Sub | CA1 | Cbl_cor | Cbl_Dentncl |
|---|---|---|---|---|---|---|---|---|---|
| MF_GY | 1.0 | 0.44 | 0.013 | 0.06 | <0.01 | <0.01 | <0.01 | <0.01 | <0.01 |
| F_SU | 0.44 | 1.0 | <0.01 | <0.01 | <0.01 | <0.01 | <0.01 | <0.01 | <0.01 |
| O_GY | 0.013 | <0.01 | 1.0 | 0.55 | 0.63 | 0.24 | <0.01 | <0.01 | <0.01 |
| O_SU | 0.06 | <0.01 | 0.55 | 1.0 | 0.31 | 0.08 | <0.01 | <0.01 | <0.01 |
| Phip_GY | <0.01 | <0.01 | 0.63 | 0.31 | 1.0 | 0.45 | <0.01 | <0.01 | <0.01 |
| Sub | <0.01 | <0.01 | 0.24 | 0.08 | 0.45 | 1.0 | 0.032 | <0.01 | <0.01 |
| CA1 | <0.01 | <0.01 | <0.01 | <0.01 | <0.01 | 0.032 | 1.0 | 0.13 | 0.012 |
| Cbl_cor | <0.01 | <0.01 | <0.01 | <0.01 | <0.01 | <0.01 | 0.13 | 1.0 | 0.36 |
| Cbl_Dentncl | <0.01 | <0.01 | <0.01 | <0.01 | <0.01 | <0.01 | 0.012 | 0.36 | 1.0 |

| b) | MF_GY | F_SU | O_GY | O_SU | Phip_GY | Sub | CA1 | Cbl_cor | Cbl_Dentncl |
|---|---|---|---|---|---|---|---|---|---|
| MF_GY | 1.00 | 0.037 | <0.01 | <0.01 | <0.01 | <0.01 | <0.01 | <0.01 | <0.01 |
| F_SU | 0.037 | 1.00 | <0.01 | <0.01 | <0.01 | <0.01 | <0.01 | <0.01 | <0.01 |
| O_GY | <0.01 | <0.01 | 1.00 | 0.33 | 0.48 | 0.36 | <0.01 | <0.01 | <0.01 |
| O_SU | <0.01 | <0.01 | 0.33 | 1.00 | 0.08 | 0.016 | <0.01 | <0.01 | <0.01 |
| Phip_GY | <0.01 | <0.01 | 0.48 | 0.08 | 1.00 | 0.23 | <0.01 | <0.01 | <0.01 |
| Sub | <0.01 | <0.01 | 0.36 | 0.016 | 0.23 | 1.00 | <0.01 | <0.01 | <0.01 |
| CA1 | <0.01 | <0.01 | <0.01 | <0.01 | <0.01 | <0.01 | 1.00 | 0.011 | <0.01 |
| Cbl_cor | <0.01 | <0.01 | <0.01 | <0.01 | <0.01 | <0.01 | 0.011 | 1.00 | 0.037 |
| Cbl_Dentncl | <0.01 | <0.01 | <0.01 | <0.01 | <0.01 | <0.01 | <0.01 | 0.037 | 1.00 |

Cbl cerebellum, cor cortex, Dentncl dentate nucleus, F frontal, GY gyrus, MF middle frontal, O occipital, Phip parahippocampal, SU sulcus, Sub subiculum

***Table S12:*** *Comparison of proportional loads of cored plaques across brain regions [%] in a complete subset*
*Complete subset is defined as all cases with completed Aβ staining in the listed nine brain regions.* (a) *Kruskal-Wallis tests and Dunn's post hoc analysis and (b) Wilcoxon signed-rank test with FDR Benjamini-Hochberg correction for multiple testing, respectively. Regions are indicated with background colors, significant, false discovery rate corrected p-values are labeled in green font color.*

| a) | MF_GY | F_SU | O_GY | O_SU | Phip_GY | Sub | CA1 | Cbl_cor | Cbl_Dentncl |
|---|---|---|---|---|---|---|---|---|---|
| **MF_GY** | 1.0 | 0.44 | 0.82 | 0.49 | <0.01 | 0.043 | <0.01 | <0.01 | <0.01 |
| **F_SU** | 0.44 | 1.0 | 0.32 | 0.13 | <0.01 | <0.01 | <0.01 | <0.01 | <0.01 |
| **O_GY** | 0.82 | 0.32 | 1.0 | 0.61 | 0.016 | 0.07 | 0.013 | <0.01 | <0.01 |
| **O_SU** | 0.49 | 0.13 | 0.61 | 1.0 | 0.06 | 0.19 | 0.048 | <0.01 | <0.01 |
| **Phip_GY** | <0.01 | <0.01 | 0.016 | 0.06 | 1.0 | 0.58 | 0.86 | 0.29 | 0.13 |
| **Sub** | 0.043 | <0.01 | 0.07 | 0.19 | 0.58 | 1.0 | 0.49 | 0.11 | 0.048 |
| **CA1** | <0.01 | <0.01 | 0.013 | 0.048 | 0.86 | 0.49 | 1.0 | 0.38 | 0.18 |
| **Cbl_cor** | <0.01 | <0.01 | <0.01 | <0.01 | 0.29 | 0.11 | 0.38 | 1.0 | 0.61 |
| **Cbl_Dentncl** | <0.01 | <0.01 | <0.01 | <0.01 | 0.13 | 0.048 | 0.18 | 0.61 | 1.0 |

| b) | MF_GY | F_SU | O_GY | O_SU | Phip_GY | Sub | CA1 | Cbl_cor | Cbl_Dentncl |
|---|---|---|---|---|---|---|---|---|---|
| **MF_GY** | 1.00 | 0.41 | 0.82 | 0.79 | 0.032 | 0.58 | 0.35 | 0.032 | 0.030 |
| **F_SU** | 0.41 | 1.00 | 0.59 | 0.39 | 0.015 | 0.16 | 0.35 | 0.032 | 0.018 |
| **O_GY** | 0.82 | 0.59 | 1.00 | 0.58 | 0.032 | 0.39 | 0.36 | 0.06 | 0.030 |
| **O_SU** | 0.79 | 0.39 | 0.58 | 1.00 | 0.06 | 0.82 | 0.91 | 0.07 | 0.08 |
| **Phip_GY** | 0.032 | 0.015 | 0.032 | 0.06 | 1.00 | 0.29 | 0.74 | 0.80 | 0.16 |
| **Sub** | 0.58 | 0.16 | 0.39 | 0.82 | 0.29 | 1.00 | 0.78 | 0.39 | 0.16 |
| **CA1** | 0.35 | 0.35 | 0.36 | 0.91 | 0.74 | 0.78 | 1.00 | 0.80 | 0.24 |
| **Cbl_cor** | 0.032 | 0.032 | 0.06 | 0.07 | 0.80 | 0.39 | 0.80 | 1.00 | 0.35 |
| **Cbl_Dentncl** | 0.030 | 0.018 | 0.030 | 0.08 | 0.16 | 0.16 | 0.24 | 0.35 | 1.00 |

Cbl cerebellum, cor cortex, Dentncl dentate nucleus, F frontal, GY gyrus, MF middle frontal, O occipital, Phip parahippocampal, SU sulcus, Sub subiculum

***Table S13:*** *Comparison of proportional loads of diffuse light plaques across brain regions [%] in complete subset*
*Complete subset is defined as all cases with completed Aβ staining in the listed nine brain regions. (a) Kruskal-Wallis tests and Dunn's post hoc analysis and (b) Wilcoxon signed-rank test with FDR Benjamini-Hochberg correction for multiple testing, respectively. Regions are indicated with background colors, significant, false discovery rate corrected p-values are labeled in green font color.*

| a) | MF_GY | F_SU | O_GY | O_SU | Phip_GY | Sub | CA1 | Cbl_cor | Cbl_Dentncl |
|---|---|---|---|---|---|---|---|---|---|
| **MF_GY** | 1.0 | 0.21 | 0.77 | 0.43 | 0.13 | 0.61 | 0.7 | 0.73 | 0.7 |
| **F_SU** | 0.21 | 1.0 | 0.12 | 0.7 | <0.01 | 0.07 | 0.43 | 0.43 | 0.6 |
| **O_GY** | 0.77 | 0.12 | 1.0 | 0.33 | 0.24 | 0.73 | 0.6 | 0.61 | 0.6 |
| **O_SU** | 0.43 | 0.7 | 0.33 | 1.0 | 0.013 | 0.15 | 0.7 | 0.7 | 0.77 |
| **Phip_GY** | 0.13 | <0.01 | 0.24 | 0.013 | 1.0 | 0.43 | 0.07 | 0.1 | 0.11 |
| **Sub** | 0.61 | 0.07 | 0.73 | 0.15 | 0.43 | 1.0 | 0.43 | 0.43 | 0.43 |
| **CA1** | 0.7 | 0.43 | 0.6 | 0.7 | 0.07 | 0.43 | 1.0 | 0.95 | 0.95 |
| **Cbl_cor** | 0.73 | 0.43 | 0.61 | 0.7 | 0.1 | 0.43 | 0.95 | 1.0 | 0.94 |
| **Cbl_Dentncl** | 0.7 | 0.6 | 0.6 | 0.77 | 0.11 | 0.43 | 0.95 | 0.94 | 1.0 |

| b) | MF_GY | F_SU | O_GY | O_SU | Phip_GY | Sub | CA1 | Cbl_cor | Cbl_Dentncl |
|---|---|---|---|---|---|---|---|---|---|
| **MF_GY** | 1.00 | 0.011 | 0.82 | 0.35 | <0.01 | 0.49 | 1.00 | 0.89 | 0.89 |
| **F_SU** | 0.011 | 1.00 | 0.028 | 0.63 | <0.01 | 0.018 | 0.58 | 0.53 | 0.65 |
| **O_GY** | 0.82 | 0.028 | 1.00 | 0.06 | 0.20 | 0.85 | 0.53 | 0.82 | 0.94 |
| **O_SU** | 0.35 | 0.63 | 0.06 | 1.00 | <0.01 | 0.049 | 0.65 | 0.91 | 0.82 |
| **Phip_GY** | <0.01 | <0.01 | 0.20 | <0.01 | 1.00 | 0.40 | <0.01 | 0.20 | 0.47 |
| **Sub** | 0.49 | 0.018 | 0.85 | 0.049 | 0.40 | 1.00 | 0.35 | 0.89 | 0.94 |
| **CA1** | 1.00 | 0.58 | 0.53 | 0.65 | <0.01 | 0.35 | 1.00 | 0.99 | 0.65 |
| **Cbl_cor** | 0.89 | 0.53 | 0.82 | 0.91 | 0.20 | 0.89 | 0.99 | 1.00 | 1.00 |
| **Cbl_Dentncl** | 0.89 | 0.65 | 0.94 | 0.82 | 0.47 | 0.94 | 0.65 | 1.00 | 1.00 |

Cbl cerebellum, cor cortex, Dentncl dentate nucleus, F frontal, GY gyrus, MF middle frontal, O occipital, Phip parahippocampal, SU sulcus, Sub subiculum

***Table S14:*** *Comparison of proportional loads of diffuse dense plaques across brain regions [%] in complete subset*
*Complete subset is defined as all cases with completed Aβ staining in the listed nine brain regions. (a) Kruskal-Wallis tests and Dunn's post hoc analysis and (b) Wilcoxon signed-rank test with FDR Benjamini-Hochberg correction for multiple testing, respectively. Regions are indicated with background colors, significant, false discovery rate corrected p-values are labeled in green font color.*

| a) | MF_GY | F_SU | O_GY | O_SU | Phip_GY | Sub | CA1 | Cbl_cor | Cbl_Dentncl |
|---|---|---|---|---|---|---|---|---|---|
| **MF_GY** | 1.0 | 0.05 | 0.79 | 0.21 | 0.35 | 0.79 | 0.36 | 0.79 | <0.01 |
| **F_SU** | 0.05 | 1.0 | 0.12 | 0.62 | <0.01 | 0.12 | 0.42 | 0.18 | <0.01 |
| **O_GY** | 0.79 | 0.12 | 1.0 | 0.33 | 0.22 | 0.99 | 0.53 | 0.99 | <0.01 |
| **O_SU** | 0.21 | 0.62 | 0.33 | 1.0 | 0.015 | 0.33 | 0.79 | 0.36 | <0.01 |
| **Phip_GY** | 0.35 | <0.01 | 0.22 | 0.015 | 1.0 | 0.22 | 0.05 | 0.27 | 0.018 |
| **Sub** | 0.79 | 0.12 | 0.99 | 0.33 | 0.22 | 1.0 | 0.53 | 0.99 | <0.01 |
| **CA1** | 0.36 | 0.42 | 0.53 | 0.79 | 0.05 | 0.53 | 1.0 | 0.58 | <0.01 |
| **Cbl_cor** | 0.79 | 0.18 | 0.99 | 0.36 | 0.27 | 0.99 | 0.58 | 1.0 | <0.01 |
| **Cbl_Dentncl** | <0.01 | <0.01 | <0.01 | <0.01 | 0.018 | <0.01 | <0.01 | <0.01 | 1.0 |

| b) | MF_GY | F_SU | O_GY | O_SU | Phip_GY | Sub | CA1 | Cbl_cor | Cbl_Dentncl |
|---|---|---|---|---|---|---|---|---|---|
| **MF_GY** | 1.00 | <0.01 | 0.85 | 0.10 | 0.14 | 0.47 | 0.38 | 0.85 | <0.01 |
| **F_SU** | <0.01 | 1.00 | 0.05 | 0.39 | <0.01 | 0.15 | 0.53 | 0.27 | <0.01 |
| **O_GY** | 0.85 | 0.05 | 1.00 | 0.17 | 0.15 | 0.53 | 0.53 | 0.95 | <0.01 |
| **O_SU** | 0.10 | 0.39 | 0.17 | 1.00 | <0.01 | 0.39 | 0.95 | 0.90 | 0.017 |
| **Phip_GY** | 0.14 | <0.01 | 0.15 | <0.01 | 1.00 | 0.035 | 0.027 | 0.16 | 0.037 |
| **Sub** | 0.47 | 0.15 | 0.53 | 0.39 | 0.035 | 1.00 | 0.68 | 0.92 | 0.037 |
| **CA1** | 0.38 | 0.53 | 0.53 | 0.95 | 0.027 | 0.68 | 1.00 | 0.99 | 0.029 |
| **Cbl_cor** | 0.85 | 0.27 | 0.95 | 0.90 | 0.16 | 0.92 | 0.99 | 1.00 | 0.10 |
| **Cbl_Dentncl** | <0.01 | <0.01 | <0.01 | 0.017 | 0.037 | 0.037 | 0.029 | 0.10 | 1.00 |

Cbl cerebellum, cor cortex, Dentncl dentate nucleus, F frontal, GY gyrus, MF middle frontal, O occipital, Phip parahippocampal, SU sulcus, Sub subiculum

***Table S15:*** *Comparison of proportional loads of compact plaques across brain regions [%] in complete subset*
*Complete subset is defined as all cases with completed Aβ staining in the listed nine brain regions. (a) Kruskal-Wallis tests and Dunn's post hoc analysis and (b) Wilcoxon signed-rank test with FDR Benjamini-Hochberg correction for multiple testing, respectively. Regions are indicated with background colors, significant, false discovery rate corrected p-values are labeled in green font color.*

| a) | MF_GY | F_SU | O_GY | O_SU | Phip_GY | Sub | CA1 | Cbl_cor | Cbl_Dentncl |
|---|---|---|---|---|---|---|---|---|---|
| MF_GY | 1.0 | 0.16 | 0.96 | 0.18 | 0.029 | 0.026 | 0.038 | <0.01 | <0.01 |
| F_SU | 0.16 | 1.0 | 0.14 | 0.96 | <0.01 | <0.01 | <0.01 | <0.01 | <0.01 |
| O_GY | 0.96 | 0.14 | 1.0 | 0.17 | 0.034 | 0.029 | 0.044 | <0.01 | <0.01 |
| O_SU | 0.18 | 0.96 | 0.17 | 1.0 | <0.01 | <0.01 | <0.01 | <0.01 | <0.01 |
| Phip_GY | 0.029 | <0.01 | 0.034 | <0.01 | 1.0 | 0.96 | 0.96 | 0.08 | 0.014 |
| Sub | 0.026 | <0.01 | 0.029 | <0.01 | 0.96 | 1.0 | 0.96 | 0.09 | 0.014 |
| CA1 | 0.038 | <0.01 | 0.044 | <0.01 | 0.96 | 0.96 | 1.0 | 0.08 | 0.014 |
| Cbl_cor | <0.01 | <0.01 | <0.01 | <0.01 | 0.08 | 0.09 | 0.08 | 1.0 | 0.38 |
| Cbl_Dentncl | <0.01 | <0.01 | <0.01 | <0.01 | 0.014 | 0.014 | 0.014 | 0.38 | 1.0 |

| b) | MF_GY | F_SU | O_GY | O_SU | Phip_GY | Sub | CA1 | Cbl_cor | Cbl_Dentncl |
|---|---|---|---|---|---|---|---|---|---|
| MF_GY | 1.00 | <0.01 | 0.82 | 0.022 | <0.01 | 0.06 | 0.47 | 0.031 | <0.01 |
| F_SU | <0.01 | 1.00 | 0.25 | 0.47 | <0.01 | <0.01 | 0.10 | <0.01 | <0.01 |
| O_GY | 0.82 | 0.25 | 1.00 | 0.028 | <0.01 | 0.10 | 0.33 | 0.07 | <0.01 |
| O_SU | 0.022 | 0.47 | 0.028 | 1.00 | <0.01 | <0.01 | 0.019 | <0.01 | <0.01 |
| Phip_GY | <0.01 | <0.01 | <0.01 | <0.01 | 1.00 | 0.74 | 0.38 | 0.11 | 0.05 |
| Sub | 0.06 | <0.01 | 0.10 | <0.01 | 0.74 | 1.00 | 0.64 | 0.17 | 0.028 |
| CA1 | 0.47 | 0.10 | 0.33 | 0.019 | 0.38 | 0.64 | 1.00 | 0.23 | 0.027 |
| Cbl_cor | 0.031 | <0.01 | 0.07 | <0.01 | 0.11 | 0.17 | 0.23 | 1.00 | 0.26 |
| Cbl_Dentncl | <0.01 | <0.01 | <0.01 | <0.01 | 0.05 | 0.028 | 0.027 | 0.26 | 1.00 |

Cbl cerebellum, cor cortex, Dentncl dentate nucleus, F frontal, GY gyrus, MF middle frontal, O occipital, Phip parahippocampal, SU sulcus, Sub subiculum

***Table S16:*** *Comparison of proportional loads of cerebral amyloid angiopathy (CAA) across brain regions [%] in complete subset*
*Complete subset is defined as all cases with completed Aβ staining in the listed nine brain regions. (a) Kruskal-Wallis tests and Dunn's post hoc analysis and (b) Wilcoxon signed-rank test with FDR Benjamini-Hochberg correction for multiple testing, respectively. Regions are indicated with background colors, significant, false discovery rate corrected p-values are labeled in green font color.*

| a) | MF_GY | F_SU | O_GY | O_SU | Phip_GY | Sub | CA1 | Cbl_cor | Cbl_Dentncl |
|---|---|---|---|---|---|---|---|---|---|
| **MF_GY** | 1.0 | 0.25 | 0.68 | 0.24 | 0.69 | 0.76 | 0.95 | 0.43 | 0.47 |
| **F_SU** | 0.25 | 1.0 | 0.46 | 0.83 | 0.43 | 0.34 | 0.3 | 0.76 | 0.11 |
| **O_GY** | 0.68 | 0.46 | 1.0 | 0.34 | 0.94 | 0.83 | 0.69 | 0.72 | 0.32 |
| **O_SU** | 0.24 | 0.83 | 0.34 | 1.0 | 0.34 | 0.3 | 0.24 | 0.68 | 0.1 |
| **Phip_GY** | 0.69 | 0.43 | 0.94 | 0.34 | 1.0 | 0.89 | 0.72 | 0.69 | 0.34 |
| **Sub** | 0.76 | 0.34 | 0.83 | 0.3 | 0.89 | 1.0 | 0.81 | 0.66 | 0.34 |
| **CA1** | 0.95 | 0.3 | 0.69 | 0.24 | 0.72 | 0.81 | 1.0 | 0.46 | 0.47 |
| **Cbl_cor** | 0.43 | 0.76 | 0.72 | 0.68 | 0.69 | 0.66 | 0.46 | 1.0 | 0.24 |
| **Cbl_Dentncl** | 0.47 | 0.11 | 0.32 | 0.1 | 0.34 | 0.34 | 0.47 | 0.24 | 1.0 |

| b) | MF_GY | F_SU | O_GY | O_SU | Phip_GY | Sub | CA1 | Cbl_cor | Cbl_Dentncl |
|---|---|---|---|---|---|---|---|---|---|
| **MF_GY** | 1.00 | 0.67 | 0.81 | 0.26 | 0.61 | 0.92 | 0.77 | 0.25 | 0.38 |
| **F_SU** | 0.67 | 1.00 | 0.89 | 0.22 | 0.92 | 0.49 | 0.44 | 0.62 | 0.22 |
| **O_GY** | 0.81 | 0.89 | 1.00 | 0.29 | 0.81 | 0.74 | 0.84 | 0.30 | 0.25 |
| **O_SU** | 0.26 | 0.22 | 0.29 | 1.00 | 0.25 | 0.25 | 0.42 | 0.65 | 0.22 |
| **Phip_GY** | 0.61 | 0.92 | 0.81 | 0.25 | 1.00 | 0.74 | 0.81 | 0.33 | 0.25 |
| **Sub** | 0.92 | 0.49 | 0.74 | 0.25 | 0.74 | 1.00 | 0.81 | 0.49 | 0.25 |
| **CA1** | 0.77 | 0.44 | 0.84 | 0.42 | 0.81 | 0.81 | 1.00 | 0.30 | 0.38 |
| **Cbl_cor** | 0.25 | 0.62 | 0.30 | 0.65 | 0.33 | 0.49 | 0.30 | 1.00 | 0.25 |
| **Cbl_Dentncl** | 0.38 | 0.22 | 0.25 | 0.22 | 0.25 | 0.25 | 0.38 | 0.25 | 1.00 |

Cbl cerebellum, cor cortex, Dentncl dentate nucleus, F frontal, GY gyrus, MF middle frontal, O occipital, Phip parahippocampal, SU sulcus, Sub subiculum

***Table S17:*** *Comparison of proportional loads of small dense plaques across brain regions [%] in complete subset*
*Complete subset is defined as all cases with completed Aβ staining in the listed nine brain regions. (a) Kruskal-Wallis tests and Dunn's post hoc analysis and (b) Wilcoxon signed-rank test with FDR Benjamini-Hochberg correction for multiple testing, respectively. Regions are indicated with background colors, significant, false discovery rate corrected p-values are labeled in green font color.*

| a) | MF_GY | F_SU | O_GY | O_SU | Phip_GY | Sub | CA1 | Cbl_cor | Cbl_Dentncl |
|---|---|---|---|---|---|---|---|---|---|
| **MF_GY** | 1.0 | 0.92 | 0.12 | 0.34 | 0.17 | 0.12 | 0.21 | 0.21 | 0.68 |
| **F_SU** | 0.92 | 1.0 | 0.17 | 0.45 | 0.21 | 0.12 | 0.26 | 0.27 | 0.57 |
| **O_GY** | 0.12 | 0.17 | 1.0 | 0.7 | 0.9 | 0.9 | 0.9 | 0.9 | 0.12 |
| **O_SU** | 0.34 | 0.45 | 0.7 | 1.0 | 0.84 | 0.55 | 0.84 | 0.84 | 0.17 |
| **Phip_GY** | 0.17 | 0.21 | 0.9 | 0.84 | 1.0 | 0.84 | 0.98 | 0.98 | 0.12 |
| **Sub** | 0.12 | 0.12 | 0.9 | 0.55 | 0.84 | 1.0 | 0.84 | 0.84 | 0.12 |
| **CA1** | 0.21 | 0.26 | 0.9 | 0.84 | 0.98 | 0.84 | 1.0 | 0.98 | 0.12 |
| **Cbl_cor** | 0.21 | 0.27 | 0.9 | 0.84 | 0.98 | 0.84 | 0.98 | 1.0 | 0.12 |
| **Cbl_Dentncl** | 0.68 | 0.57 | 0.12 | 0.17 | 0.12 | 0.12 | 0.12 | 0.12 | 1.0 |

| b) | MF_GY | F_SU | O_GY | O_SU | Phip_GY | Sub | CA1 | Cbl_cor | Cbl_Dentncl |
|---|---|---|---|---|---|---|---|---|---|
| **MF_GY** | 1.00 | 0.86 | 0.12 | 0.15 | 0.10 | 0.20 | 0.34 | 0.86 | 0.15 |
| **F_SU** | 0.86 | 1.00 | 0.15 | 0.20 | 0.15 | 0.43 | 0.63 | 0.86 | 0.12 |
| **O_GY** | 0.12 | 0.15 | 1.00 | 0.34 | 1.00 | 0.92 | 0.86 | 0.86 | 0.10 |
| **O_SU** | 0.15 | 0.20 | 0.34 | 1.00 | 0.86 | 0.86 | 0.92 | 1.00 | 0.10 |
| **Phip_GY** | 0.10 | 0.15 | 1.00 | 0.86 | 1.00 | 0.88 | 0.86 | 0.86 | 0.10 |
| **Sub** | 0.20 | 0.43 | 0.92 | 0.86 | 0.88 | 1.00 | 0.86 | 0.92 | 0.15 |
| **CA1** | 0.34 | 0.63 | 0.86 | 0.92 | 0.86 | 0.86 | 1.00 | 1.00 | 0.20 |
| **Cbl_cor** | 0.86 | 0.86 | 0.86 | 1.00 | 0.86 | 0.92 | 1.00 | 1.00 | 0.10 |
| **Cbl_Dentncl** | 0.15 | 0.12 | 0.10 | 0.10 | 0.10 | 0.15 | 0.20 | 0.10 | 1.00 |

Cbl cerebellum, cor cortex, Dentncl dentate nucleus, F frontal, GY gyrus, MF middle frontal, O occipital, Phip parahippocampal, SU sulcus, Sub subiculum

***Table S18:*** *Explanation of column names of the separate supplementary csv Table S19*
*The supplementary csv table is intended to serve as a template for correlating new cross-regional data with the data collected here on a cohort-specific basis. The first few columns characterize the (sub-)cohort, while the subsequent columns contain the mean or median regional Aβ deposit counts per mm² for each of these sub-cohorts.*

| **Column name** | **Description** |
|---|---|
| label | Given name to describe the cohort subgroup |
| sex | f = female, m = male |
| Age_at_onset, Disease_duration_years, Age_at_death | Stated in years |
| ApoE4 | 0 = no ApoE4 allele, 1 = at least one ApoE4 allele |
| genetics | PSEN1 = familial Alzheimer's disease cases with PSEN1 mutation, Sporadic = Alzheimer's disease case with a reported age of onset >65 years and an inconspicuous whole genome sequencing for Alzheimer's disease-related variants |
| aSyn_type | 0 = no α-synuclein co-pathology, 1 = amygdala predominant α-synunclein co-pathology, 2 = brainstem predominant α-synuclein co-pathology, 3 = disseminated, cortical α-synuclein co-pathology |
| TDP43_pos | 0 = no TDP43 co-pathology detected, 1 = TDP43 co-pathology was detected |
| Statistic | The following columns show the *mean* or *median* values of the cohort |
| n | Shows how many cases in the cohort fulfill the criteria which were defined in the columns before. As not every region was available for every case, the mean or median for a specific region might be calculated with less cases. |
| *Region*_count_*class*_mm2 | Shows the mean or median of how many Aβ deposits from all classes (*all*) or from specific classes, i.e. *cored*, diffuse light (*difflight*), diffuse dense (*diffdense*), *compact*, cerebral amyloid angiopathy (*CAA*) or small dense (*smalldense*) plaques were detected per mm² in the subcohort defined in the columns before, restricted by a reduced availability of stains in some brain regions. Mean and median values were rounded to two decimal places.<br>List of abbreviated brain regions: ['MF_GY', 'F_SU', 'P_GY', 'ST_GY', 'T_SU', 'MT_GY', 'Insula', 'O_GY', 'O_SU', 'Phip_GY', 'Ent_GY', 'Amyg', 'Cla', 'Put', 'Tha_MNcl', 'Tha_VLNcl', 'Sub', 'CA1', 'CA2', 'CA3', 'CA4', 'Cbl_cor', 'Cbl_Ncl']<br>List of abbreviated classes: ['all', 'cored', 'difflight', 'diffdense', 'compact', 'CAA', 'smalldense'] |

Amyg amygdala, Cbl cerebellum, Cla claustrum, cor cortex, Dentncl dentate nucleus, Ent entorhinal, F frontal, GY gyrus, MF middle frontal, MNcl medial nucleus, MT middle temporal, O occipital, P parietal, Phip parahippocampal, Put putamen, ST superior temporal, SU sulcus, Sub subiculum, T temporal, Thal thalamus, VLNcl ventrolateral nucleus

**Supplementary Figures**

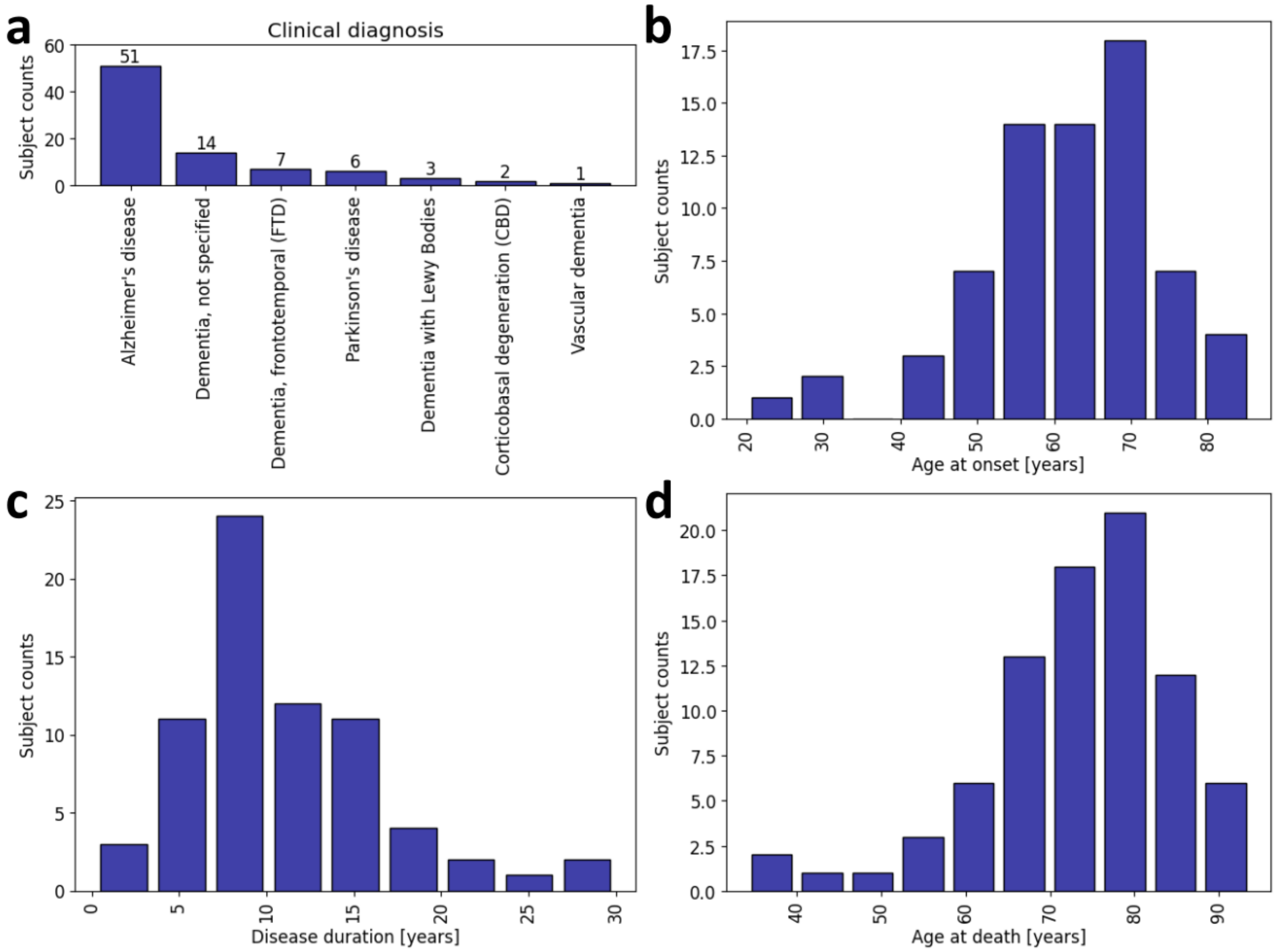


***Fig. S1:*** *Overview of the study cohort*

*Histograms show the distribution of clinical diagnoses, age at symptom onset, disease duration and age at death of the study cohort.*

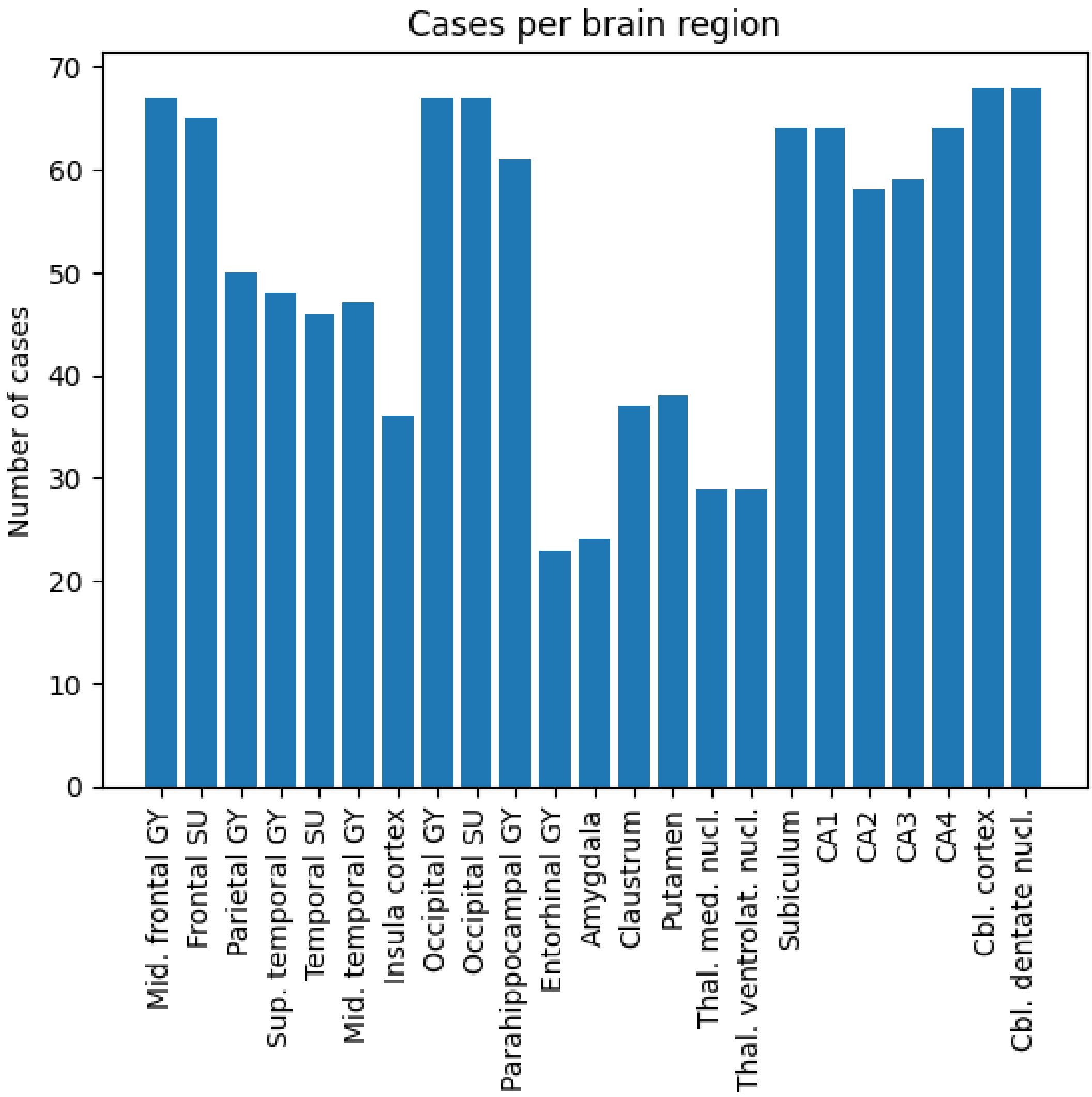


***Fig. S2:*** *Staining availability*

*Histogram of how many cases were available for each brain region. CA cornu ammonis (hippocampus), Cbl. cerebellum, GY gyrus, med. Medial, mid. middle, nucl. nucleus, SU sulcus, Sup. superior, Thal thalamus, ventrolat. Ventrolateral*

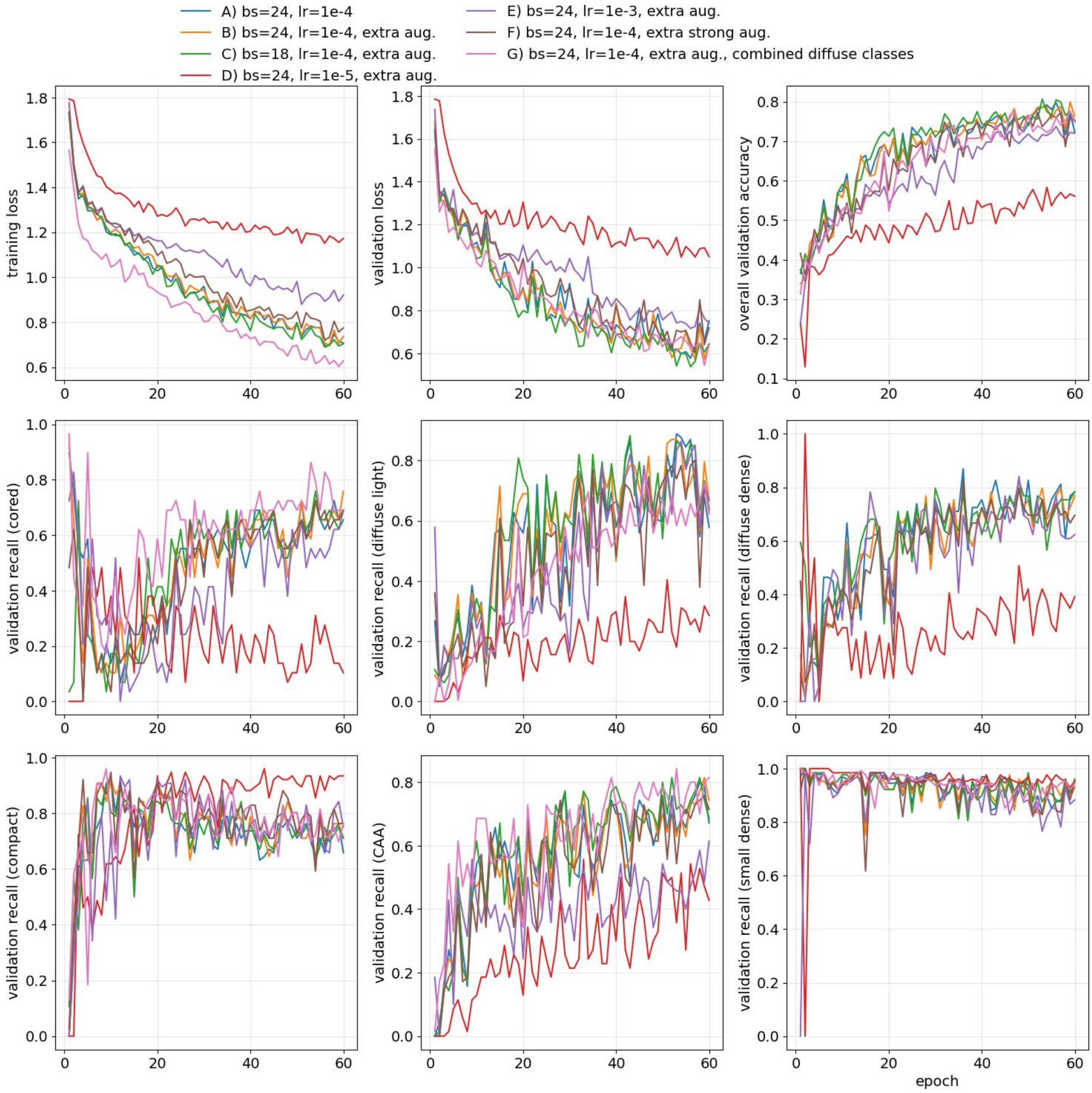


***Fig. S3:*** *Convolutional neural network optimization*

*General and class-wise model performances during training, tested on the validation set, which is not equal to the test set. The x-axis shows the number of epochs. Moderate data augmentation with torchvision (version 0.18.1) included random rotation, horizontal and vertical flip, color jitter (transforms.ColorJitter (brightness=0.1, contrast=0.1, saturation=0.1, hue=0.02)), and further low-grade geometric augmentations (transforms.RandomAffine (0, translate=(0.05, 0.05), scale=(0.95, 1.05), shear=10)). Extra data augmentation (extra aug.) means that underrepresented deposit classes, e.g. cored plaques and CAA, have a higher probability to pass additional data augmentation (transforms.ColorJitter (brightness=0.1, contrast=0.2, saturation=0.2, hue=0.02); transforms.RandomAffine (0, translate=(0.08, 0.08), scale=(0.9, 1.1), shear=10)) to increase the variability in minority classes. Strong data augmentation (strong aug.) included transforms.ColorJitter (brightness=0.2, contrast=0.2, saturation=0.2, hue=0.02) and transforms.RandomAffine (0, translate=(0.05, 0.05), scale=(0.9, 1.1), shear=12). In model G, the classes diffuse light and diffuse dense deposits were combined as one class, here shown in the central plot for diffuse light plaques. Model A, B and C underwent a second performance test with the holdout test set for ultimate selection. Finally, model A, showing the highest overall performance and the highest recall for cored plaques in the test set, was selected for further application. Recall (true positives / (true positives + false negatives)), aug. data augmentation, bs batch size, lr learning rate*

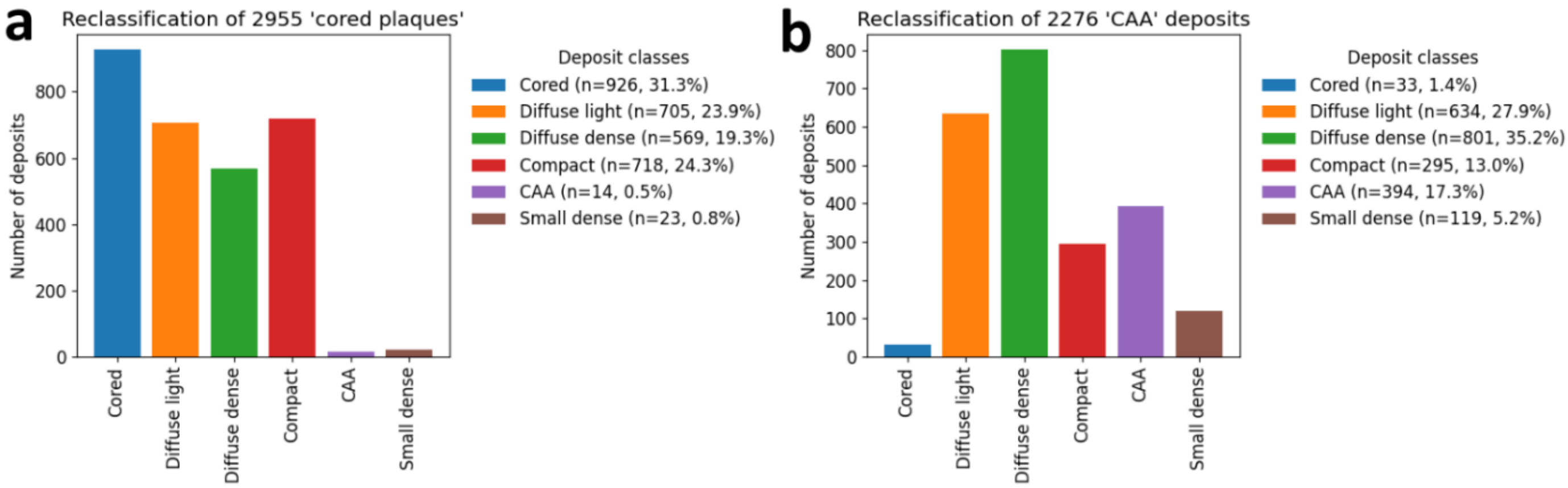


***Fig. S4:*** *Manual reclassification of deposits which were classified as 'cored plaques' or 'CAA'.*

*(a) 2955 Aβ deposits were classified as cored plaques by the convolutional neural network. Color images of these deposits were manually reclassified where necessary, resulting in the distribution presented in the plot. (b) 2276 Aβ deposits were classified as CAA by the model. As before, color images were manually reclassified as presented in the plot. The precision (true positives / (true positives + false positives)) of the model is worse than in the test set, presumably for two reasons: 1) The proportion of cored plaques and CAA in the large application dataset is lower than in the test set. The proportions in the training, validation and test sets were intentionally increased by preferred selection of images with CAA and cored plaques to enable the model to learn these underrepresented classes more robustly. 2) The large application dataset includes numerous plaques that are difficult to classify even by humans. A fraction of these plaques was misclassified as 'cored' or 'CAA' contrary to the relatively strict inclusion criteria of these two classes for human raters. These results therefore do not contradict the results from the test set, but they underscore the importance of manual verification for these two specific classes. CAA cerebral amyloid angiopathy*

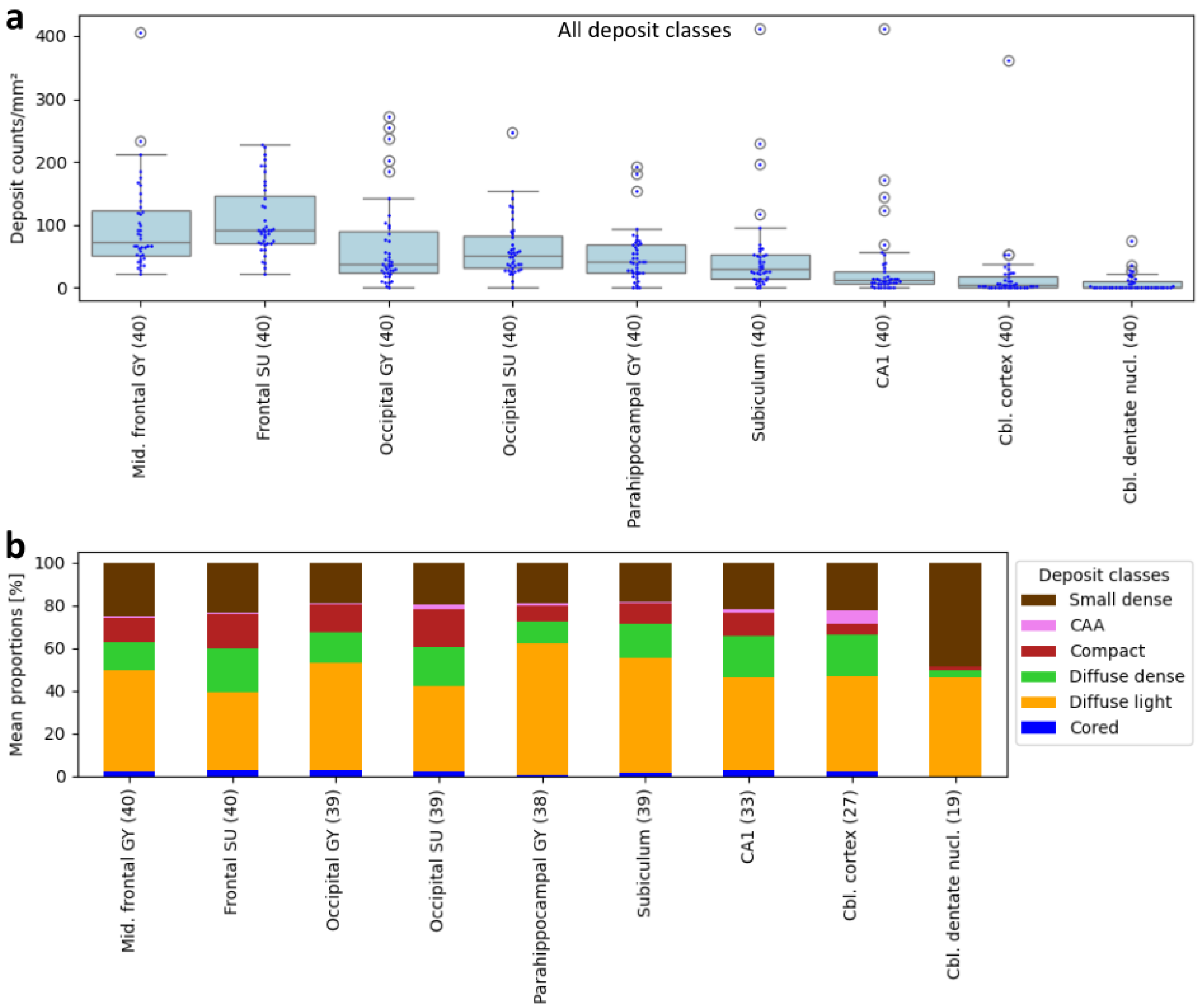


***Fig. S5:*** *Aβ deposit classes across regions with a data subset*

*These plots correspond to the main Fig. 3a and b but with a subset of 40 subjects that have complete data for the nine brain regions listed here. The idea is to get a visual impression if there is a bias in the whole cohort because of the missing data points. (a) Deposit counts per mm² summing up all deposit classes per region. (b) Mean proportions of deposit classes [%] in each region. The numbers in brackets behind region names indicate how many cases are included in the above plot. Numbers in plot b can be lower as proportions of plaques cannot be calculated in images with zero detected deposits. CAA cerebral amyloid angiopathy, Cbl. cerebellum, GY gyrus, med. medial, mid. middle, nucl. nucleus, SU sulcus*

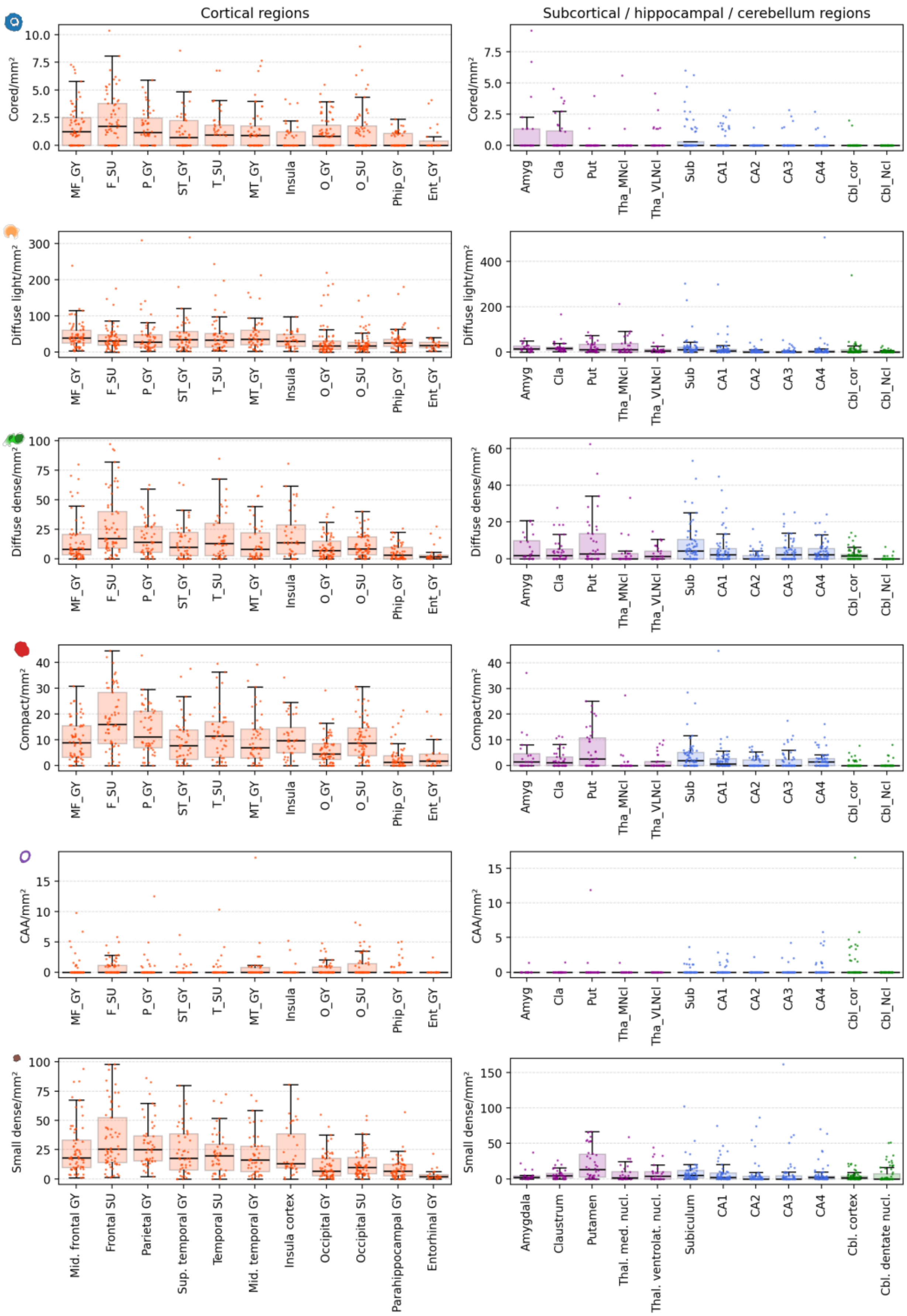


***Fig. S6:*** *Aβ deposit counts per mm² separately for each deposit class and brain region*

*Cortical regions are shown on the left side, non-cortical regions on the right. Amyg amygdala, CAA cerebral amyloid angiopathy, Cbl cerebellum, Cla claustrum, cor cortex, Dentncl dentate nucleus, Ent entorhinal, F frontal, GY gyrus,*

*med. medial, mid. middle, MF middle frontal, MNcl medial nucleus, MT middle temporal, nucl. nucleus, O occipital, P parietal, Phip parahippocampal, Put putamen, ST superior temporal, SU sulcus, Sub subiculum, Sup. superior, T temporal, Thal thalamus, vetrolat. Ventrolateral, VlNcl ventrolateral nucleus*

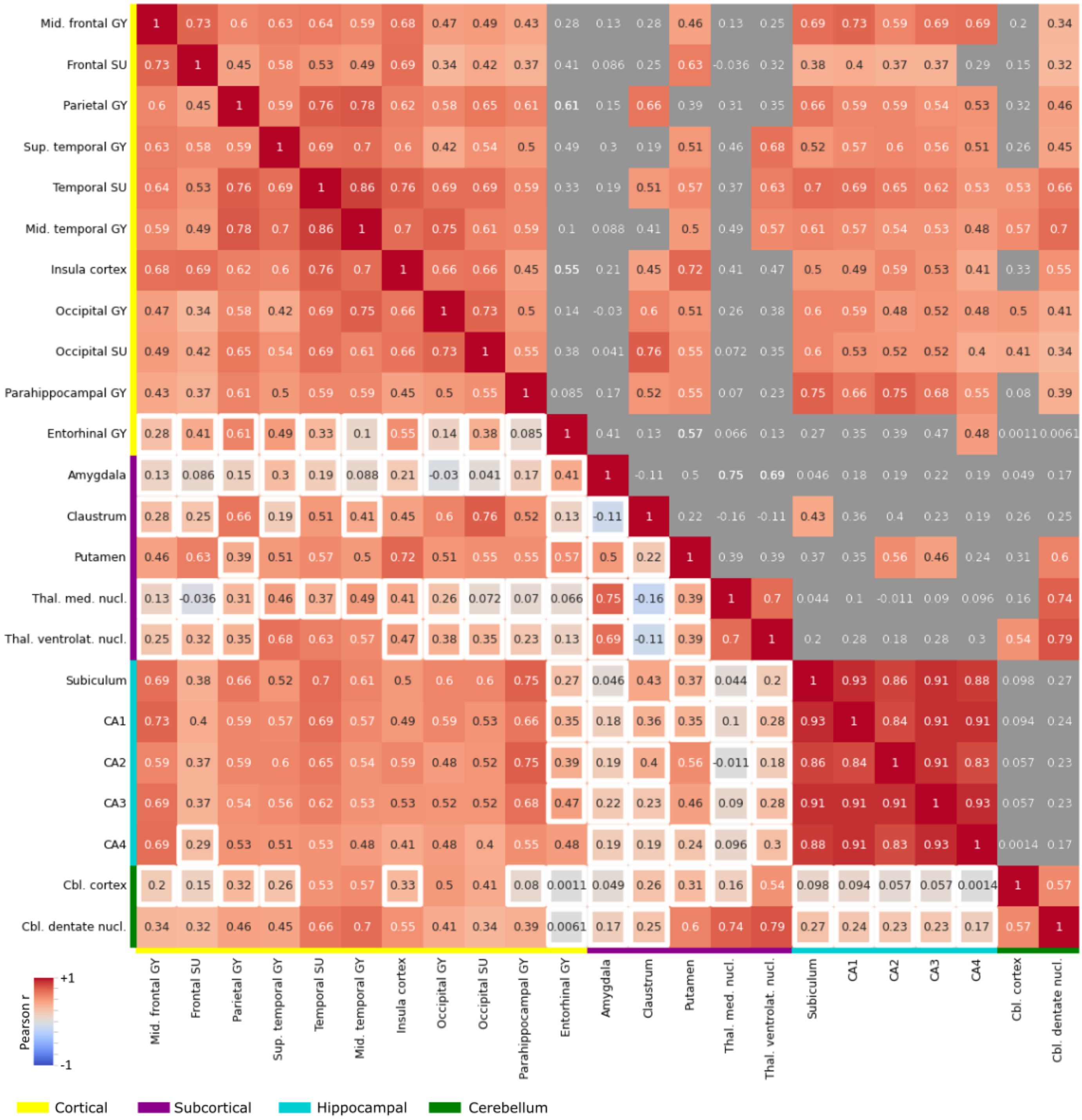


***Fig. S7:*** *Pearson correlation between regional deposit counts of all classes together (/mm²)*

*This Fig. matches the large triangle heatmap in main Fig. 3c. Non-significant correlation coefficients are shown with gray background color in the upper half or white frame in the lower half. Corresponding false discovery rate corrected p-values are presented in the SFig. below. CA cornu ammonis (hippocampus), Cbl. cerebellum, GY gyrus, med. medial, mid. middle, nucl. nucleus, SU sulcus, Sup. superior, Thal thalamus, ventrolat. ventrolateral*

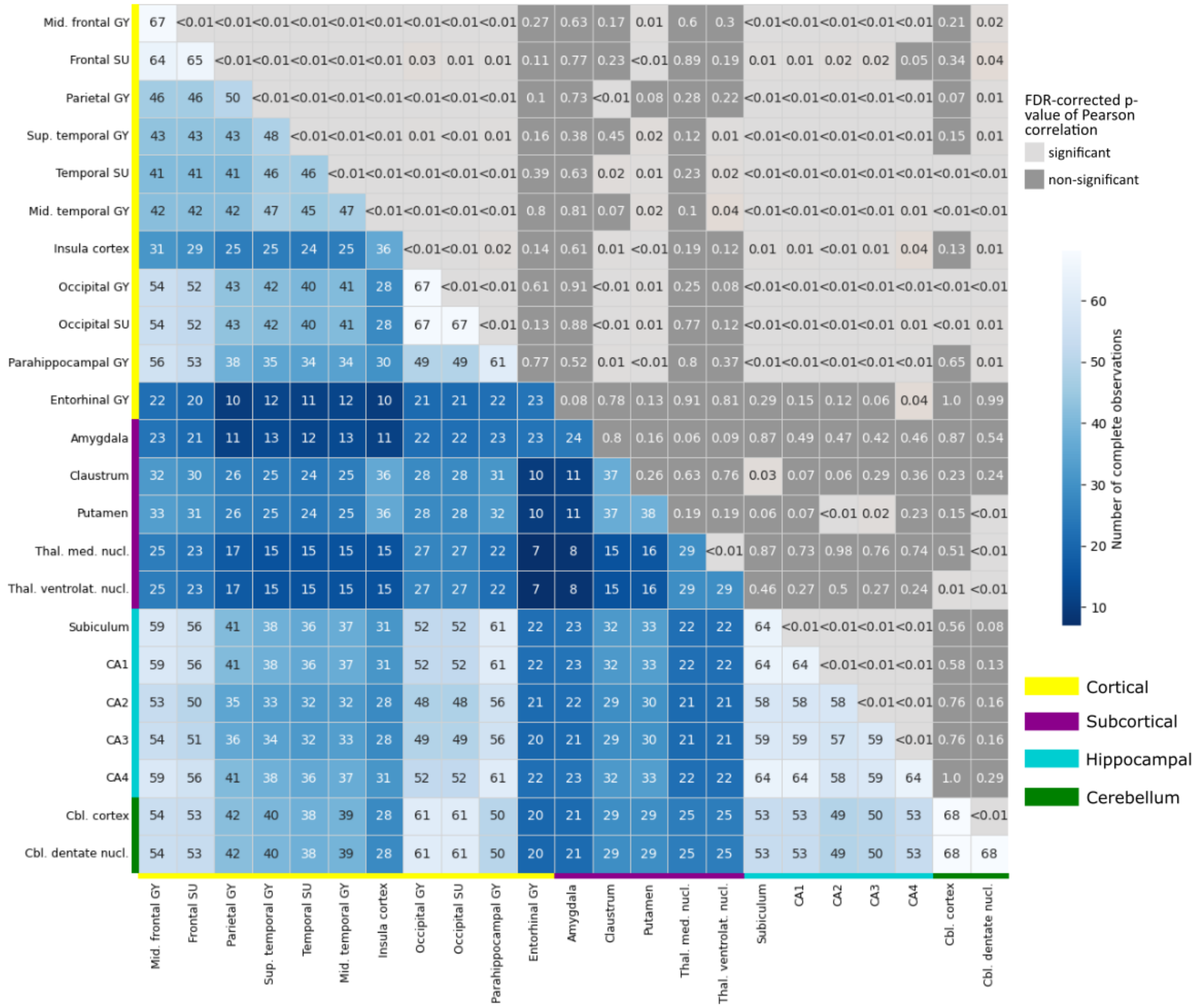


***Fig. S8:*** *P-values and n of the above Pearson correlation analysis*

*Upper half: False discovery rate corrected p-values corresponding to the correlation analysis of Aβ deposit counts between brain regions in the previous figure. Lower half: Number of available observations per region combination. CA cornu ammonis (hippocampus), Cbl. cerebellum, GY gyrus, med. medial, mid. middle, nucl. nucleus, SU sulcus, Sup. superior, Thal thalamus, ventrolat. ventrolateral*

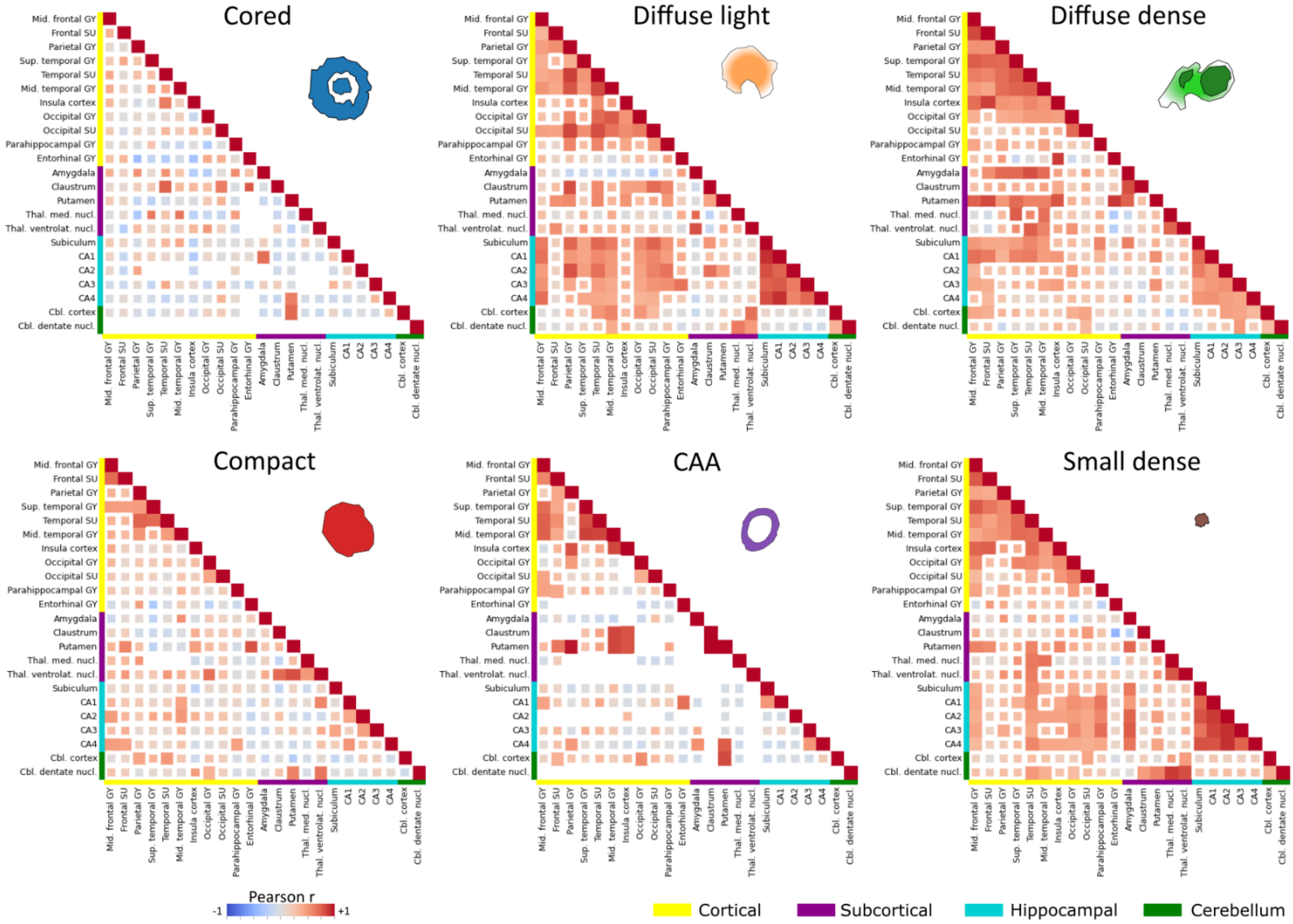


***Fig. S9:*** *Class-wise Aβ plaque correlations between brain regions*

*The heatmaps show Pearson correlation coefficients between brain regions regarding plaque counts per mm² for each Aβ deposit class separately. Non-significant correlations are labeled with a white frame. Completely white cells indicate less than three datapoints for this correlation. The subplots for diffuse light, diffuse dense and small dense plaques are also shown in main Fig. 3c. CA cornu ammonis (hippocampus), Cbl. cerebellum, GY gyrus, med. medial, mid. middle, nucl. nucleus, SU sulcus, Sup. superior, Thal thalamus, ventrolat. ventrolateral*

**Cortical regions (part 1)**

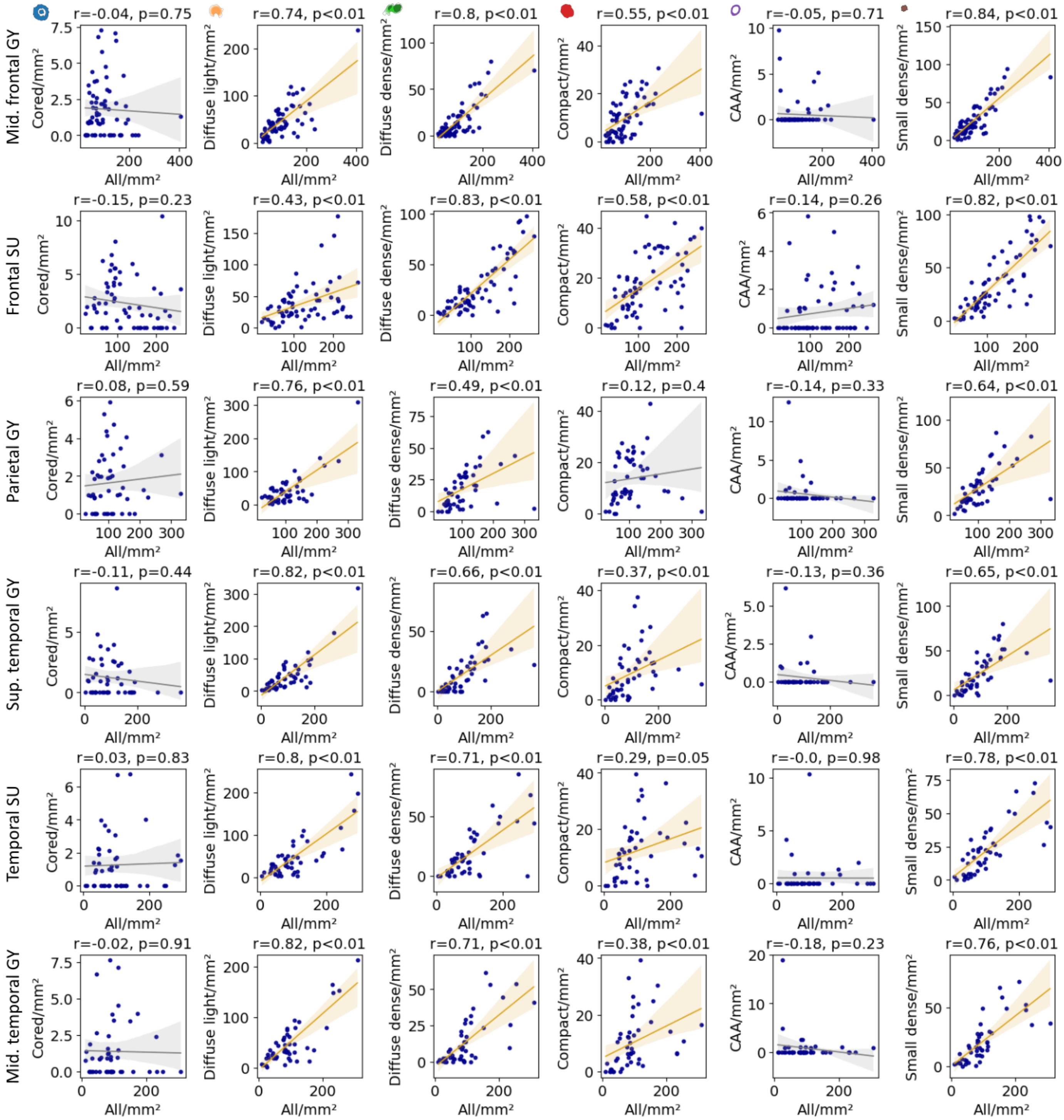


***Fig. S10:*** *Regional associations between Aβ deposit classes and total Aβ load*

*Pearson correlation coefficients r between deposit classes (counts/mm²) and total Aβ plaque loads (All/mm²) for each cortical brain region separately. Significant correlations are shown with yellow regression lines, non-significant correlations are grayed out (without false discovery rate correction). This Fig. is continued in the following Fig. CAA cerebral amyloid angiopathy, GY gyrus, Mid. middle, SU sulcus, Sup. superior*

**Cortical regions (part 2)**

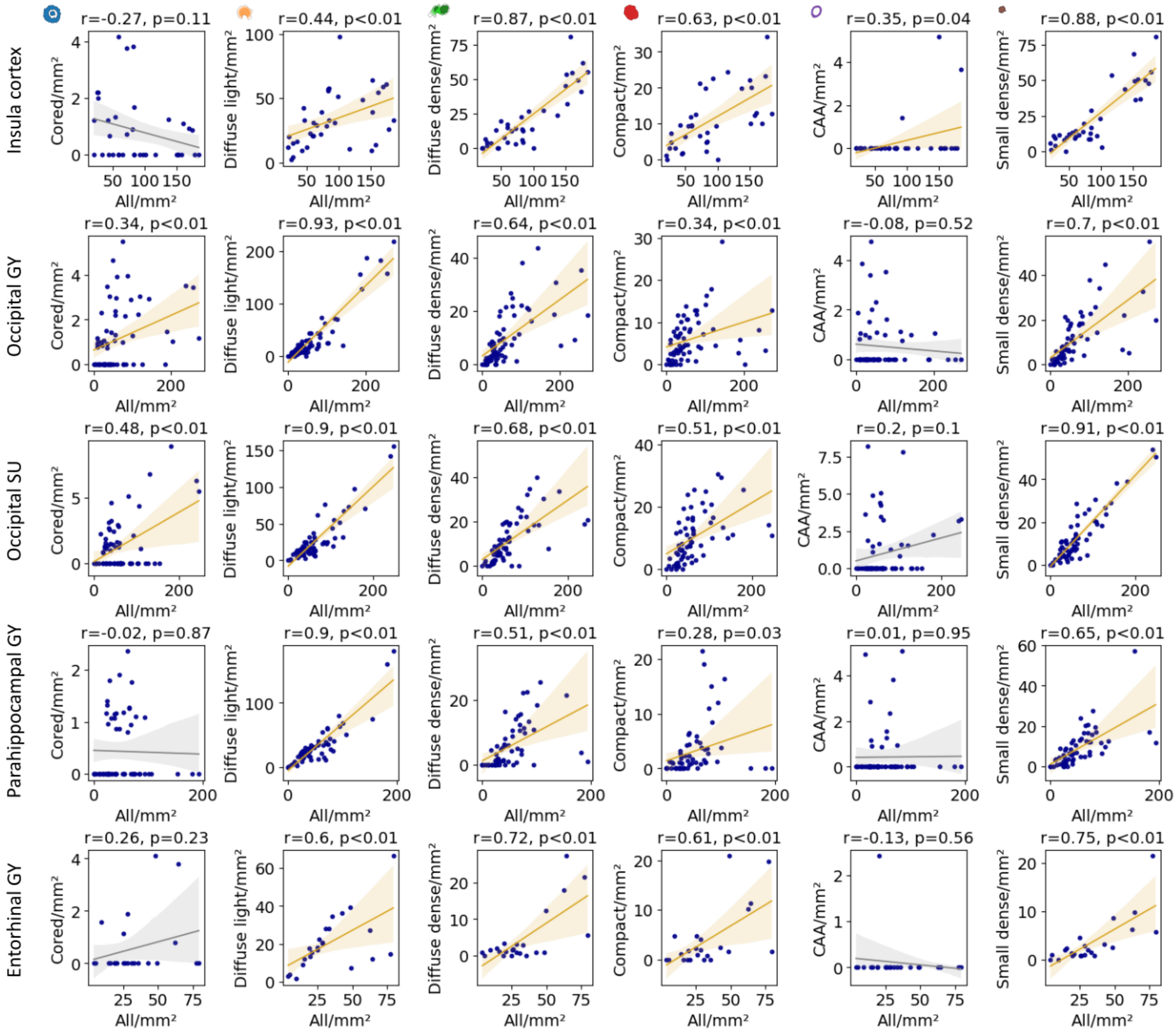


***Fig. S11:*** *This Fig. continues the preceding Fig.*

*Pearson correlation coefficients r between deposit classes (counts/mm²) and total Aβ plaque loads (All/mm²) for each cortical brain region separately. Significant correlations are shown with yellow regression lines, non-significant correlations are grayed out (without false discovery rate correction). CAA cerebral amyloid angiopathy, GY gyrus, Med. medial, SU sulcus, Sup. superior*

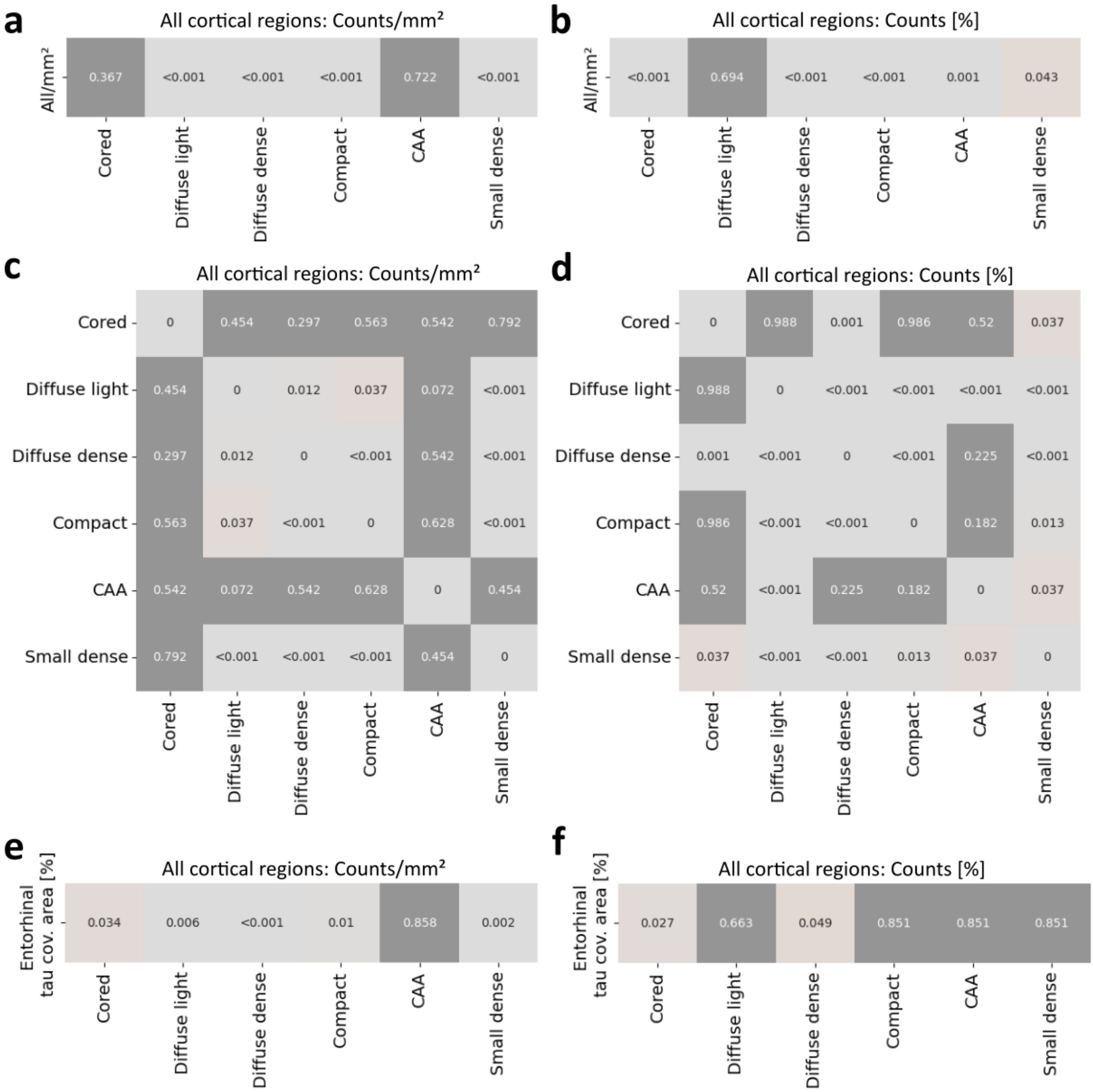


**Fig. S12:** *False discovery rate corrected p-values of Pearson correlation analyses*

*Significant p-values are highlighted in light gray. (a) P-values for main Fig. 4a, correlating cortical plaque counts (/mm²) with the overall Aβ plaque load. (b) P-values for main Fig. 4c, correlating the proportions of cortical plaque loads (%) with the overall Aβ plaque load. (c) P-values for main Fig. 4e, correlating counts of plaque classes (/mm²) across cortical regions. (d) P-values for main Fig. 4g, correlating proportional loads of plaque classes (%) across cortical regions. (e) P-values for main Fig. 5a, correlating the entorhinal tau load (% of covered area) with the counts of Aβ plaques across cortical regions. (f) P-values for Fig. S15a, correlating the entorhinal tau load (% of covered area) with the proportional load of Aβ deposit classes (%) across cortical regions. CAA cerebral amyloid angiopathy, cov. covered*

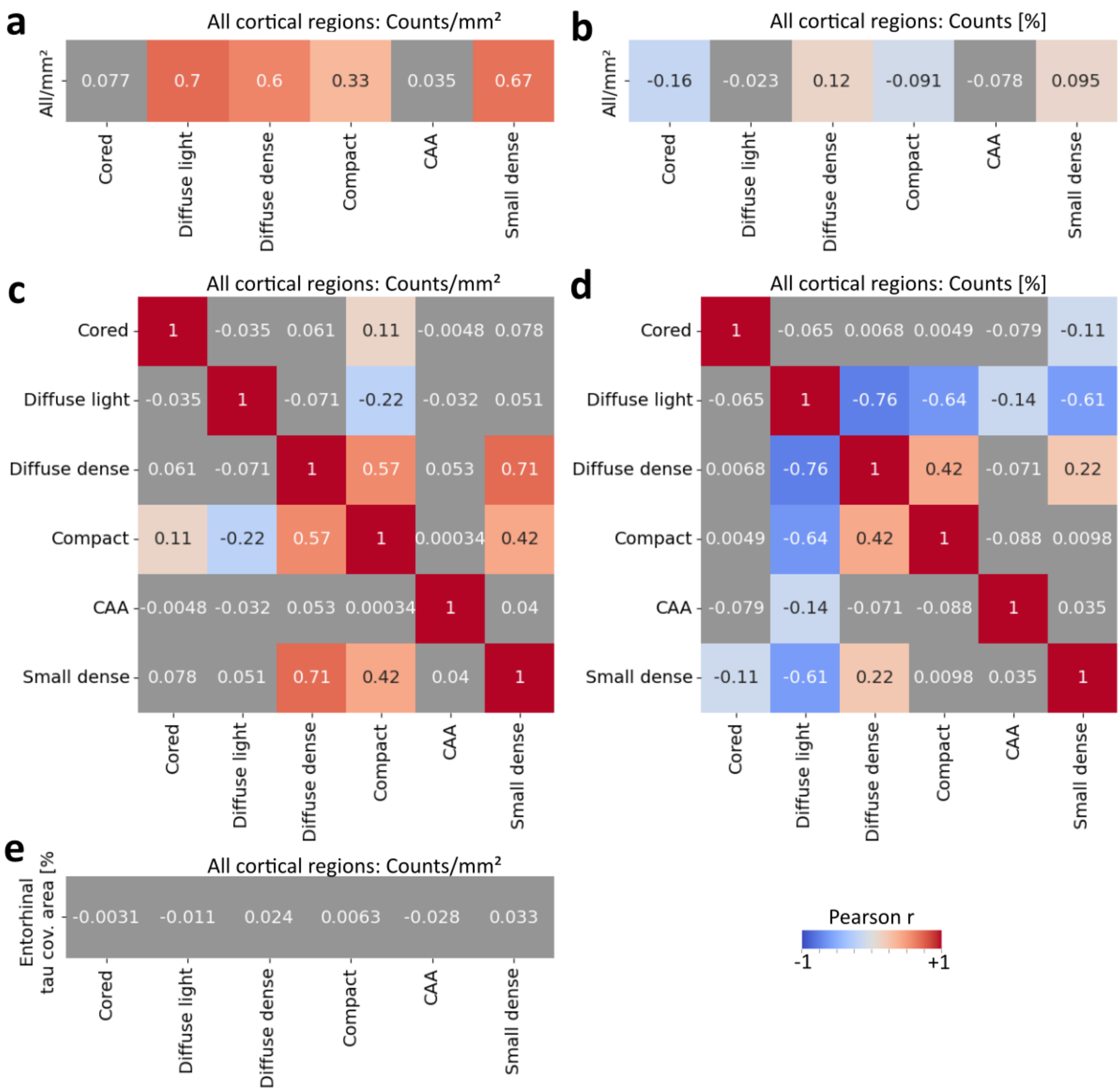


***Fig. S13:*** *Associations between deposit classes with correction for patient ID*

*Correlation coefficients are presented in parallel to Fig. 4a, c, e and g, and Fig. 5a with additional correction for patient ID. Non-significant correlations are grayed out in the heatmaps. (a) Pearson correlation coefficients r across cortical regions between the total count of deposits (All/mm²) and counts/mm² of individual deposit classes. Statistics were corrected for individual region names and patient IDs by linear regression; the significance level was set to <0.05 after false discovery rate correction. (b) Pearson correlation coefficients r across cortical regions between the total count of deposits (All/mm²) and the proportion of individual deposit classes in percent. Statistics in parallel to (a). (c) Heatmap showing correlations between plaque classes (counts/mm²) across cortical regions. Statistics in parallel to (a). (d) Heatmap showing correlations between plaque class proportions [%] across cortical regions. Statistics in parallel to (a). (e) Heatmaps showing correlations between the tau covered area in the entorhinal cortex (%) and counts/mm² of individual deposit classes. Statistics in parallel to (a). CAA cerebral amyloid angiopathy, cov. covered*

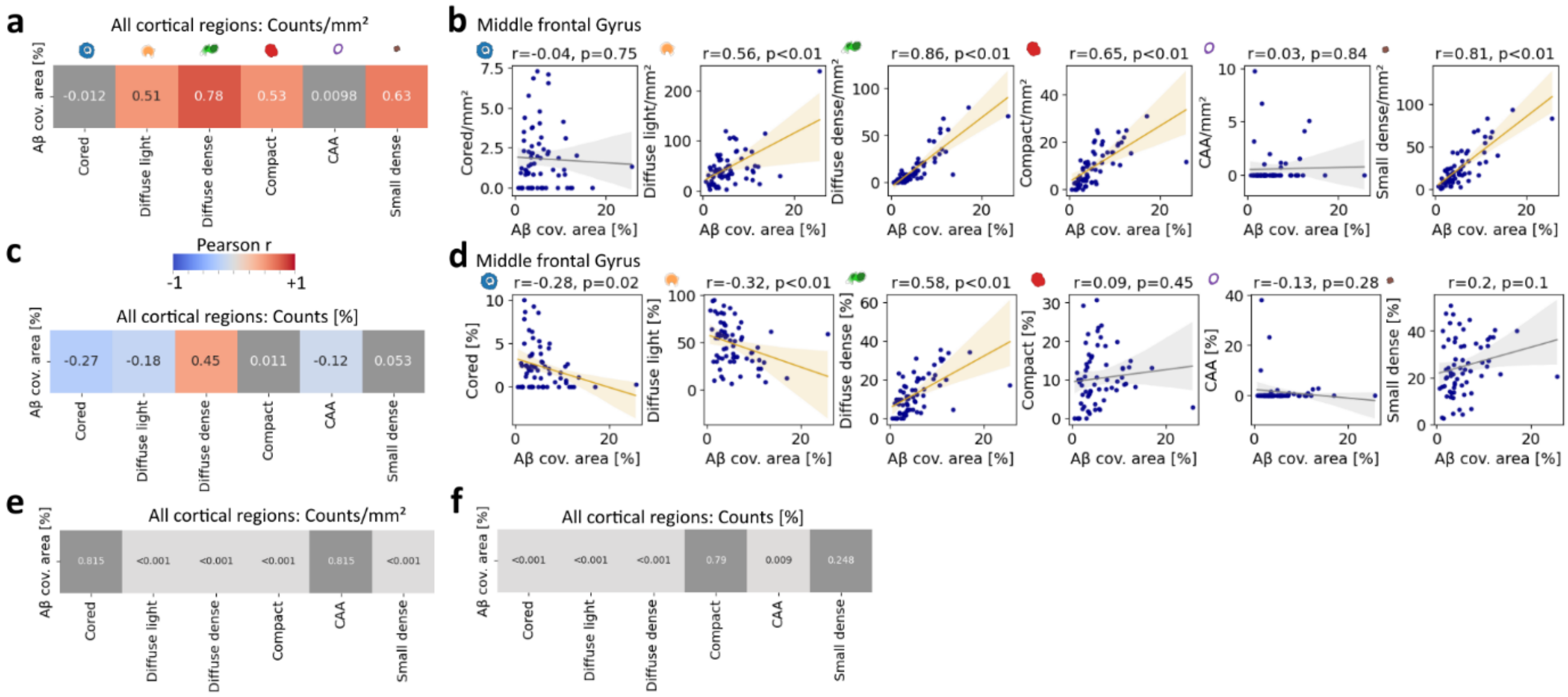


***Fig. S14:*** *Associations between deposit classes and Aβ covered area*

*Non-significant correlations are grayed out in heatmaps and scatter plots. (a) Pearson correlation coefficients r across cortical regions between the total Aβ covered area [%] and counts/mm² of individual deposit classes. Statistics were corrected for individual region names by linear regression; the significance level was set to <0.05 after false discovery rate correction. (b) Scatterplots illustrate the correlation analysis in parallel to (a) using the middle frontal gyrus as an example region. (c) Pearson correlation coefficients r across cortical regions between the total Aβ covered area (%) and the proportion of individual deposit classes in percent. Statistics in parallel to (a). (d) Scatterplots illustrate the correlation analysis in parallel to (c) using the middle frontal gyrus as an example region. (e) Corresponding p-values to subplot (a). (f) Corresponding p-values to subplot (c). CAA cerebral amyloid angiopathy, cov. covered*

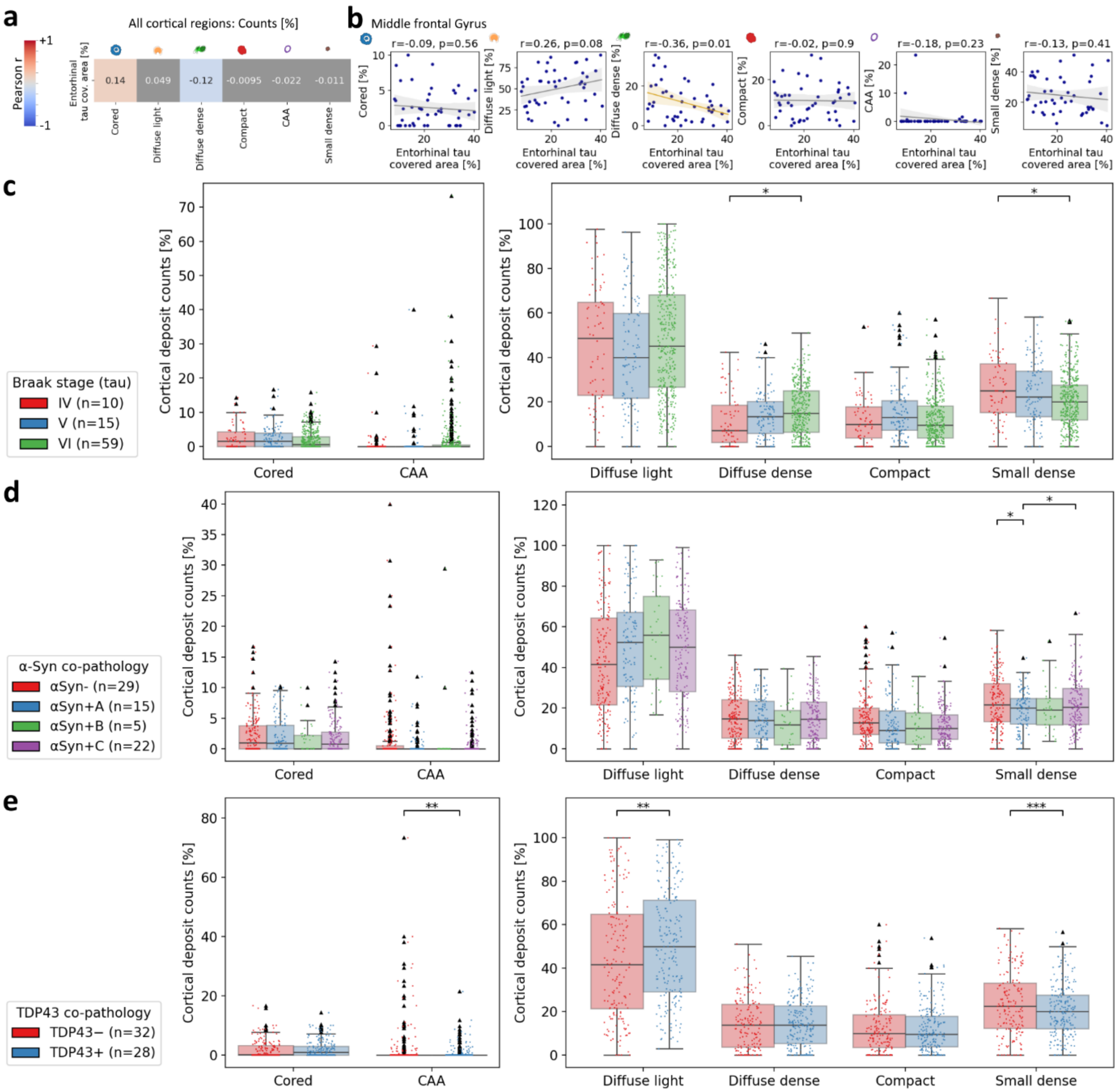


***Fig. S15:** Associations between Aβ deposit classes and other protein deposits*

*This Fig. repeats the analysis from main Fig. 5, but with proportional Aβ plaque loads instead of counts per mm². (a) Pearson correlation coefficients r across cortical regions between the tau covered area in the entorhinal cortex (%) and proportions of individual deposit classes. Statistics were corrected for individual region names by linear regression; the significance level was set to <0.05 after false discovery rate correction. Non-significant correlations are grayed out. (b) Scatterplots illustrate the Pearson correlation analysis in parallel to (a) with tau covered area in the entorhinal cortex on the x-axis and Aβ deposit proportions in the middle frontal gyrus on the y-axis. (c) Comparison of cortical Aβ deposit classes between cases with different Braak and Braak (tau) stages. (d) Comparison of cortical Aβ deposit classes between aSyn co-pathology groups. (e) Comparison between TDP43 co-pathology groups. In (c), (d) and (e), groups were compared with multiple linear regression correcting for ApoE genotype, age at death, sex, region names, and false discovery rate correction for multiple testing. The numbers (n=) indicate how many cases were included in the scatter dots. In the linear regression analyses, individual cases with incomplete data for the covariates were excluded. CAA cerebral amyloid angiopathy, aSyn- no aSyn co-pathology, aSyn+A amygdala predominant aSyn co-pathology, aSyn+B brainstem predominant aSyn co-pathology, aSyn+C cortical disseminated aSyn co-pathology*

**Cortical regions (part 1)**

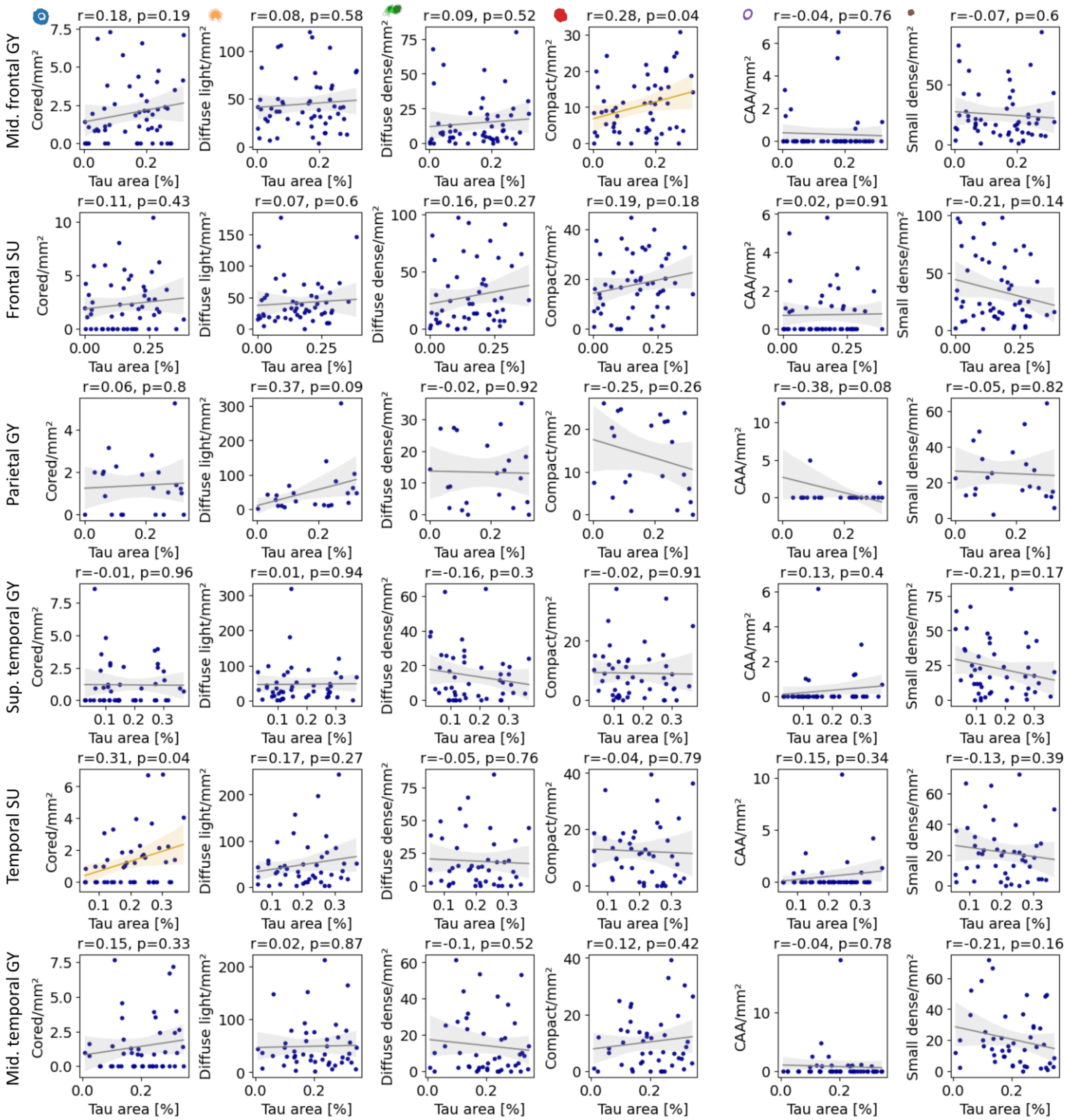


***Fig. S16:*** *Correlations between Aβ and tau load within cortical regions*

*Scatterplots illustrate the Pearson correlation analysis between the tau covered area (%) of the specified brain region (row) and the density of each plaque class (/mm²) in the same region. Significant correlations are shown with yellow regression lines, non-significant correlations are grayed out (without false discovery rate correction). This Fig. is continued in the following Fig. CAA cerebral amyloid angiopathy, GY gyrus, Mid. middle, SU sulcus, Sup. superior*

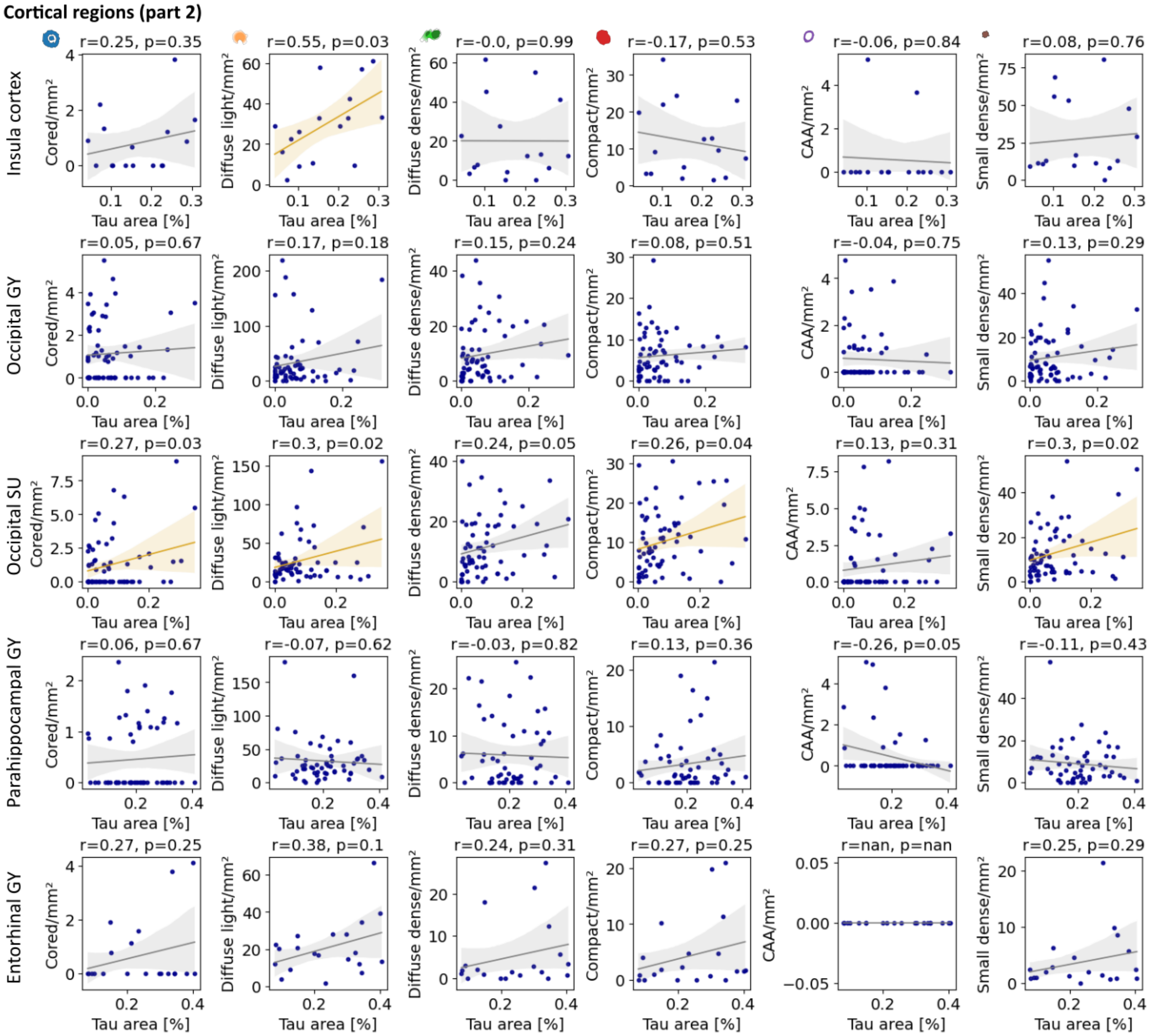


***Fig. S17:*** *This Fig. continues the preceding Fig.*

*Correlations between Aβ and tau load within cortical regions. Scatterplots illustrate the Pearson correlation analysis between the tau covered area (%) of the specified brain region (row) and the density of each plaque class (/mm²) in the same region. Significant correlations are shown with yellow regression lines, non-significant correlations are grayed out (without false discovery rate correction). CAA cerebral amyloid angiopathy, GY gyrus, Med. medial, SU sulcus, Sup. superior*

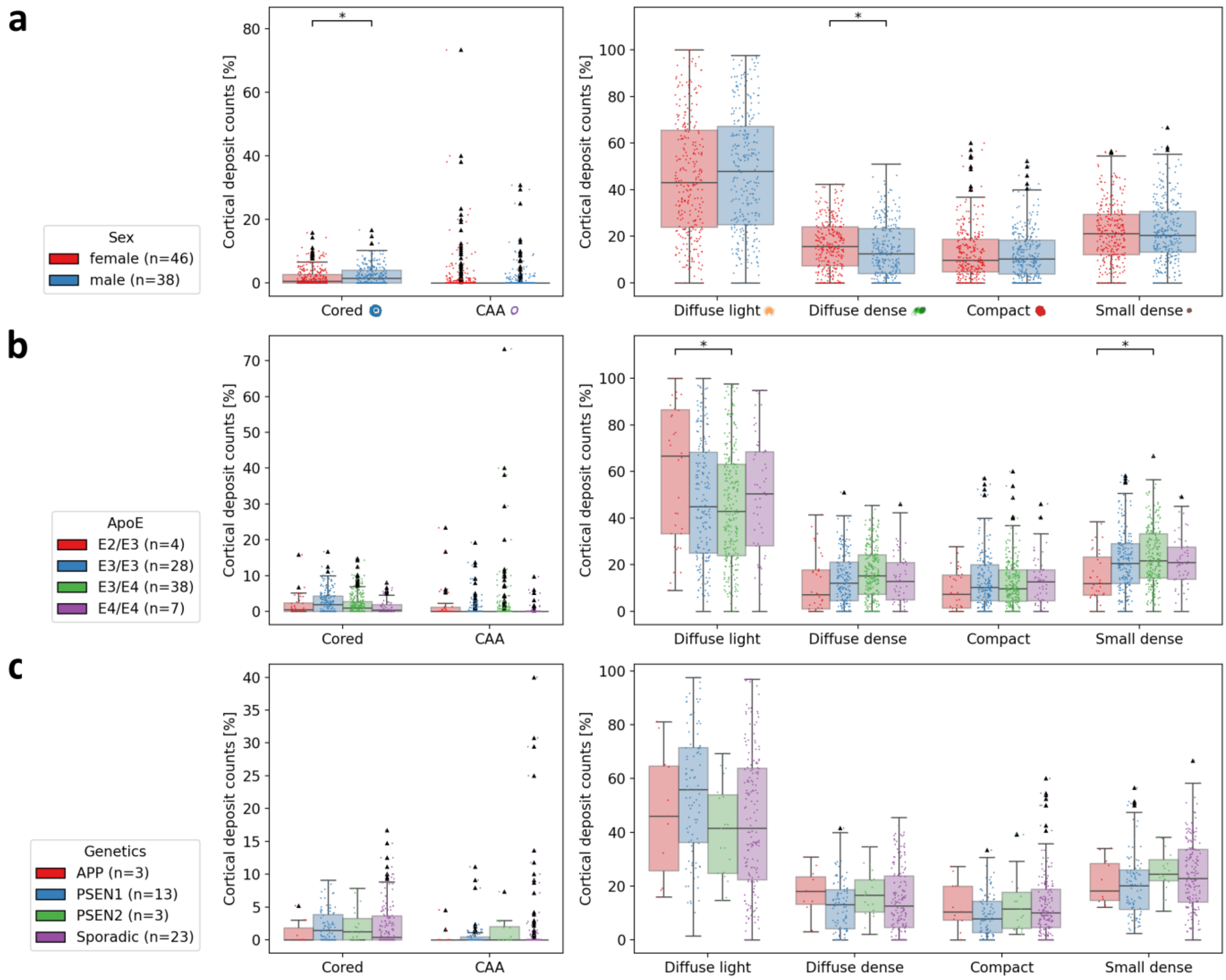


***Fig. S18:** Associations between Aβ deposit classes and genetic factors*

*This Fig. repeats the analysis from main Fig. 6, but with proportional Aβ plaque loads instead of counts per mm². (a) Comparison of cortical Aβ deposit classes between female and male Alzheimer's disease (AD) cases. Groups were compared with multiple linear regression correcting for ApoE genotype, age at death, and region names. (b) Comparison between ApoE genotypes. Groups were compared with multiple linear regression correcting for sex, age at death, and region names. (c) Comparison between familial and sporadic Alzheimer's disease cases. Groups were compared with multiple linear regression correcting for sex, ApoE genotype, and region names. Sporadic cases were defined as AD with a reported age of onset >65 years and an inconspicuous whole genome sequencing for AD-related variants. In (a), (b) and (c), p-values were corrected for multiple testing by respective false discovery rate correction. The numbers (n=) indicate how many cases were included in the scatter dots. In the linear regression analyses, individual cases with incomplete data for the covariates were excluded. CAA cerebral amyloid angiopathy*

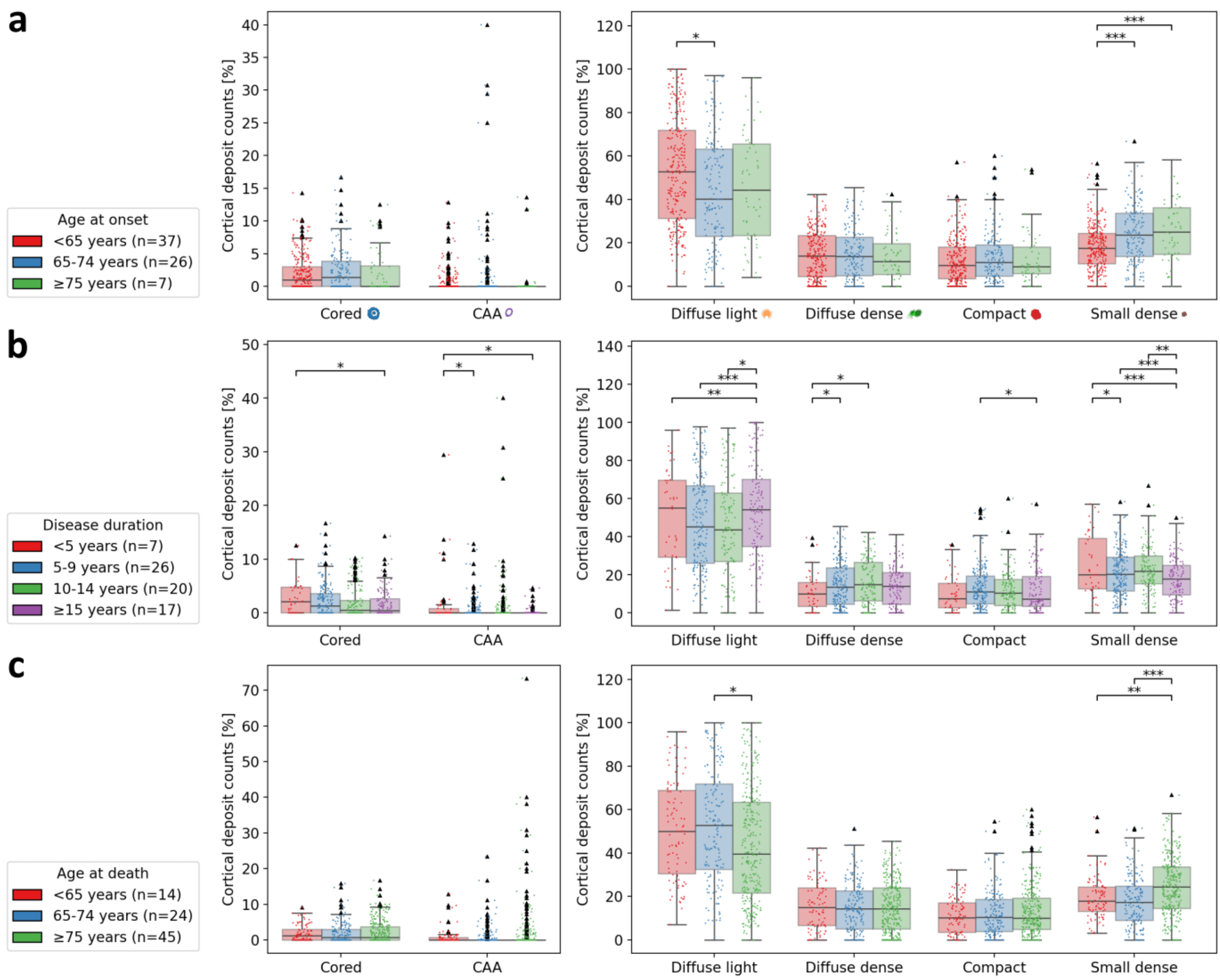


***Fig. S19:*** *Associations between Aβ deposit classes and age*

*This Fig. repeats the analysis from main Fig. 7, but with proportional Aβ plaque loads instead of counts per mm². (a) Comparison of cortical Aβ deposit class densities between early, middle-aged and late onset Alzheimer's disease (AD) cases. Groups were compared with multiple linear regression correcting for ApoE genotype, sex, and region names. (b) Comparison between different disease duration groups. Groups were compared with multiple linear regression correcting for sex, ApoE genotype, age at death, and region names. (c) Comparison between three groups with varying age at death. Groups were compared with multiple linear regression correcting for sex, ApoE genotype, and region names. In (a), (b) and (c), p-values were corrected for multiple testing by respective false discovery rate correction. The numbers (n=) indicate how many cases were included in the scatter dots. In the linear regression analyses, individual cases with incomplete data for the covariates were excluded. CAA cerebral amyloid angiopathy*

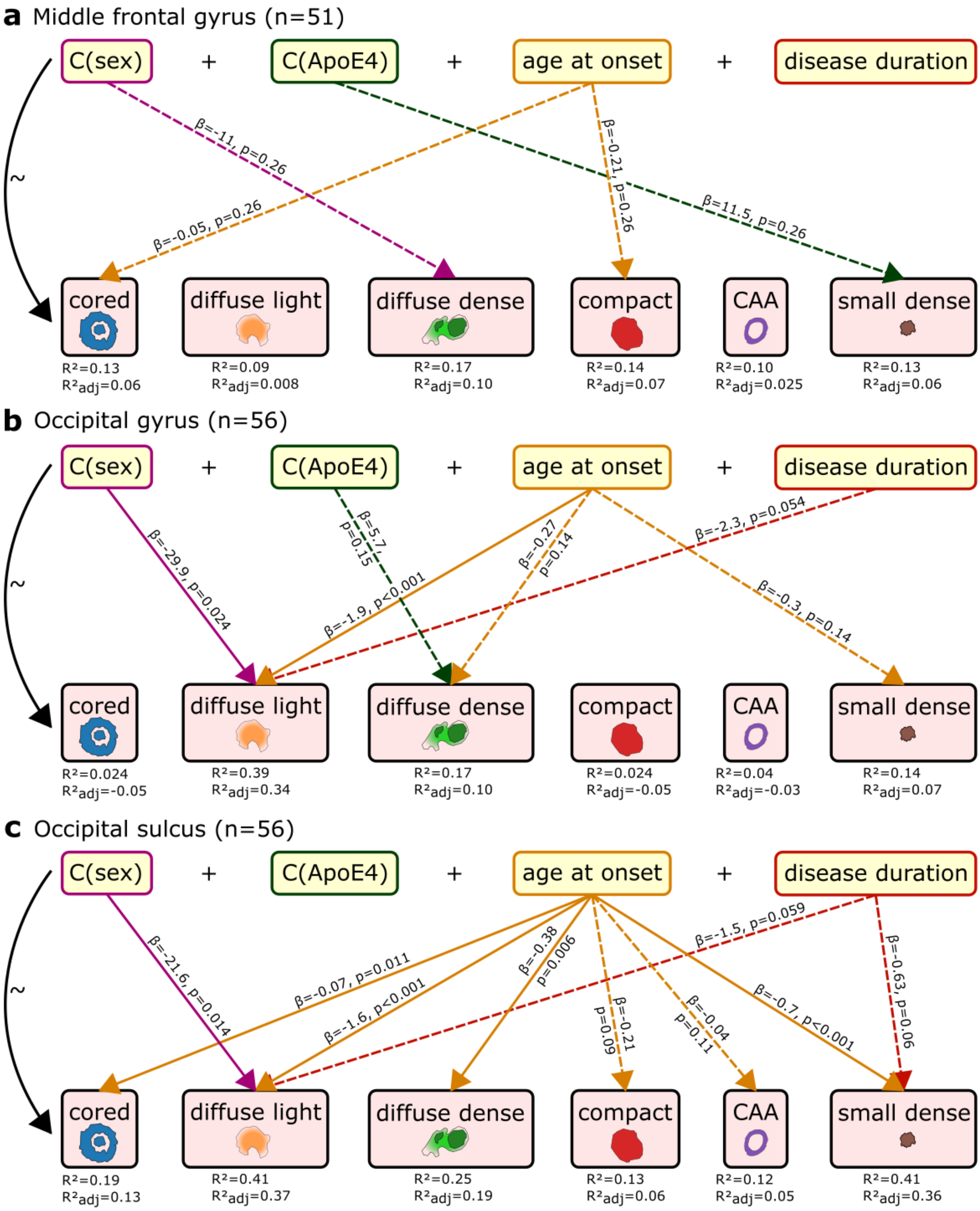


***Fig. S20:*** *Integrative analysis of potential Aβ plaque composition determinants for exemplary brain regions*

*To investigate the associations of various factors (upper line in yellow) with densities (counts/mm²) of the six deposit classes (lower line in red), a multiple linear regression model was created for each deposit class separately for three brain regions (a, b, c). Sex (female vs. male), ApoE4 (no E4 vs. at least one E4 allele), age at onset, and disease duration were independent factors and the deposit classes were the dependent variables, respectively. C() indicates categorical variables. Significant effects after correction for false discovery rate (FDR) due to multiple testing are indicated with solid arrow lines, and associations that were only significant before FDR correction are labeled with dashed lines. Adj. adjusted, CAA cerebral amyloid angiopathy*
